# Predicted effects of observed changes in the mRNA and microRNA transcriptome of lung neutrophils during *S. pneumoniae* pneumonia in mice

**DOI:** 10.1038/s41598-017-11638-7

**Published:** 2017-09-12

**Authors:** John C. Gomez, Hong Dang, Matthew Kanke, Robert S. Hagan, Jason R. Mock, Samir N. P. Kelada, Praveen Sethupathy, Claire M. Doerschuk

**Affiliations:** 1 Center for Airways Disease, University of North Carolina at Chapel Hill, Chapel Hill, NC USA; 2 Cystic Fibrosis/Pulmonary Research and Treatment Center, University of North Carolina at Chapel Hill, Chapel Hill, NC USA; 3 Marsico Lung Institute, University of North Carolina at Chapel Hill, Chapel Hill, NC USA; 4 Department of Genetics, University of North Carolina at Chapel Hill, Chapel Hill, NC USA; 5 Department of Medicine, University of North Carolina at Chapel Hill, Chapel Hill, NC USA

## Abstract

The complex role of neutrophils in modulating the inflammatory response is increasingly appreciated. Our studies profiled the expression of mRNAs and microRNAs (miRs) in lung neutrophils in mice during *S. pneumoniae* pneumonia and performed in depth *in silico* analyses. Lung neutrophils were isolated 24 hours after intratracheal instillation of PBS or *S. pneumoniae,* and differentially expressed (DE) mRNAs and miRs were identified. Lung neutrophils from mice with *S. pneumoniae* pneumonia contained 4127 DE mRNAs, 36% of which were upregulated at least 2-fold. During pneumonia, lung neutrophils increase expression of pattern recognition receptors, receptors for inflammatory mediators, transcription factors including NF-κB and AP-1, Nrf2 targets, cytokines, chemokines and other inflammatory mediators. Interestingly, neutrophils responded to Type I interferons, whereas they both produced and responded to Type II interferon. Expression of regulators of the inflammatory and immune response was verified at the mRNA and protein level. Of approximately 1100 miRs queried, 31 increased and 67 decreased more than 2-fold in neutrophils from *S. pneumoniae* pneumonia. Network analyses of potential DE miR-target DE mRNA interactions revealed candidate key regulatory miRs. Thus, *S. pneumoniae* modulates mRNA and miR expression by lung neutrophils, increasing their ability to respond and facilitating host defense.

## Introduction

During inflammation, neutrophils are recruited from the circulation to sites of injury, where they kill invading pathogens through phagocytosis and release of reactive oxygen species, proteases and other effectors. In recent years, the complexity of the neutrophil response has become increasingly clear. Rather than being simply highly motile phagocytic bags of enzymes, neutrophils are in fact capable of a range of responses that encompass alterations in neutrophil adhesiveness and motility, modulation of cell death and survival pathways, and production of cytokines and other mediators. The active and dynamic neutrophil response during inflammation is associated with changes in gene expression^1, 2^. Neutrophils also express miRs, short single-stranded RNAs that recognize sequences in the 3′ untranslated region (UTR) of mRNAs and cause post-transcriptional silencing of the target mRNA by suppressing protein synthesis and/or inducing mRNA degradation^3^. As with mRNAs, miR expression in neutrophils can be modulated during development, apoptosis, exercise, during infection or after administration of inflammatory mediators such as LPS^4–16^. Transcription factors regulate transcription of mRNAs and microRNAs (miRs) by binding to DNA sequences adjacent to the target genes and directing recruitment of RNA polymerases and other enzymes. Because each transcription factor usually regulates more than one gene, which in turn may modify the expression and function of other downstream genes, transcription factors are key players in determining the host response during infection.

*S. pneumoniae* is the most common pathogen causing community-acquired pneumonia and is associated with a cellular infiltrate composed primarily of neutrophils in the acute stages^17^. In this study, we tested the hypothesis that expression of mRNAs and miRs by neutrophils in the lungs is altered during pneumonia induced by *S. pneumoniae*. Our results presented herein demonstrate that *S. pneumoniae* modulates mRNA and miR expression by lung neutrophils, which likely shape the inflammatory and immune response against this pathogen and subsequent resolution and repair in the lungs. Identifying the changes in mRNAs and miRs induced by this organism provides an unprecedented opportunity to map the signaling pathways that are most critical to the host response to infection. Although studies have addressed changes in gene expression in blood or bone marrow neutrophils following *ex vivo* stimulation, we believe these are the first studies profiling gene expression in neutrophils isolated from lung tissue with and without infection. Network-level characterization of miR-mediated regulation of mRNAs in neutrophils during pneumonia can facilitate the development of improved therapies for fighting infections and diseases in which neutrophils play a critical role.

## Results

Neutrophils were isolated from lung digests 24 h after intratracheal instillation of *S. pneumoniae* or PBS. The results of the analyses profiling both mRNA and miR expression in the same neutrophils are described below, followed by integration of mRNA and miR expression into a signaling network schema.

### mRNA expression profiling

**Figure 1:**
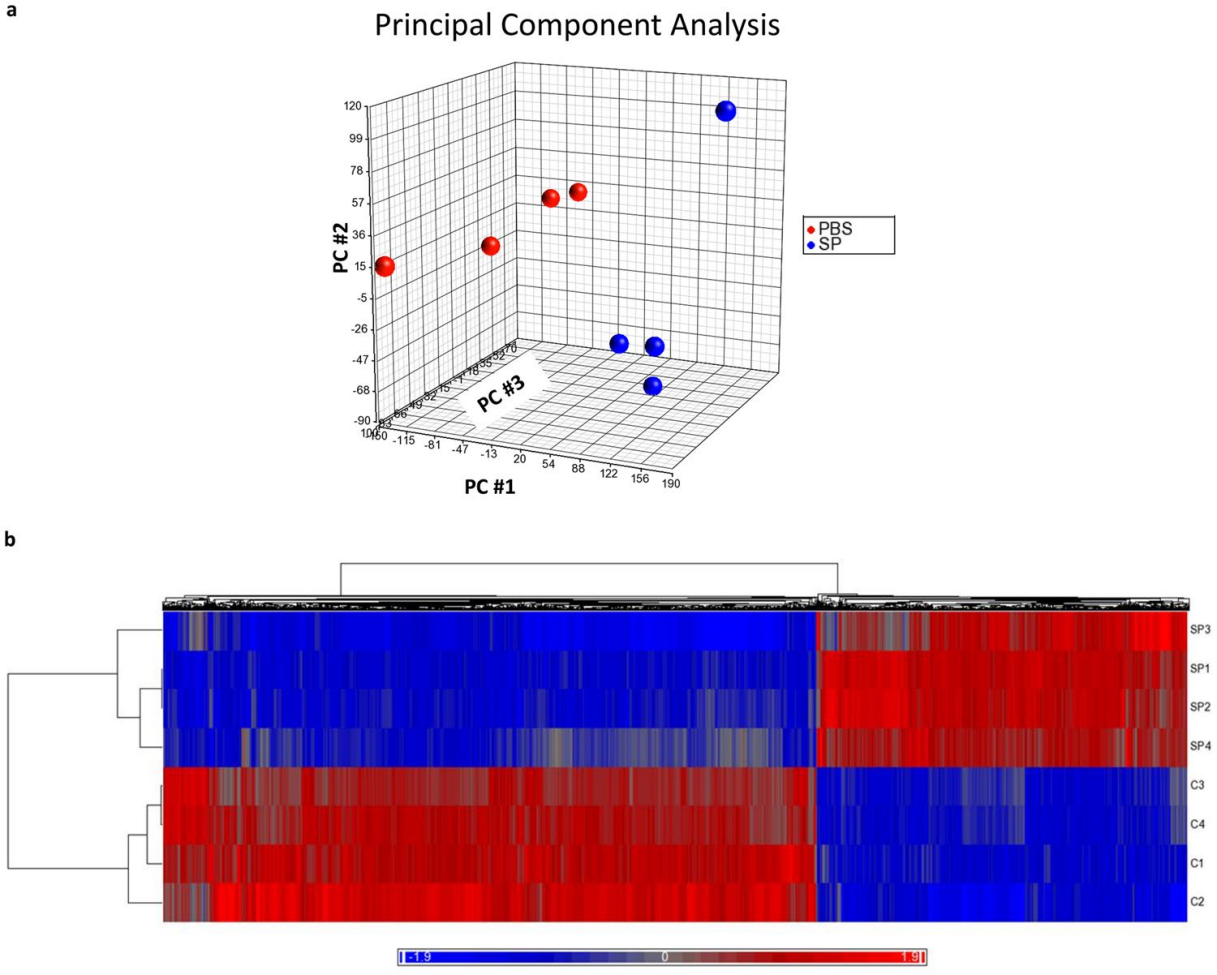
(**a**) Principal Component Analysis (PCA) of mRNA expression data showing the contributions of the top three principal components to the variance in mRNA expression by lung neutrophils from mice that received PBS compared to *S. pneumoniae* (n = 4 in each group). The top 3 principal components (PC) together account for 83.1% of the total variance, with the top 3 PCs each accounting for 57.4%, 16.1% and 9.64% of the total variance, respectively. (**b**) Unsupervised hierarchical clustering of DE mRNAs in lung neutrophils from mice that received PBS or *S. pneumoniae*. DE mRNAs were identified using filtering criteria as described in the text. The heat map depicts the standardized intensity values for 4127 DE mRNAs after normalization and standardization. Mean expression was set at zero, and expression levels scaled to one standard deviation (bright blue for low expression levels and bright red for high expression levels). Each row represents a sample from a mouse given PBS or *S. pneumoniae* (as labeled) and each column represents a DE mRNA. The dendrograms show the results of hierarchical clustering across samples (left) or across DE mRNAs (top).

*Microarray analysis of mRNA expression in lung neutrophils from mice that received either S. pneumoniae or PBS reveals that S. pneumoniae induces widespread changes in neutrophil gene expression*. Dimension reduction using Principal Component Analysis reveals that the top 3 principal components (PCs) account for 83% of the total variance, with the first component (PC#1) alone accounting for 57% of the variance and showing clear separation between the PBS- and *S. pneumoniae-*treated samples (Fig. 1a). Unsupervised hierarchical clustering from the whole array data or differentially expressed genes shows that samples of neutrophils from lungs with PBS and *S. pneumoniae* cluster into their respective groups, indicating that samples given the same stimulus (PBS or *S. pneumoniae*) have broadly similar patterns of expression (Fig. 1b).

The expression levels of 9,939 interrogated mRNAs were significantly different in lung neutrophils from mice with *S. pneumoniae* pneumonia compared with those from mice given PBS at an FDR adjusted p value (q-value) ≤ 0.05. Of these, 4,127 were changed by at least 2-fold and were designated as differentially expressed (DE) mRNAs. Of the DE transcripts, 1495 (36%) were upregulated at least 2-fold during *S. pneumoniae* pneumonia and 2632 (64%) were downregulated. Gene Set Enrichment Analysis (GSEA) using Gene Ontology (GO) molecular function terms indicated that genes involved in host defense and immune processes showed highly significant enrichment in samples from mice given *S. pneumoniae*, whereas metabolic and motor pathways were enriched in PBS (Table 1).

**Table 1:** Top 25 molecular pathways significantly enriched in lung neutrophils from mice given PBS or *S. pneumoniae*.

Gene sets enriched in *S. pneumoniae* samples	Gene sets enriched in PBS samples
(GO:0035456) response to interferon-beta	(GO:0042384) cilium assembly
(GO:0002237) response to molecule of bacterial origin	(GO:0044782) cilium organization
(GO:0032496) response to lipopolysaccharide	(GO:0060271) cilium morphogenesis
(GO:0071219) cellular response to molecule of bacterial origin	(GO:0001578) microtubule bundle formation
(GO:0071222) cellular response to lipopolysaccharide	(GO:0003341) cilium movement
(GO:0071216) cellular response to biotic stimulus	(GO:0035082) axoneme assembly
(GO:0035458) cellular response to interferon-beta	(GO:0010927) cellular component assembly involved in morphogenesis
(GO:0032655) regulation of interleukin-12 production	(GO:0030031) cell projection assembly
(GO:0034097) response to cytokine	(GO:0007018) microtubule-based movement
(GO:0001819) positive regulation of cytokine production	(GO:0007224) smoothened signaling pathway
(GO:0032615) interleukin-12 production	(GO:0007368) determination of left/right symmetry
(GO:0042832) defense response to protozoan	(GO:0070286) axonemal dynein complex assembly
(GO:0045088) regulation of innate immune response	(GO:0042073) intraciliary transport
(GO:0045087) innate immune response	(GO:0021532) neural tube patterning
(GO:0034341) response to interferon-gamma	(GO:0021904) dorsal/ventral neural tube patterning
(GO:0045089) positive regulation of innate immune response	(GO:0009799) specification of symmetry
(GO:0071345) cellular response to cytokine stimulus	(GO:0009855) determination of bilateral symmetry
(GO:0019882) antigen processing and presentation	(GO:0048858) cell projection morphogenesis
(GO:0031349) positive regulation of defense response	(GO:0008589) regulation of smoothened signaling pathway
(GO:0032649) regulation of interferon-gamma production	(GO:0003351) epithelial cilium movement
(GO:0002675) positive regulation of acute inflammatory response	(GO:0032990) cell part morphogenesis
(GO:0019221) cytokine-mediated signaling pathway	(GO:0021591) ventricular system development
(GO:0032609) interferon-gamma production	(GO:0007017) microtubule-based process
(GO:0009617) response to bacterium	(GO:0009062) fatty acid catabolic process
(GO:0001562) response to protozoan	(GO:0036158) outer dynein arm assembly

Gene Set Enrichment Analysis (GSEA) was done using gene sets from the Molecular Signatures Database (http://www.broadinstitute.org/gsea/index.jsp). FDR < 0.00005.

Although not listed among the top 25 gene sets shown in Table 1, gene sets pertaining to adaptive immunity were also significantly enriched during pneumonia (FDR < 1.70 × 10^−5^), including GO: 0072678 (T cell migration), GO: 0002824 (positive regulation of adaptive immune response based on somatic recombination of immune receptors built from immunoglobulin superfamily domains) and GO: 0002821 (positive regulation of adaptive immune response). These results indicate that neutrophils may play a role in shaping the subsequent adaptive immune response to *S. pneumoniae*, perhaps through effects on lymphocyte recruitment and activation. For example, expression of various chemokines that regulate lymphocyte chemotaxis are highly upregulated (*Ccl2*, *Ccl5*, *Cxcl9*, *Cxcl10*; see Supplementary Table 1) and the gene for the costimulatory receptor CD86 is upregulated 2.7 fold (FDR = 0.05).

*Neutrophils upregulate expression of inflammatory genes*. Among the gene sets that were identified as being highly significantly enriched during pneumonia (Table 1) were several large gene sets comprising numerous inflammatory genes (Supplementary Figure). Lung neutrophils from mice with pneumonia showed increased expression of genes that encode a number of mediators, including the important pro-inflammatory cytokines IL-1α, IL-6, TNF and CXCL1 (KC) (Supplementary Table 1). The ability of neutrophils to respond to inflammatory stimuli is enhanced during pneumonia, as evidenced by the increased expression of pattern recognition receptors and receptors for cytokines and other inflammatory mediators. Anti-inflammatory genes including *Il10* and *Il1rn* are also increased at this time (Supplementary Table 1). Upregulation of the *Cxcl1*, *Cxcl5*, *Cxcl9 Cxcl11*, *Tnf*, *Il1a*, *Il6* and *Il10* was confirmed in an independent cohort of lung neutrophil samples isolated from mice given PBS or *S. pneumoniae* using TaqMan assays and RT-qPCR (Supplementary Table 2). Increased expression of several DE genes that are important in the inflammatory and immune response were also validated at the protein level, namely the cytokines IFN-γ and TNF, the adhesion molecules ICAM-1, CD11c, CD11b and CD103, and the Fc receptor CD64 (Supplementary Table 3).

*Induction of type I interferon (IFN*) *response*. Induction of the type I IFN response in the lung during *S. pneumoniae* infection was apparent in the upregulation of type I IFN-responsive genes in neutrophils. The IFN-α/β receptor consists of two subunits, *Ifnar1* and *Ifnar2*, both of which are abundantly expressed in unstimulated neutrophils. During pneumonia, lung neutrophils upregulated expression of *Ifnar1* and *Ifnar2* mRNA 2.3 and 1.5 fold, respectively (FDR < 0.05). Furthermore, during pneumonia, lung neutrophils increased expression of a number of IFN-responsive genes, including the inflammatory mediators *Ccl2*, *Ccl5*, and *Cxcl10*, antiviral effector enzymes *Oas1*, *Oas2*, *Oas3*, and *Ddx60*, and transcription factors and signaling molecules involved in the interferon pathway such as *Irf1* and *Stat1* (Supplementary Table 1). However, normalized log2 expression levels were low for the genes encoding IFN-α isoforms and IFN-β, and these genes were not induced 2-fold or more during pneumonia. In an independent set of samples assayed using RT-qPCR, *Ifnar2* and the interferon-responsive genes *Cxcl11*, *Ifit1, Irf5* and *Isg15* were confirmed to be upregulated during pneumonia (Supplementary Table 2). Taken together, these studies suggest that neutrophils are well able to respond to type I IFNs through the IFN-α/β receptor to produce target genes, but do not appear to be a major source of Type I IFNs at this time point.

*Type II IFN (IFN-γ) production and activity*. Consistent with our previous report^4^, the pleiotropic cytokine IFN-γ was upregulated 2.3-fold in lung neutrophils from mice given *S. pneumoniae* for 24 h compared with PBS controls (FDR = 0.02). Notably, GSEA identified “cellular response to IFN-γ” and “production of IFN-γ” among the GO biological processes significantly upregulated during pneumonia (FDR < 10^−5^, FWER p value < 0.001 for both sets). Upregulated IFN-γ responsive genes included *Nos2, Irf1, Ccl2, Ccl5*, and *Gbp2-3* (Supplementary Table 1). Neutrophils also upregulated genes that encode proteins in the IFN-γ signaling pathway, including *Jak2* (3.3-fold) and *Stat1* (3.5-fold), and genes that encode cytokines inducing IFN-γ production, including *Il12a* (25-fold) and *Il18* (2.7-fold). Upregulation of the IFN-γ responsive genes *Nos2* and *Cxcl9* during pneumonia were confirmed by RT-qPCR in independent samples (Supplementary Table 2). Taken together, these results show that neutrophils simultaneously upregulate both production and sensing of Type II IFN, in contrast to type I IFN signaling for which sensing occurs but not production

*The role of NF-κB*. Numerous genes regulated by the transcription factor NF-κB were increased in neutrophils during *S. pneumoniae* pneumonia, including cytokines and chemokines, growth factors, receptors and intracellular signaling molecules (Supplementary Table 1). Transcription factors that are either regulated by NF-κB or regulate NF-κB activation were also differentially expressed (Supplementary Table 1). Upregulation of genes for pattern recognition receptors that signal upstream of NF-κB activation (for example, *Nod1, Nod2, Tlr2, Tlr4, Tlr6, Tlr7, Tlr9, Nlrp3*), cytoplasmic signaling molecules that signal downstream of these receptors (*Pycard, Casp1*), and mRNAs coding for proteins that interact with NF-κB subunits to regulate activation (*Nfkbia, Nfkbib, Nfkbie*) indicate complex regulation of and by NF-κB in neutrophils during *S. pneumoniae*-induced pneumonia. Changes in the nucleic acid sensors were variable in lung neutrophils. DAI and RIG-I family expression levels increased, but curiously, MAVS was downregulated.

There were interesting changes in multiple pathways leading to NF-κB activation in neutrophils (Supplementary Table 1). *S. pneumoniae* leads to strong induction of the IKK-related kinases *Ikbke* and *Tbk1* which we confirmed by RT-qPCR in an independent set of samples (Supplementary Table 2 *)*, whereas *Chuk* (IKKα) and *Ikbkb* (IKKβ) are not induced. While the role of IKKα and IKKβ in canonical and non-canonical NF-κB activation has been examined extensively, TBK1 has been shown to contribute to canonical and non-canonical NF-κB activation in some contexts while repressing NF-κB activation in others^18–24^. As noted in other tables, multiple transcriptional targets consistent with activation of TBK1 and IKKε were upregulated, for example, *Irf7*, *Cxcl10*, *Ifit1*, *Mx1*, *Mx2* and *Oas*-family members. Overall, these changes are consistent with increased TBK1 and IKKε kinase activity, as well as activation of Irf3/7 and the NF-κB pathways. Of note, many of these genes encode antiviral proteins (e.g., Mx1/2, IFITs, OAS) that to date have a less well-defined role in bacterial infections.

Both canonical and non-canonical IKKs require adaptor or scaffold proteins such as Ikbkg (NEMO), Tbk1bp1 (also called SINTBAD), Azi2 (also called Nap1) and TANK to direct them to specific subcellular locations and assist in substrate binding^21, 25, 26^. *S. pneumoniae* caused significant upregulation of genes encoding NEMO, SINTBAD, TANK, and mild upregulation of OPTN and Azi2, but no change in the expression of the genes encoding adaptors Calcoco2 (Ndp52), SQSTM1 (p62), RIOK, or DOK3. It is possible that selective expression of IKK adaptor proteins may direct the specificity of response in this infection model; namely, upregulation of NEMO, SINTBAD and TANK without upregulation of other scaffolds such as RIOK3 and DOK3 may facilitate high expression of NF-κB target genes (*Il1a*, *Tnf*, *Nos2*) and type I IFN responsive genes (*Ifit1/2/3*, *Oas1/2/3*, *Gbp3*) while limiting overall expression of IFNα and IFNβ themselves.

*The role of AP-1*. The transcription factor AP-1 is activated by cytokines, growth factors, inflammatory mediators, and other stimuli via MAPK signaling, and its function can be assessed by analysis of its target genes. Canonical AP-1 targets are upregulated in neutrophils at least 2-fold during *S. pneumoniae* pneumonia, indicating that AP-1 contributes to the neutrophil transcriptional response to *S. pneumoniae* in the lungs (Supplementary Table 1).

*The role of Nrf2 during pneumonia is evaluated by expression of its target genes*. The redox-sensitive transcription factor Nrf2 is highly expressed in lung neutrophils, and its mRNA expression does not change significantly during *S. pneumoniae* pneumonia (upregulated 1.3-fold, FDR = 0.09), consistent with post-translational regulation of its function^27, 28^. The expression levels of known Nrf2-regulated genes are increased at least 2-fold in lung neutrophils by *S. pneumoniae*, including the redox enzymes and the validated direct targets of Nrf2, *Sod2*, *Hmox1*, *Nqo1*, and *Txnrd1* (Supplementary Table 1). Several additional Nrf2-regulated genes were upregulated 1.5–1.9 fold (Supplementary Table 1). Upregulation of the Nrf2 target genes *Nqo1* and *Hmox1* were confirmed by RT-qPCR (Supplementary Table 2). However, other genes known to be positively regulated by Nrf2 are down-regulated during *S. pneumoniae* pneumonia, underlining the fact that the response to redox stress during pneumonia is multifactorial and complex (Supplementary Table 1). The number of differentially expressed Nrf2 regulated genes and the wide range of critical functions these genes perform indicate that the Nrf2-mediated response to oxidative stress is robust and likely to play an important role in regulating neutrophils during pneumonia^29^.

### Verification of mRNA profiling by RT-qPCR and flow cytometry

An independent cohort of lung neutrophil samples isolated from mice given PBS or *S. pneumoniae* was prepared, and TaqMan assays and RT-qPCR were used to confirm mRNAs and miRNAs identified in microarray profiling. These are discussed in depth in the sections above. In summary, upregulation of the mediators *Cxcl1*, *Cxcl5*, *Cxcl9 Cxcl11*, *Tnf*, *Il1a*, *Il6* and *Il10* was confirmed (Supplementary Table 2). Interestingly, in the interferon signaling pathways, *Ifnar2* and the interferon-responsive genes *Cxcl11*, *Ifit1*, *Irf5* and *Isg15* were verified to be upregulated during pneumonia (Supplementary Table 2), documenting that neutrophils are well able to respond to type I interferon signaling. Consistent with our previous studies regarding the role of IFNγ in neutrophils during *S. pneumoniae* pneumonia^30^, we confirmed upregulation of the IFN-γ responsive genes *Nos2* and *Cxcl9*. The Nrf2 target genes *Nqo1* and *Hmox1* were also verified by RT-qPCR (Supplementary Table 2).

Furthermore, increased expression of several DE genes that are important in the inflammatory and immune response were verified at the protein level, namely the cytokines IFN-γ and TNF, the adhesion molecules ICAM-1, CD11c, CD11b and CD103, and the Fc receptor CD64 (Supplementary Table 3). These molecules are discussed above in sections appropriate to their function.

### miR expression profiling

**Figure 2:**
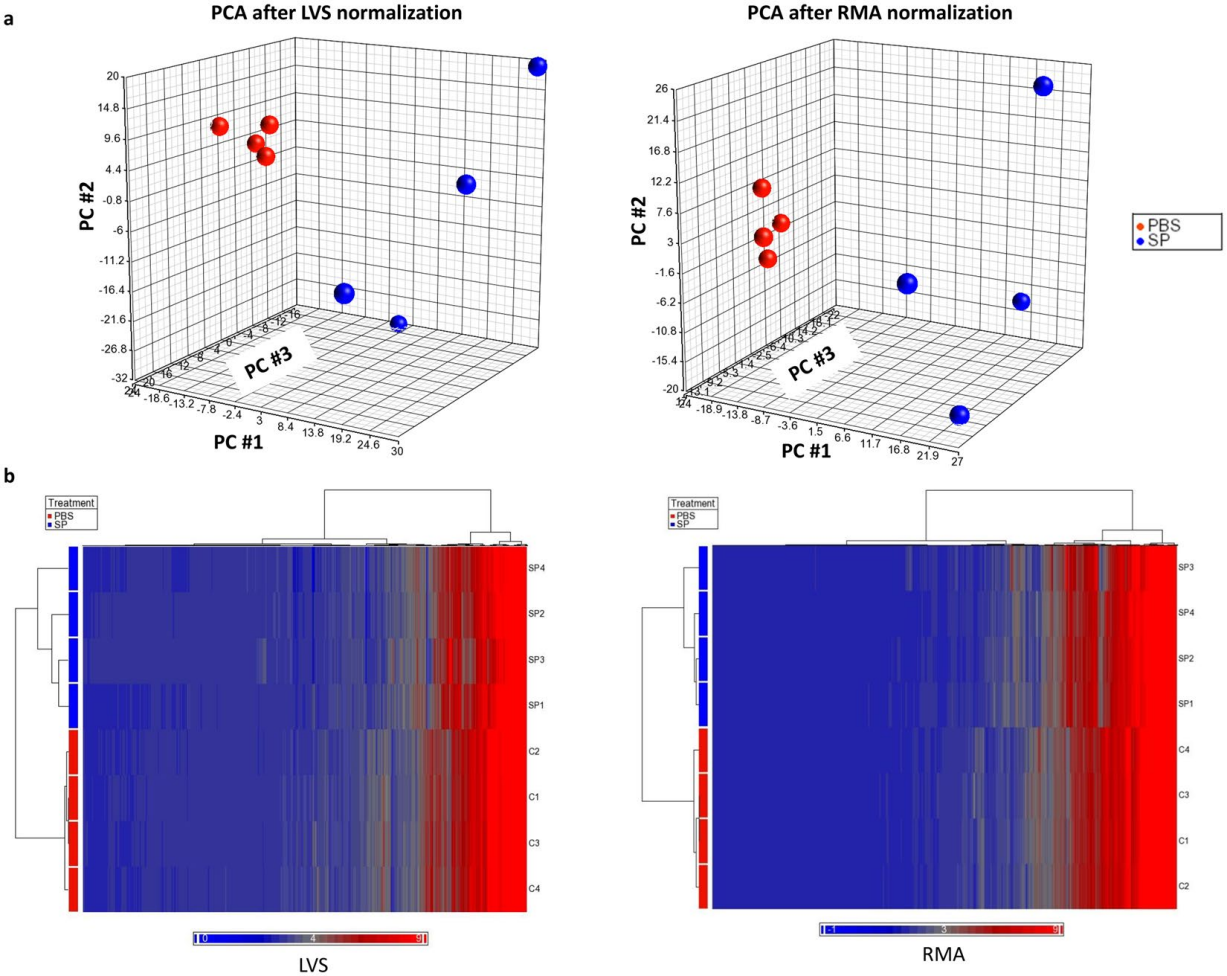
(**a**) PCA of miR profiling data after normalization using LVS (left) or RMA (right) shows the contributions of the top three principal components to the variance in miR expression by lung neutrophils from mice that received PBS or *S. pneumoniae*. Together, the top three PCs account for 76% of the total variance after normalization using LVS or 72% of total variance after normalization using RMA. Using LVS normalization, the top 3 principal components each account for 42.7%, 20.6% and 12.9% of the total variance, respectively. Using RMA normalization, the top 3 principal components each account for 43.5%, 16.1% and 12.3% of the total variance, respectively. (**b**) Unsupervised hierarchical clustering of all miRs in lung neutrophils from mice that received PBS or *S. pneumoniae*. The heat map depicts the standardized intensity values for miRs after normalization using LVS (left panel) or RMA (right panel) and standardization, with mean expression set at zero and expression levels scaled to one standard deviation (bright blue for lower expression levels and bright red for high expression levels). Each row represents a sample from a mouse given PBS or *S. pneumoniae* (as labeled) and each column represents a miR. The dendrograms show the results of hierarchical clustering across miRs (left side of each panel) or across samples (top of each panel).

**Figure 3:**
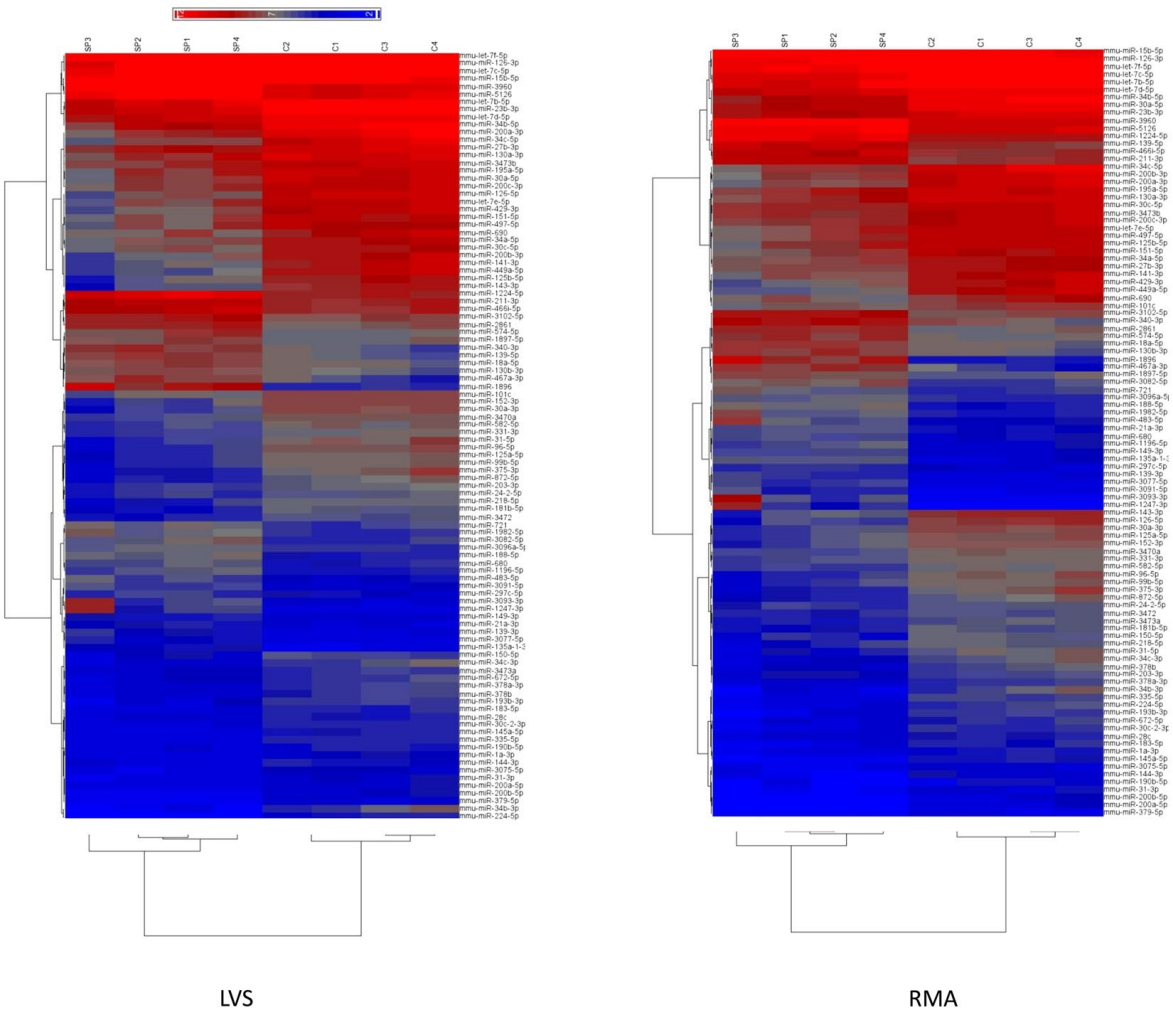
Unsupervised hierarchical clustering of DE miRs in lung neutrophils from mice that received PBS or *S. pneumoniae*. DE miRs were identified using filtering criteria as described in the text after normalization using LVS (left panel) or RMA (right panel). The heat map depicts the standardized intensity values for DE miRs after standardization.

*Microarray analysis reveals that S. pneumoniae induces widespread changes in neutrophil miR expression*. The expression of over 1100 mouse miRs was profiled in neutrophils isolated from the lungs of mice given PBS or *S. pneumoniae* intratracheally. The data were normalized using two different approaches: least-variant set (LVS) and robust multiarray average (RMA). PCA showed that top three PCs account for 76% or 72% of total variance using normalization by LVS or RMA, respectively. The first principal component (PC#1) accounted for approximately 43% of the variance using both methods and showed clear distinction between PBS and *S. pneumoniae*-treated samples. Neutrophils from mice with *S. pneumonia*e pneumonia grouped separately from the PBS-exposed neutrophils (Fig. 2a). Hierarchical clustering using all profiled miRs or DE miRs shows that samples cluster into their respective treatment groups (Figs 2b and 3).

Comparison of neutrophils from mice given *S. pneumoniae* with those from mice given PBS reveals that the expression of 374 or 312 miRs is significantly different (FDR ≤ 0.05) after normalizing the data using LVS or RMA, respectively. A list of 98 consensus DE miRs was generated from these by selecting miRs that were changed at least 2-fold in both RMA- and LVS-normalized data sets (Supplementary Table 4). Of the 98 DE miRs, 31 were upregulated and 67 were downregulated more than 2-fold in neutrophils from *S. pneumoniae* pneumonia compared with PBS controls. Three recently described miRs exhibited the highest fold-change (upregulation) using LVS or RMA normalization: mmu-miR-3093-3p on chromosome 3 (22.5 fold and 6.8-fold by RMA and LVS, respectively), mmu-miR-1896 on chromosome 13 (22-fold and 15-fold by RMA and LVS, respectively) and mmu-miR-1247-3p on chromosome 12 (21-fold and 8-fold by RMA and LVS, respectively). Of the downregulated miRs, mmu-miR-224-5p was down 7-fold and 31-fold by RMA and LVS, respectively. Mmu-miR-449a-5p was down 10-fold by both RMA and LVS. Three members of the mir-34 family were downregulated: mmu-miR-34b-3p was down 11.5-fold and 18-fold by RMA and LVS, respectively, whereas mmu-miR-34b-5p and mmu-miR-34c-5p show relatively high expression and 7 to 9-fold downregulation during pneumonia. miR-34 has been shown previously to be suppressed by NF-κB activation and is a known inhibitor of the inflammatory response^31^.

To validate the findings in the microarray expression profiling study, the expression of several miRs were measured using RT-qPCR in a different set of lung neutrophil samples generated in an independent study of mice given PBS or *S. pneumoniae* (Supplementary Table 5). Confirming the results of the gene profiling, mmu-miR-15b-5p and mmu-miR-223-3p were significantly upregulated during pneumonia, and mmu-miR-34b-5p and mmu-miR-126-3p were significantly downregulated. The four members of the let7 family that were tested tended to be downregulated, although the differences did not reach significance except for mmu-let-7f-5p (Supplementary Table 5). The other tested miRs were up or downregulated during pneumonia as found by gene expression profiling, except for mmu-miR-125b-5p, which was significantly down in the profiling study but was widely variable in the verification study.

*Some miRs are highly expressed in neutrophils from control mice with PBS instillation*. Consistent with data in the literature describing miR expression in neutrophils or granulocytes, miR-223 and miR-142 are highly expressed in neutrophils from un-infected lungs^13–15^. Of the 25 most highly expressed miRs in neutrophils from mice given PBS, 16 were identified as being significantly changed during pneumonia using LVS normalization and 17 using RMA, and the majority of these were downregulated (13 of 16 using LVS, 12 of 17 using RMA, Table 2). Two of the most highly expressed miRs are only recently described: mmu-miR-3963 (on chromosome 3)^32^, and mmu-miR-5100 (on chromosome 11)^33–36^. In fact, three of the genes identified by Chijiiwa and colleagues and Huang and colleagues as targets of mmu-miR-5100 are also significantly downregulated in neutrophils in pneumonia: *Podxl* (−3.67 fold), *Rab6b* (−2.37 fold), and *Rab6a* (−1.33 fold)^34, 36^, even though expression of this mmu-miR-5100 didn’t change.

**Table 2:** The 25 most highly expressed miRNAs in lung neutrophils from mice given PBS and the corresponding changes in expression during pneumonia.

Top 25 miRNAs expressed in PBS using LVS normalization (listed by expression value)	Fold change during pneumonia using LVS normalization	Top 25 miRNAs expressed in PBS using RMA normalization (listed by expression value)	Fold change during pneumonia using RMA normalization
mmu-miR-223-3p	2.1*	mmu-miR-223-3p	1.5
mmu-miR-21a-5p	1.3	mmu-miR-3963	−1.3*
mmu-miR-142-3p	1.7	mmu-miR-21a-5p	1.3*
mmu-let-7f-5p	−3.4*	mmu-miR-126-3p	−3.9*
mmu-let-7a-5p	−2.4*	mmu-miR-142-3p	1.6
mmu-miR-3963	−1.2	mmu-let-7f-5p	−2.4*
mmu-miR-126-3p	−4.2*	mmu-let-7a-5p	−1.8*
mmu-let-7c-5p	−2.6*	mmu-let-7c-5p	−2.6*
mmu-miR-26a-5p	−2.6*	mmu-miR-23a-3p	−1.5*
mmu-let-7b-5p	−3.2*	mmu-miR-26a-5p	−2.0*
mmu-miR-15a-5p	1.8*	mmu-let-7b-5p	−2.3*
mmu-miR-23a-3p	−2.1*	mmu-miR-29a-3p	1.2
mmu-miR-15b-5p	2.4*	mmu-miR-5100^c^	1.1
mmu-let-7g-5p	−1.1	mmu-miR-15b-5p	3.2*
mmu-miR-34b-5p	−8.0*	mmu-let-7g-5p	1.3*
mmu-miR-24-3p	−1.8*	mmu-miR-34b-5p	−6.7*
mmu-miR-29a-3p	−1.0	mmu-miR-16-5p	2.0*
mmu-miR-23b-3p	−2.8*	mmu-miR-15a-5p	2.3*
mmu-let-7i-5p	−1.6*	mmu-miR-24-3p	−1.5*
mmu-miR-19b-3p^a^	1.1	mmu-miR-26b-5p	1.1
mmu-miR-26b-5p	−1.1	mmu-let-7i-5p	−1.1
mmu-miR-16-5p	1.5*	mmu-miR-30a-5p^d^	−4.1*
mmu-miR-29c-3p	−1.1	mmu-let-7d-5p	−2.1*
mmu-let-7d-5p	−3.0*	mmu-miR-29c-3p	1.1
mmu-miR-107-3p^b^	−1.3	mmu-miR-29b-3p	−1.2

Data were normalized using LVS or RMA. Positive (negative) values indicate up (down) regulation of expression compared with PBS. *p ≤ 0.05 compared with PBS (FDR corrected). Notes on individual miRs; ^a^mmu-miR-19b-3p is ranked 30^th^ in RMA; ^b^mmu-miR-107-3p is ranked 41^st^ in RMA; ^c^mmu-miR-5100 was ranked 31^st^ in LVS; ^d^mmu-miR-30a-5p is 42^nd^ in LVS.

### mRNA and miR interactions and integration of their expression in a signaling network schema

*Conserved DE miRs and their anti-correlated predicted targets during pneumonia*. The data for conserved miRs are presented in Supplementary Table 6 and those for poorly conserved miRs are in Supplementary Table 7. A greater proportion of DE predicted target genes of DE miRs are down rather than upregulated during pneumonia (Supplementary Tables 6 and 7). The anti-correlated DE mRNAs that are predicted targets for the conserved DE miRs and that are up or downregulated during pneumonia are listed in Supplementary Tables 8 and 9, respectively. Targets for two conserved DE miRs are currently not in the TargetScanMouse 7.1 database: mmu-miR-126a-3p and mmu-miR-203-3p.

To gain further insight into the role of miRs in regulating target mRNA expression, our subsequent analysis focused largely on conserved miR families. Seven conserved DE miRs are upregulated during pneumonia and are expressed at moderate to high levels in lung neutrophils (average normalized mean expression above 5): mmu-miR-1224-5p, mmu-miR-188-5p, mmu-miR-139-5p, mmu-miR-15b-5p, mmu-miR-721, mmu-miR-18a-5p, and mmu-miR-130b-3p. Two of these, mmu-miR-721 and mmu-miR-130b-3p, belong to the same broadly conserved miR family and are therefore predicted to share target mRNAs in common. Our analysis identified 379 downregulated DE mRNAs that are predicted to be targets of these 7 upregulated conserved DE miRs, and 171 (45%) of these mRNAs are predicted targets of 2 or more of the DE miRs. Strikingly, 10 downregulated DE mRNAs are predicted targets of 4 or more of the upregulated DE miRs (*Dip2c*, *Dynll2*, *Efnb2*, *Enah*, *Lrp2*, *Nfib*, *Sos2*, *Trim2*, *Wdr50*, and *Zbtb20*), and several of these mRNAs act in processes that are likely to be critical in neutrophil function and host defense during infection, including gene transcription, cell migration and apoptosis (Supplementary Table 10).

There are 37 conserved DE miRs that are downregulated during pneumonia. These include 5 members of the broadly conserved let-7 family (mmu-let-7b-5p, mmu-let-7c-5p, mmu-let-7d-5p, mmu-let-7e-5p, and mmu-let-7f-5p); 2 members of the miR-30 family (mmu-miR-30a-5p and mmu-miR-30c-5p), and 3 members of the miR-34 family (mmu-miR-34a-5p, mmu-miR-34b-5p and mmu-miR-34c-5p).

**Figure 4:**
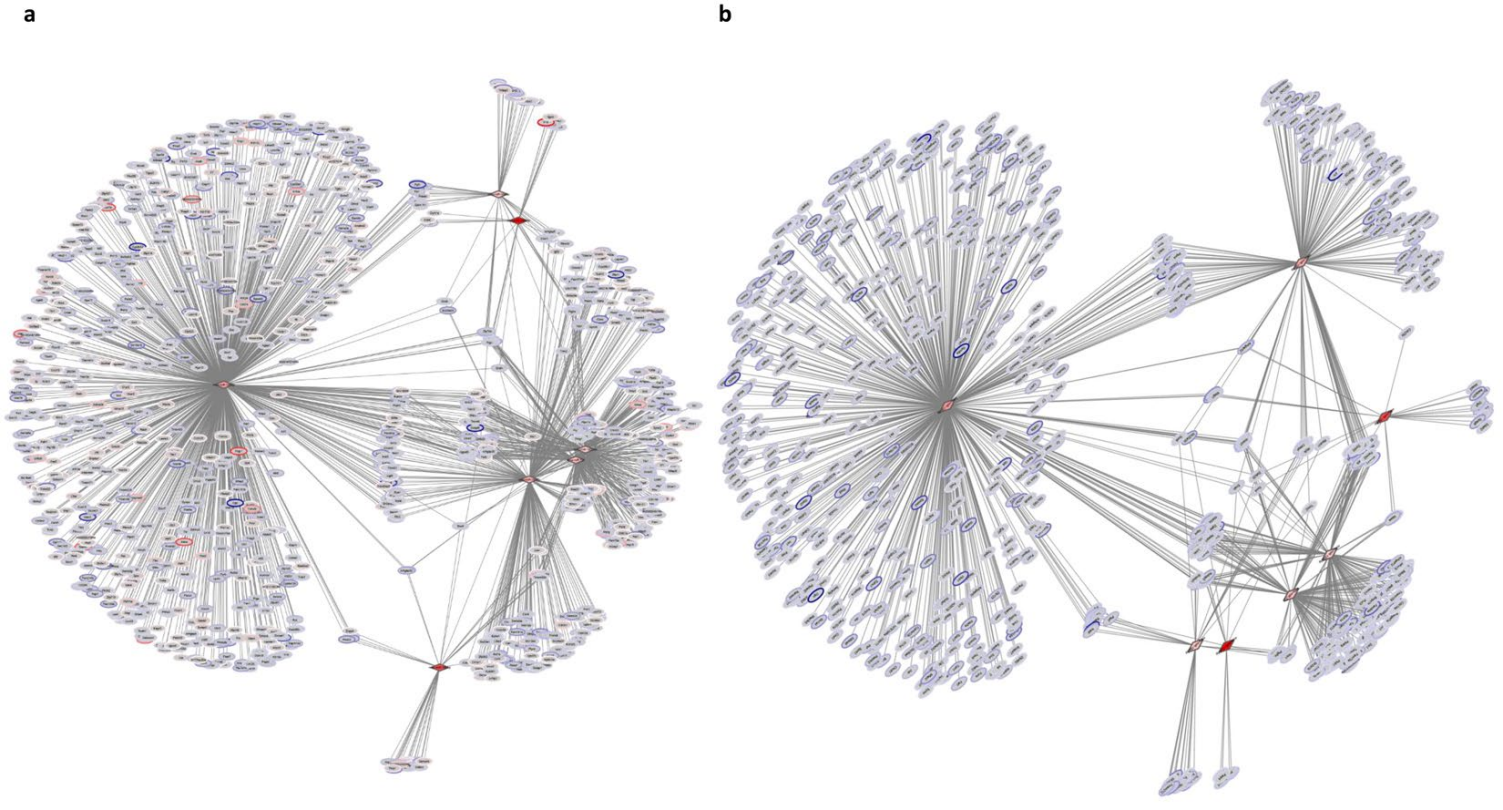
(**a**) Network describing the relationships of the 7 upregulated conserved miRs during pneumonia (red triangles) and their DE predicted mRNA targets (ovals, red if upregulated during pneumonia and purple if downregulated). (**b**) The expression of a subset of the DE mRNAs in panel a are anti-correlated with the targeting miR.

*Networks incorporating the changes in expression levels of conserved DE miRs and their DE predicted targets were constructed and visualized*. DE miRs were screened for those belonging to conserved miR families and showing moderate or greater expression levels. Lists of miR-target connections between these DE miRs and their target DE mRNAs were compiled from TargetScanMouse 7.1 target predictions. The network based on 7 upregulated DE miRs and their predicted target mRNAs that are also DE is shown in Fig. 4a for both correlated and anti-correlated DE mRNAs. This same network based on these 7 upregulated DE miRs but including only their anti-correlated predicted DE target mRNAs clearly shows that a substantial number of the predicted mRNA targets are shared by 2 or more candidate regulatory miRs (Fig. 4b).

**Figure 5:**
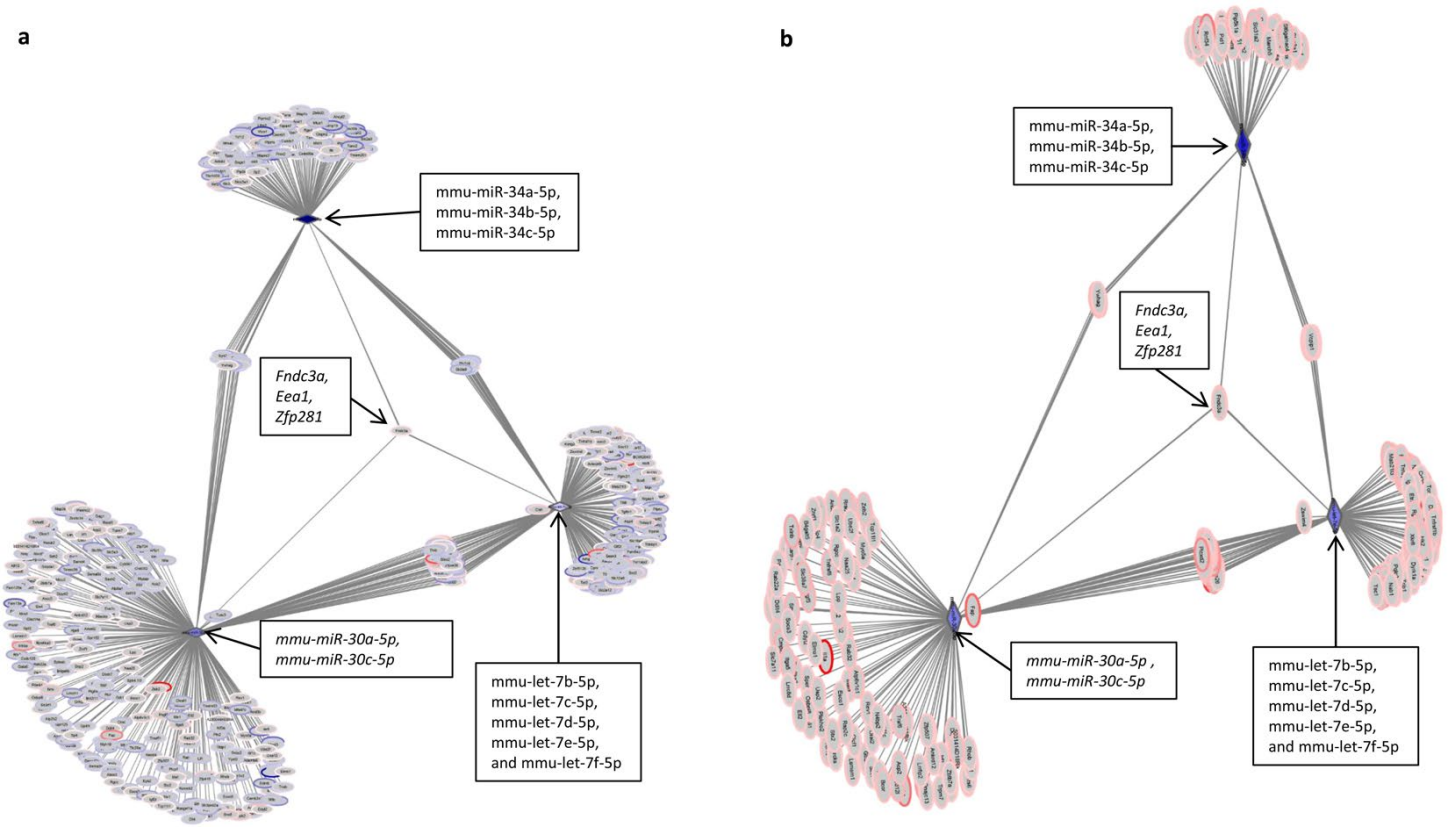
(**a**) Network showing downregulated miRs (purple triangles) belonging to 3 highly conserved miR families and their DE predicted mRNA targets (ovals). (**b**) The DE predicted targets from panel a that are anti-correlated with the miR. Strikingly, 3 DE mRNAs are predicted targets of all 3 miR families: *Eea1* (early endosome antigen 1), *Fndc3a* (fibronectin type III domain containing 3A) and *Zfp281* (zinc finger protein 281). Networks were visualized using Cytoscape. DE mRNAs that are upregulated during pneumonia are colored red, whereas downregulated DE mRNAs are colored purple. Gray lines connect DE miRs with their predicted target mRNAs.

A network containing all 37 conserved DE miRs that are downregulated during pneumonia is extremely complex and difficult to interpret. Instead, a more tractable network consisting of downregulated conserved miRs belonging to 3 miR families and their DE predicted targets was constructed (Fig. 5a). This network shows that most of the DE predicted targets were correlated, changing in the same direction as the miRs (Fig. 5a). However, there are 227 upregulated predicted target mRNAs, 41 (18%) of which are targeted by 2 or more of the 3 miR families (Fig. 5b). Strikingly, 3 DE mRNAs (*Eea1*, *Fndc3a* and *Zfp281)* are predicted targets of all 3 miR families. Pathway analysis of the over 200 upregulated DE target mRNAs indicated significant representation of many innate immune pathways, including apoptosis involving BCL-2 family members, JAK-STAT signaling, Notch signaling, SLC-mediated transport and Toll-like receptor cascades. Thus, these networks based on putative key regulatory miRs demonstrate the complexity of miR-mRNA interactions in neutrophils.

**Figure 6:**
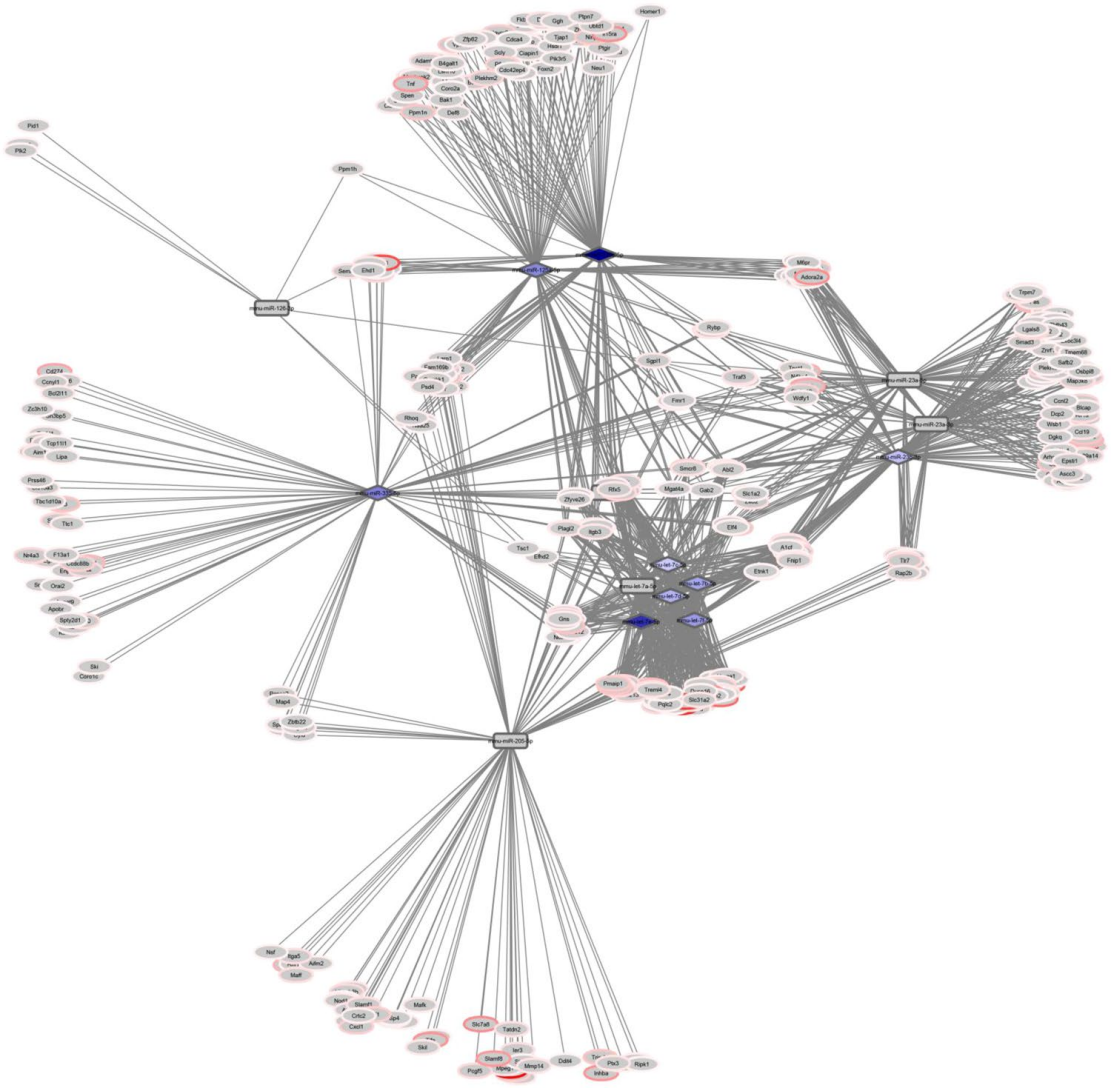
Network depicting downregulated miRs that have been identified as putative key regulatory miRs based on the differential expression of their predicted mRNA targets (miRhubs analysis), and their upregulated DE predicted targets. Consensus DE miRs that are predicted to regulate many DE mRNAs are depicted as purple diamonds. Mmu-miR-126-3p which is a consensus DE miR predicted to target 8 DE mRNAs is depicted as a gray oblong. Downregulated miRs that are identified as putative hubs but that are not DE by the filtering criteria (miR-mmu-let-7a-5p, mmu-miR-23a-5p, mmu-miR-23a-3p, mmu-miR-205-5p) are also depicted as gray oblongs. DE mRNAs that are upregulated during pneumonia are colored red. Gray lines connect miRs with their predicted target DE mRNAs.

*DE mRNAs predict miR regulatory hubs*. A complementary approach to identify potential miR regulatory hubs in lung neutrophils during host response to *S. pneumoniae* is the miRHub algorithm, which determines whether the predicted regulatory effect of any given miR on a set of DE mRNAs is significantly greater than expected by chance^37, 38^. Eleven miR families were identified as candidate regulatory hubs of the downregulated genes (Supplementary Table 11); however, five of these were not present on the array. Only one of the remaining six candidates was differentially expressed (mmu-miR-96-5p), and it was downregulated during pneumonia in these lung neutrophils. Twenty-four different miR families were identified as potential regulatory hubs of the upregulated mRNAs (Supplementary Table 11). Fourteen individual miRs belonging to six miR families (miR-125a-5p/125b-5p/351/670/4319, let-7, miR-126-3p, miR-205/205ab, miR-335/335-5p and miR-23abc/23b-3p) were downregulated during pneumonia (Supplementary Table 11). The 14 downregulated miRs were predicted to target hundreds of upregulated mRNAs. Some of these mRNAs were targeted uniquely by only one of the miRs, whereas others were targeted by two or more miRs (Fig. 6). Ten individual miRs out of the 14 miRs belonging to five of the six miR families (miR-125a-5p/125b-5p/351/670/4319, let-7, miR-126-3p, miR-335/335-5p and miR-23abc/23b-3p) were consensus DE miRs, i.e., they were significantly downregulated at least 2-fold during *S. pneumoniae* pneumonia using both LVS and RMA normalization (Fig. 6 and Supplementary Table 11). The consensus DE miRs whose expression is anti-correlated with their DE predicted targets (mmu-miR-125a-5p/mmu-miR-125b-5p, mmu-let-7b-5p/mmu-let-7c-5p/mmu-let-7d-5p/mmu-let-7e-5p/mmu-let-7f-5p, mmu-miR-126-3p, mmu-miR-335-5p and mmu-miR-23b-3p) are designated as candidate key regulatory miRs (miRhubs), which may represent major control points in the network-level neutrophil response to *S. pneumoniae*. These candidate miRhubs are predicted to target 357 upregulated DE mRNAs (Supplementary Table 12). Of the 357 DE mRNAs, 122 (34%) are shared predicted targets of 2 or more of the candidate regulatory miRs (Supplementary Table 13).

## Discussion

The present study examined the response of neutrophils in the lungs during pneumonia induced by *S. pneumoniae*. Our previous studies have shown that greater than 98% of these neutrophils migrate out of the pulmonary microvasculature and into the lung tissue or airspace at 24 h after instillation of these bacteria^4^. Gene profiling shows that neutrophils alter their transcriptional profile massively during their response to *S. pneumoniae*. Pathway analysis indicates neutrophils display evidence of activation of immune and host defense pathways (Table 1), particularly the upregulation of expression of cytokines, chemokines, cytokine receptors, and pattern recognition receptors and adaptor molecules (Supplementary Table 1). The upregulation of many cytokine receptors and downstream signaling molecules suggests that neutrophil-derived mediators can act through autocrine or paracrine signaling to amplify the neutrophil response. Gene and pathway analysis also show that neutrophils have the potential to shape the subsequent adaptive immune response to *S. pneumoniae*, in part through effects on lymphocyte recruitment and activation. These results support the increasingly appreciated role of neutrophils as sources of cytokines and other mediators and as central players in cellular networks involving other immune cells^39^.

Because of the large number of lung neutrophil mRNAs and miRs that were changed during *S. pneumoniae* pneumonia, evidence of transcription factor activation was pursued by asking whether target genes of particular transcription factors were upregulated in these neutrophils. The data suggest that robust activation of a number of transcription factors in neutrophils occurs in response to *S. pneumoniae* in the lungs, including AP-1, Nrf2 and NF-κB, as assessed by expression of the genes they regulate (Supplementary Table 1). Direct demonstration of NF-kB activation in neutrophils using classical biochemical methods has been challenging, likely due to high protease activity in neutrophils^40^. Nrf2 is a redox-sensitive transcription factor encoded by *Nfe2l2* that regulates the transcription of antioxidants and other cytoprotective genes^41, 42^. Because neutrophils can produce large amounts of ROS rapidly, Nrf2 appears to play an important role in regulating neutrophil function and survival^29^. The cytokines regulated by these transcription factors, in turn, regulate production of mediators such as the chemokines KC and MIP-2.

The signaling pathways that lead to activation of these transcription factors likely include the recognition of bacteria or bacterial components by pattern recognition receptors and the binding of inflammatory mediators to receptors expressed on the neutrophil surface. These transcription factors then target inflammatory mediators, immune modulators, pattern recognition receptors and adaptor molecules, and other transcription factors. Transcription factors also regulate the expression of miRs, which often silence expression of their target genes. Thus, a complex and dynamic web of interactions involving transcription factors and miRs regulates the transcriptional response of neutrophils to bacteria in the lungs. Neutrophils are fully capable of responding to pathogens and producing the downstream mediators that recruit more neutrophils and modulate host defense in numerous ways, including modulation of resolution and initiation of innate immunity^30^.

This study identified a sizable proportion of neutrophil miRs that are differentially regulated during pneumonia induced by *S. pneumoniae* and may play critical roles in the host response. miRs were identified whose expression showed greater than 2-fold change during pneumonia compared with PBS, either upregulated during pneumonia (31 miRs) or downregulated (67 miRs). The observed fold changes in DE miRs range from 2-22, with the majority of the 98 consensus DE miRs changing less than five-fold (80% and 70% of the DE miRs by LVS and RMA, respectively). Importantly, miRs often target overlapping and/or functionally related target genes^43^, so miRs can play a major role in regulating important processes despite modest changes in their expression. Target mRNA suppression by microRNAs is affected not only by miR abundance, but also by target site accessibility, binding affinity and the ratio of the miR to its target^44–46^. The majority of the DE mRNAs that were identified as putative targets of the upregulated DE miRs showed decreased expression during pneumonia compared with PBS (for example, Fig. 4a compared to Fig. 4b). This finding is consistent with the concept that miRs negatively regulate expression of their target genes by decreasing mRNA levels, rather than by inhibiting translation^47^. These data support the idea that direct control of targets by upregulated miRs is a prominent regulatory feature of the neutrophilic response to *S. pneumoniae*.

To understand integrated regulatory interactions of miRs and mRNAs, candidate regulatory miRs were identified by constructing networks consisting of DE conserved miRs and their DE predicted targets (Figs 4 and 5). These networks demonstrated that potential target DE mRNAs may change in the same or the opposite direction as the DE miRs. Both also showed that an mRNA is often predicted to be targeted by more than one DE miR. We identified a network containing seven upregulated conserved miRs (mmu-miR-1224-5p, mmu-miR-188-5p, mmu-miR-139-5p, mmu-miR-15b-5p, mmu-miR-721, mmu-miR-18a-5p and mmu-miR-130b-3p) and another network consisting of downregulated miRs belonging to 3 highly conserved miR families (let-7, mir-30 and mir-34).

A complementary approach using miRHub analysis^37, 38^ identified candidate key regulatory miRs based on conserved target sites in DE mRNAs, of which 10 individual miRs are differentially expressed (mmu-miR-125a-5p/mmu-miR-125b-5p, mmu-let-7a-5p/ mmu-let-7c-5p/mmu-let-7d-5p/mmu-let-7e-5p/mmu-let-7f-5p, mmu-miR-126-3p, mmu-miR-335-5p and mmu-miR-23b-3p; see Fig. 6 and Supplementary Tables 8 and 9). Thus, each of these approaches identified different candidate key regulatory miRs for the regulation of mRNAs during *S. pneumoniae* pneumonia. Each approach uses a rigorously defined method based upon the most up-to-date available computational and biological information. Testing of each potential regulatory miR using *in vitro* and *in vivo* approaches will be needed to understand these complex systems.

Many of these candidate key regulatory miRs have been identified only recently, and their functions have not been studied. Others have been previously identified as prominent regulators of inflammatory pathways, including miR-34^31^,miR-3960 and miR-2861^48^, miR-126^49–51^ and let-7f^52^.

These studies assume that neutrophils in bulk reflect individual cell behavior. Whether there are truly distinct subtypes of neutrophils is unlikely. Differences between neutrophils more likely represent their plasticity, similar to that within subpopulations of macrophages, or changes that are part of the aging process in this very short-lived cell. No surface markers are known that can be used to discriminate subtypes of neutrophils having different functions; thus, cell sorting to study these functions is not feasible. The technology needed for single cell sequencing is not yet sufficient to allow studies of transcription within a single neutrophil. This approach will clearly be helpful as the technology improves.

In summary, neutrophils are highly responsive to *S. pneumoniae* by transcribing miRs and mRNAs. This dataset of DE miRs and DE mRNAs derived from lung neutrophils was used to identify particular transcription factors and cytokines that regulate the inflammatory response and the transition to innate immunity or resolution of inflammation. Key pathways were identified, and networks of DE miR-target mRNA interactions were built based primarily on computationally derived potential interactions derived from sequence information. These networks identified candidate key regulatory miRs. Neutrophils have a short lifespan and cannot be cultured; as such, knockdown technologies cannot be effectively employed, making further study of these miR-mRNA interactions difficult at the present time. Studies examining mice with conditional deletion of key regulatory miRs may prove very exciting. As our biological knowledge of these interactions grows, these networks will become even more useful in generating exciting novel testable hypotheses.

## Methods

### Reagents

Collagenase/ Dispase and Dispase II were purchased from Roche Applied Science (Indianapolis, IN); fetal calf serum (FCS) and phosphate buffered saline (PBS) were from Invitrogen (Carlsbad, CA); bovine serum albumin (BSA), deoxyribonuclease I from bovine pancreas and Red Blood Cell Lysing Buffer were from Sigma-Aldrich (St. Louis, MO). MACs columns, separators, and anti-Ly6G microbead kit were from Miltenyi (Auburn, CA). Hema3 fixative and staining solutions were obtained from Fisher Scientific (Kalamazoo, MI, USA).

#### Mice

Adult female C57BL/6 J mice were purchased from Jackson Laboratory (Bar Harbor, ME), and colonies were generated. Mice were housed in a specific pathogen-free facility and housed in sterile cages within ventilated racks. They received irradiated standard chow and water. Age- and sex-matched mice were studied at 6–8 weeks of age. All studies were subject to review by the Institutional Animal Care and Use Committee at the University of North Carolina at Chapel Hill and conformed to the Guide for the Care and Use of Laboratory Animals by the Institute of Laboratory Animal Resources, Commission on Life Sciences, National Research Council.

#### Bacterial pneumonia

*Streptococcus pneumoniae* (*S. pneumoniae*; *serotype* 19, ATCC 49619) was purchased from American Type Culture Collection (Manassas, VA). Suspensions of *S. pneumoniae* were prepared in PBS, and the target bacterial dose was estimated based on absorbance of the bacterial suspension at 600 nm (O.D. = 0.9). Pneumonia was induced by intratracheal instillation of the bacterial suspension into the left lung at a dose of 2.3 µl/g mouse body weight^29, 53^. Colony forming units (CFU) in bacterial suspensions were subsequently determined by plating serial dilutions of the bacterial suspension on agar plates. The range of CFUs was 1.71–1.88 × 10^**7**^ CFU/mouse (mean 1.8 × 10^7^ ± 3.8 × 10^5^ CFU/mouse). Control mice received an equal volume of PBS, in which the bacteria were resuspended.

#### Isolation of single lung cells

Mice were euthanized by isoflurane overdose 24 hours after bacterial instillation. The lung vasculature was flushed by perfusing with 10 ml of PBS via the right ventricle, and the lungs and heart were removed. Single cell suspensions of lung cells were prepared as described previously^4^. Dispase II solution was instilled into the lungs through the trachea, and the trachea was ligated with silk suture. The samples were incubated for 30 minutes at 37 °C. The lungs were excised, minced with scissors and enzymatically digested with 0.1% collagenase-dispase and 0.01% deoxyribonuclease I in PBS containing 5 mM CaCl_2_ at 37 °C for 10 min. The cells were passed through a 100 µm mesh to remove clumps. The cell suspension was resuspended in red blood cell lysis solution, washed several times with PBS and passed through a 40 µm mesh. The total number of cells in each sample was determined using a hemocytometer. Cell differential counts were performed by examining cytospins stained with Hema3.

#### Isolation of neutrophils from single cell suspensions of mouse lungs

Neutrophils were isolated from lung digests using Miltenyi’s anti-Ly6G microbead kit according to the manufacturer’s instructions. Briefly, single cell suspensions were prepared from dissected mouse lungs as described above. The cells were washed with PBS containing 2 mM EDTA and 0.5% BSA, and incubated with biotinylated anti-Ly6G and magnetic beads coated with anti-biotin. The samples were passed through a column in a magnetic field, and cells bound to beads were collected for further analysis. Cells were kept on ice or at 4–8 °C during processing and isolation. The purity of neutrophil isolation was evaluated in the samples from mice with pneumonia, in which the purity was greater than 97% as determined by examination of stained cytospin preparations. The few contaminating cells usually had the appearance of alveolar macrophages and rarely eosinophils. The neutrophil marker Ly6G mRNA was highly expressed in every sample.

#### RNA isolation

Total RNA was extracted from neutrophils using miRNeasy® Mini Kit from Qiagen (Valencia. CA).

#### Microarrays

mRNA expression was profiled using Mouse Gene 1.1 ST arrays (Affymetrix, Santa Clara, CA) which cover over 26,000 RefSeq transcripts. miRs were profiled using SurePrint G3 Mouse miRNA 8 × 60 K Microarray kits (Agilent, Santa Clara, CA) designed based on miRbase release 17. This approach to profiling mRNAs and miRs was selected after a comprehensive evaluation of platforms. Microarrays, RT-qPCR and sequencing each has strengths and weaknesses that make each appropriate for specific study goals^54^. Whereas sequencing offers advantages over microarrays or RT-qPCR in that the information extracted with sequencing can be more complex, the study by Mestdagh and colleagues showed Agilent arrays performed well compared with other hybridization technologies and were best at capturing small expression differences compared with all other methods^54^. The amount of RNA required for high quality next generation sequencing was greater than the RNA we could obtain from lung neutrophils isolated from a single mouse with pneumonia. Studies in the literature show that microarrays fared well compared with other methods and was indeed superior at detecting small differences in expression of miRs^54^.

#### Real Time-quantitative PCR

Expression of selected mRNAs and miRs was measured in independent samples generated from a separate study usingTaqMan mRNA gene expression assays (Thermo Fisher) and Exiqon miRcury LNA miRNA assays (Exiqon, Woburn, MA, USA) respectively, according to the manufacturers’ instructions. RT-qPCR was performed using the Applied Biosystems 7500 Real-Time System or QuantStudio 6 Flex Real-Time PCR System (Thermo Fisher Scientific). Fold changes were calculated using the method described by Livak and Schmittgen^55^.

### Flow cytometry

Single cell suspensions were prepared from dissected mouse lungs and stained using fluorochrome-conjugated antibodies^56^ that recognize cell surface markers and the cytokines TNF and IFN-γ present intracellularly. Antibodies used were purchased from BioLegend (San Diego, CA, USA) unless indicated otherwise: anti-mouse Ly6G (clone IA8), anti-mouse CD45 (clone 30-F11, BD Biosciences, San Jose, CA, USA), anti-mouse CD11b (clone M1/70), anti-mouse CD11c (clone N418), anti-mouse Ly6C (clone AL-21 BD Biosciences), anti-mouse MHC Class II I-A/I-E (clone M5/114.15.2), anti-mouse Siglec-F (clone E50-2440, BD Biosciences), anti-mouse CD54 (clone 3E2), anti-mouse CD103 (clone M290), anti-mouse IFNγ (XMG1.2), anti-mouse TNF (MP6-XT22), anti-mouse CD64 (clone × 54–5/7.1), and anti-mouse CD24 (clone M1/69). After gating out debris and doublet cells, neutrophils were identified as single cells expressing both CD45 and Ly6G. Flow cytometry was performed using a Cytoflex flow cytometer (Beckman Coulter, Brea, CA, USA) and data were analyzed using CytExpert (Beckman Coulter) software.

#### Microarray data analysis and statistics

Expression signals from Affymetrix mRNA arrays were preprocessed and normalized by RMA (Robust Multiarray Average) background correction, GC content and sequence correction, quantile normalization, and median polish summarization of probe signals mapped to specific genes. Custom probeset-to-gene mappings were generated from Affymetrix Probeset and Transcript Annotation release 35 by consolidating all probesets mapped, in order of preference, to Ensembl 81 gene ID, Refseq mRNA, and Genbank accession numbers. miR expression signals were extracted from microarray images using Agilent Feature Extraction (FE) software, and normalized using either LVSmiRNA or AgiMicroRna package from Bioconductor. These normalization methods were used based on literature suggesting distinct advantages offered by each in normalizing gene expression data^57–60^. LVSmiRNA uses the least-variant set of miRNA to normalize between the arrays^57^, while AgiMicroRna uses RMA.

The normalized log2 transformed intensities were analyzed for differential expression between treatment and control groups by one-way ANOVA. Differentially expressed (DE) miRs or mRNAs were filtered at Benjamini-Hochberg FDR adjusted p value or q-value < 0.05, and fold change >2. Fold change, expression level and p value were used as filtering criteria to identify possible candidate key miRs. These criteria were implemented based on a comprehensive study by the SEQC/MAQC-III consortium showing that relative expression measurements agreed fairly well across different expression profiling platforms and study sites after filtering for fold change, p value and expression level^61^. For miRs, the analyses were carried out on both LVS and RMA normalized log2 intensities in parallel. The consensus DE miRs between LVS and RMA were used in subsequent network analysis. Expression analyses were performed using Partek Genomics Suites v6.5 (Partek, Inc., St. Louis, MO). To identify pathways or functions that are significantly altered during *S. pneumoniae* pneumonia, Gene Set Enrichment Analysis (GSEA) using Gene Ontology (GO) biological process terms from mRNA expression data was performed according to the method described by Subramanian, Tamayo and colleagues^62, 63^. Gene ontology, biological functions and pathways and transcription factor binding sites were analyzed using DAVID Bioinformatics Resources^64, 65^ (NIAID, NIH) and InnateDB^66^.

To identify potential functionally significant miR-mRNA interactions, lists of target genes (mRNAs) were constructed for each DE miR based on predicted targets in the 3′-UTR of all genes using TargetScanMouse release 7.1 (TS7) as source for miR targets^67^. TargetScan predictions are based on the identification of 6-, 7- or 8-mer sequences in the 3′UTR of target mRNAs that are complementary to the seed region of miRs. miRs are grouped into families that share a common seed sequence, suggesting similar or closely related targets and functions. TargetScan classifies miR families as broadly conserved (expressed across most vertebrates, usually to zebrafish), conserved (expressed across most mammals, but usually not beyond placental mammals) or poorly conserved (comprising all remaining miRs).

Networks of miR-target connections between DE miRs and DE mRNAs were compiled from TS7 target predictions and miR family information downloaded from miRBase21. The networks were visualized using Cytoscape software^68^.

Candidate miR regulatory hubs were identified by miRHub^37, 38^, using the “non-network” mode and requiring a predicted target site to be conserved among at least three mammalian species including mouse. This algorithm determines whether the predicted regulatory effect of any given miR on a set of DE genes is significantly greater than expected by chance.

Online Supplementary Material is included.

#### *Availability of materials and data*

Microarray data have been deposited in Gene Expression Omnibus under accession number GSE97922 (http://www.ncbi.nlm.nih.gov/geo/).

## Electronic supplementary material

Supplementary tables and figure
