# Supplementary material for: Predicted effects of observed changes in the mRNA and microRNA transcriptome of lung neutrophils during S. pneumoniae pneumonia in mice
Source: Sci Rep. 2017 Sep 12;7:11258. doi: 10.1038/s41598-017-11638-7 (PMC5595893; doi:10.1038/s41598-017-11638-7)

**Supplementary Information**

Predicted effects of observed changes in the mRNA and microRNA transcriptome of lung neutrophils during *S. pneumoniae* pneumonia in mice

John C. Gomez^1,2,3^, Hong Dang^2^, Matthew Kanke^4^, Robert S. Hagan^1,3,5^, Jason R. Mock^1,3,5^, Samir N. P. Kelada^4^, Praveen Sethupathy^4^, and Claire M. Doerschuk^1,2,3,5, *^

^1^Center for Airways Disease, ^2^Cystic Fibrosis/Pulmonary Research and Treatment Center, ^3^Marsico Lung Institute, ^4^Department of Genetics,^5^Department of Medicine, University of North Carolina at Chapel Hill, Chapel Hill, NC, USA

**Supplementary Tables**

Supplementary Table 1: Fold changes in expression of selected genes in lung neutrophils during *S. pneumoniae* pneumonia

Supplementary Table 2: Expression of mRNA genes in lung neutrophils measured using RT-PCR

Supplementary Table 3: Protein expression of selected markers and cytokines in lung neutrophils

Supplementary Table 4. 98 consensus DE miRs.

Supplementary table 5: Expression of selected miRs in lung neutrophils measured using RT-qPCR

Supplementary Table 6: Conserved DE and the numbers and percentages of predicted mRNA targets that are differentially expressed during pneumonia

Supplementary Table 7: Poorly conserved DE miRs and the numbers and percentages of predicted mRNA targets that are differentially expressed during pneumonia

Supplementary Table 8: Conserved DE miRs that were upregulated during pneumonia and their anti-correlated predicted targets (mRNAs 2 fold or greater downregulated during pneumonia compared with PBS, FDR≤0.05).

Supplementary Table 9: Conserved downregulated DE miRs and their anti-correlated predicted targets

Supplementary Table 10: Downregulated DE mRNAs that are predicted targets of 4 or more upregulated conserved DE miRs

Supplementary Table 11: Candidate regulatory miRs based on miRhub analysis.

Supplementary Table 12: DE predicted targets of putative key regulatory miRs (in bold type) based on miRhubs analysis.

Supplementary Table 13. miR families and individual miRs identified as putative miRhubs and their unique and shared DE predicted targets.

**Supplementary Figure**

**Supplementary Figure:** Enrichment plots of large gene sets in the GO database that are significantly enriched during pneumonia according to GSEA.

**Supplementary Table 1:** Fold changes in expression of selected genes in lung neutrophils during *S. pneumoniae* pneumonia

| **Category** | **Gene** | **Gene Description** | **Fold change compared to PBS** |
| --- | --- | --- | --- |
| **Chemokines and chemokine receptors** |  |  |  |
|  | *Ccl12* | chemokine (C-C motif) ligand 12 | 7.2 |
|  | *Ccl17* | chemokine (C-C motif) ligand 17 | 3.2 |
|  | *Ccl19* | chemokine (C-C motif) ligand 19 | 4.6 |
|  | *Ccl2* | chemokine (C-C motif) ligand 2 | 16.4 |
|  | *Ccl20* | chemokine (C-C motif) ligand 20 | 5.8 |
|  | *Ccl27a* | chemokine (C-C motif) ligand 27A | -2.0 |
|  | *Ccl3* | chemokine (C-C motif) ligand 3 | 5.1 |
|  | *Ccl4* | chemokine (C-C motif) ligand 4 | 23.5 |
|  | *Ccl5* | chemokine (C-C motif) ligand 5 | 12.4 |
|  | *Ccl7* | chemokine (C-C motif) ligand 7 | 9.0 |
|  | *Ccl9* | chemokine (C-C motif) ligand 9 | 2.2 |
|  | *Ccr5* | chemokine (C-C motif) receptor 5 | 5.1 |
|  | *Cxcl1* | chemokine (C-X-C motif) ligand 1 | 7.7 |
|  | *Cxcl10* | chemokine (C-X-C motif) ligand 10 | 26.8 |
|  | *Cxcl11* | chemokine (C-X-C motif) ligand 11 | 47.0 |
|  | *Cxcl13* | chemokine (C-X-C motif) ligand 13 | 3.6 |
|  | *Cxcl15* | chemokine (C-X-C motif) ligand 15 | -3.2 |
|  | *Cxcl16* | chemokine (C-X-C motif) ligand 16 | 2.1 |
|  | *Cxcl17* | chemokine (C-X-C motif) ligand 17 | -4.9 |
|  | *Cxcl3* | chemokine (C-X-C motif) ligand 3 | 11.6 |
|  | *Cxcl5* | chemokine (C-X-C motif) ligand 5 | 4.3 |
|  | *Cxcl9* | chemokine (C-X-C motif) ligand 9 | 52.9 |
|  | *Cxcr3* | chemokine (C-X-C motif) receptor 3 | 2.1 |
| **Cytokines and receptors** |  |  |  |
|  | *Ifng* | interferon gamma | 2.3 |
|  | *Il10* | interleukin 10 | 23.2 |
|  | *Il10rb* | interleukin 10 receptor, beta | 2.6 |
|  | *Il12a* | interleukin 12a | 25.2 |
|  | *Il12rb1* | interleukin 12 receptor, beta 1 | 4.6 |
|  | *Il15ra* | interleukin 15 receptor, alpha chain | 18.0 |
|  | *Il17rc* | interleukin 17 receptor C | -2.6 |
|  | *Il17rd* | interleukin 17 receptor D | -2.1 |
|  | *Il17re* | interleukin 17 receptor E | -3.0 |
|  | *Il18* | interleukin 18 | 2.7 |
|  | *Il18bp* | interleukin 18 binding protein | 6.6 |
|  | *Il18r1* | interleukin 18 receptor 1 | -3.2 |
|  | *Il1a* | interleukin 1 alpha | 35.3 |
|  | *Il1bos* | interleukin 1 beta, opposite strand | 8.5 |
|  | *Il1f6* | interleukin 1 family, member 6 | 21.5 |
|  | *Il1f9* | interleukin 1 family, member 9 | 2.1 |
|  | *Il1rn* | interleukin 1 receptor antagonist | 6.2 |
|  | *Il20rb* | interleukin 20 receptor beta | 2.6 |
|  | *Il20rb* | interleukin 20 receptor beta | 2.5 |
|  | *Il21r* | interleukin 21 receptor | 2.8 |
|  | *Il22ra1* | interleukin 22 receptor, alpha 1 | -2.7 |
|  | *Il23a* | interleukin 23, alpha subunit p19 | 10.6 |
|  | *Il27* | interleukin 27 | 4.2 |
|  | *Il2ra* | interleukin 2 receptor, alpha chain | 2.8 |
|  | *Il2rg* | interleukin 2 receptor, gamma chain | 3.4 |
|  | *Il3ra* | interleukin 3 receptor, alpha chain | 2.6 |
|  | *Il6* | interleukin 6 | 17.5 |
|  | *Il6st* | interleukin 6 signal transducer | -2.3 |
|  | *Il7r* | interleukin 7 receptor | 6.8 |
|  | *Tnf* | tumor necrosis factor | 16.0 |
|  | *Csf1* | colony stimulating factor 1 (macrophage) | 8.0 |
|  | *Csf2* | colony stimulating factor 2 (granulocyte-macrophage) | 2.9 |
|  | *Csf2rb2* | colony stimulating factor 2 receptor, beta 2, low-affinity (granulocyte-macrophage) | 2.5 |
|  | *Csf3* | colony stimulating factor 3 (granulocyte) | 28.4 |
| **Pattern recognition receptors** |  |  |  |
|  | *Tlr2* | toll-like receptor 2 | 3.3 |
|  | *Tlr4* | toll-like receptor 4 | 2.8 |
|  | *Tlr7* | toll-like receptor 7 | 6.8 |
|  | *Tlr8* | toll-like receptor 8 | -2.8 |
|  | *Tlr9* | toll-like receptor 9 | 26.0 |
|  | *Nod1* | nucleotide-binding oligomerization domain containing 1 | 3.4 |
|  | *Nod2* | nucleotide-binding oligomerization domain containing 2 | 10.7 |
|  | *Nlrc4* | NLR family, CARD domain containing 4 | 2.8 |
|  | *Nlrc5* | NLR family, CARD domain containing 5 | 22.2 |
|  | *Nlrp3* | NLR family, pyrin domain containing 3 | 3.1 |
|  | *Clec4a1* | C-type lectin domain family 4, member a1 | 3.3 |
|  | *Clec4a4* | C-type lectin domain family 4, member a4 | 3.4 |
|  | *Clec4b2* | C-type lectin domain family 4, member b2 | 5.8 |
|  | *Clec4n* | C-type lectin domain family 4, member n | 10.0 |
|  | *Clec5a* | C-type lectin domain family 5, member a | 4.0 |
|  | *Cd209a* | CD209a antigen | -3.9 |
|  | *Mrc1* | mannose receptor, C type 1 | -6.5 |
| **Type I interferon-responsive genes and antiviral defense genes** |  |  |  |
|  | *Adar* | adenosine deaminase, RNA-specific | 3.7 |
|  | *Aim2* | absent in melanoma 2 | 4.4 |
|  | *Apobec1* | apolipoprotein B mRNA editing enzyme, catalytic polypeptide 1 | 2.1 |
|  | *Bcl2l1* | BCL2-like 1 | 2.4 |
|  | *Bcl2l11* | BCL2-like 11 (apoptosis facilitator) | 3.7 |
|  | *Bst2* | bone marrow stromal cell antigen 2 | 6.5 |
|  | *Ccl5* | chemokine (C-C motif) ligand 5 | 12.4 |
|  | *Cenpj* | centromere protein J | 2.8 |
|  | *Clec5a* | C-type lectin domain family 5, member a | 4.0 |
|  | *Ddx58* | DEAD (Asp-Glu-Ala-Asp) box polypeptide 58 | 3.1 |
|  | *Ddx60* | DEAD (Asp-Glu-Ala-Asp) box polypeptide 60 | 5.2 |
|  | *Dhx58* | DEXH (Asp-Glu-X-His) box polypeptide 58 | 6.7 |
|  | *Eif2ak2* | eukaryotic translation initiation factor 2-alpha kinase 2 | 3.9 |
|  | *Gbp2* | guanylate binding protein 2 | 5 |
|  | *Gbp3* | guanylate binding protein 3 | 21.5 |
|  | *Gm4951* | predicted gene 4951 | 8.0 |
|  | *Ifi204* | interferon activated gene 204 | 8.0 |
|  | *Ifi205* | interferon activated gene 205 | 40.7 |
|  | *Ifih1* | interferon induced with helicase C domain 1 | 6.8 |
|  | *Ifit1* | interferon-induced protein with tetratricopeptide repeats 1 | 13.9 |
|  | *Ifit2* | interferon-induced protein with tetratricopeptide repeats 2 | 49.0 |
|  | *Ifit3* | interferon-induced protein with tetratricopeptide repeats 3 | 9.3 |
|  | *Ifnar1* | interferon (alpha and beta) receptor 1 | 2.3 |
|  | *Iigp1* | interferon inducible GTPase 1 | 9.4 |
|  | *Irak3* | interleukin-1 receptor-associated kinase 3 | 5.5 |
|  | *Irf1* | interferon regulatory factor 1 | 3.3 |
|  | *Irf5* | interferon regulatory factor 5 | 5.0 |
|  | *Irf7* | interferon regulatory factor 7 | 5.2 |
|  | *Irf9* | interferon regulatory factor 9 | 2.5 |
|  | *Isg20* | interferon-stimulated protein | 2.6 |
|  | *Itgax* | integrin alpha X | 2.4 |
|  | *Jak2* | Janus kinase 2 | 3.3 |
|  | *Lif* | leukemia inhibitory factor | 3.3 |
|  | *Mb21d1* | Mab-21 domain containing 1 | 4.1 |
|  | *Mx1* | myxovirus (influenza virus) resistance 1 | 14.4 |
|  | *Mx2* | myxovirus (influenza virus) resistance 2 | 24.9 |
|  | *Nfkbia* | nuclear factor of kappa light polypeptide gene enhancer in B cells inhibitor, alpha | 2.6 |
|  | *Oas1a* | 2-5 oligoadenylate synthetase 1A | 7.5 |
|  | *Oas1b* | 2-5 oligoadenylate synthetase 1B | 5.6 |
|  | *Oas1c* | 2-5 oligoadenylate synthetase 1C | 3.5 |
|  | *Oas1g* | 2-5 oligoadenylate synthetase 1G | 5.6 |
|  | *Oas2* | 2-5 oligoadenylate synthetase 2 | 2.6 |
|  | *Oas3* | 2-5 oligoadenylate synthetase 3 | 2.3 |
|  | *Oasl1* | 2-5 oligoadenylate synthetase-like 1 | 8 |
|  | *Peli1* | pellino 1 | 2.7 |
|  | *Pmaip1* | phorbol-12-myristate-13-acetate-induced protein 1 | 8.5 |
|  | *Pml* | promyelocytic leukemia | 5 |
|  | *Pyhin1* | pyrin and HIN domain family, member 1 | 19.4 |
|  | *Rsad2* | radical S-adenosyl methionine domain containing 2 | 7.7 |
|  | *Snip1* | Smad nuclear interacting protein 1 | 2.2 |
|  | *Stat1* | signal transducer and activator of transcription 1 | 3.5 |
|  | *Tbk1* | TANK-binding kinase 1 | 5.8 |
|  | *Trim56* | tripartite motif-containing 56 | 3.3 |
|  | *Xaf1* | XIAP associated factor 1 | 9.8 |
|  | *Zbp1* | Z-DNA binding protein 1 | 6.6 |
| **IFN-γ responsive genes** |  |  |  |
|  | *Gbp5* | guanylate binding protein 5 | 50.0 |
|  | *Gbp3* | guanylate binding protein 3 | 21.5 |
|  | *Ccl2* | chemokine (C-C motif) ligand 2 | 16.4 |
|  | *Nos2* | nitric oxide synthase 2, inducible | 13.9 |
|  | *Ccl5* | chemokine (C-C motif) ligand 5 | 12.4 |
|  | *Parp9* | poly (ADP-ribose) polymerase family, member 9 | 6.1 |
|  | *Gbp2* | guanylate binding protein 2 | 5.0 |
|  | *Bst2* | bone marrow stromal cell antigen 2 | 6.5 |
|  | *Gbp7* | guanylate binding protein 7 | 4.7 |
|  | *Il12rb1* | interleukin 12 receptor, beta 1 | 4.6 |
|  | *Irgm1* | immunity-related GTPase family M member 1 | 11.0 |
|  | *Gbp8* | guanylate-binding protein 8 | 4.9 |
|  | *Jak2* | Janus kinase 2 | 3.3 |
|  | *Stat1* | signal transducer and activator of transcription 1 | 3.5 |
|  | *Irf1* | interferon regulatory factor 1 | 3.5 |
|  | *Cxcl16* | chemokine (C-X-C motif) ligand 16 | 2.1 |
|  | *Txk* | TXK tyrosine kinase | 2.5 |
|  | *Il12a* | interleukin 12a | 25.2 |
| **NF-κB target genes** |  |  |  |
|  | *Cxcl9* | chemokine (C-X-C motif) ligand 9 | 52.9 |
|  | *Il1a* | interleukin 1 alpha | 35.3 |
|  | *Cxcl10* | chemokine (C-X-C motif) ligand 10 | 26.8 |
|  | *Csf3* | colony stimulating factor 3 (granulocyte) | 28.4 |
|  | *Ccl4* | chemokine (C-C motif) ligand 4 | 23.5 |
|  | *Il10* | interleukin 10 | 23.2 |
|  | *Tnf* | tumor necrosis factor | 16.0 |
|  | *Il15ra* | interleukin 15 receptor, alpha chain | 18.0 |
|  | *Ccl2* | chemokine (C-C motif) ligand 2 | 16.4 |
|  | *Nos2* | nitric oxide synthase 2, inducible | 13.9 |
|  | *Olr1* | oxidized low density lipoprotein (lectin-like) receptor 1 | 11.0 |
|  | *Tnfrsf9* | tumor necrosis factor receptor superfamily, member 9 | 19.6 |
|  | *Orm1* | orosomucoid 1 | 10.8 |
|  | *Plau* | plasminogen activator, urokinase | 12.1 |
|  | *Csf1* | colony stimulating factor 1 (macrophage) | 8.0 |
|  | *Tnc* | tenascin C | 12.3 |
|  | *Ccl7* | chemokine (C-C motif) ligand 7 | 9.0 |
|  | *Nod2* | nucleotide-binding oligomerization domain containing 2 | 10.7 |
|  | *Il1rn* | interleukin 1 receptor antagonist | 6.2 |
|  | *Tap1* | transporter 1, ATP-binding cassette, sub-family B (MDR/TAP) | 6.4 |
|  | *Tnfaip3* | tumor necrosis factor, alpha-induced protein 3 | 6.2 |
|  | *Relb* | avian reticuloendotheliosis viral (v-rel) oncogene related B | 8.5 |
|  | *Cflar* | CASP8 and FADD-like apoptosis regulator | 6.6 |
|  | *Traf1* | TNF receptor-associated factor 1 | 6.3 |
|  | *Sod2* | superoxide dismutase 2, mitochondrial | 5.7 |
|  | *Gadd45b* | growth arrest and DNA-damage-inducible 45 beta | 5.7 |
|  | *Irf7* | interferon regulatory factor 7 | 5.2 |
|  | *Cd40* | CD40 antigen | 6.3 |
|  | *Ccl3* | chemokine (C-C motif) ligand 3 | 5.1 |
|  | *Ccr5* | chemokine (C-C motif) receptor 5 | 5.1 |
|  | *Hmox1* | heme oxygenase (decycling) 1 | 4.4 |
|  | *Fas* | Fas (TNF receptor superfamily member 6) | 4.6 |
|  | *Ier3* | immediate early response 3 | 5.0 |
|  | *Nfkb2* | nuclear factor of kappa light polypeptide gene enhancer in B cells 2, p49/p100 | 4.5 |
|  | *Icam1* | intercellular adhesion molecule 1 | 3.9 |
|  | *Psmb9* | proteasome (prosome, macropain) subunit, beta type 9 (large multifunctional peptidase 2 | 4.0 |
|  | *Cxcl5* | chemokine (C-X-C motif) ligand 5 | 4.3 |
|  | *Lta* | lymphotoxin A | 3.2 |
|  | *Irf1* | interferon regulatory factor 1 | 3.5 |
|  | *Csf2* | colony stimulating factor 2 (granulocyte-macrophage) | 2.9 |
|  | *Cd48* | CD48 antigen | 3.5 |
|  | *Casp4* | caspase 4, apoptosis-related cysteine peptidase | 3.0 |
|  | *Rel* | reticuloendotheliosis oncogene | 2.5 |
|  | *Bax* | BCL2-associated X protein | 3.1 |
|  | *Il2ra* | interleukin 2 receptor, alpha chain | 2.8 |
|  | *Nqo1* | NAD(P)H dehydrogenase, quinone 1 | 3.0 |
|  | *Nfkbia* | nuclear factor of kappa light polypeptide gene enhancer in B cells inhibitor, alpha | 2.6 |
|  | *Nfkb1* | nuclear factor of kappa light polypeptide gene enhancer in B cells 1, p105 | 2.7 |
|  | *Tapbp* | TAP binding protein | 2.6 |
|  | *Bcl2l1* | BCL2-like 1 | 2.4 |
|  | *Traf2* | TNF receptor-associated factor 2 | 2.2 |
|  | *Birc2* | baculoviral IAP repeat-containing 2 | 2.4 |
| **NF-κB regulated transcription factors** |  |  |  |
|  | *Nfkbie* | nuclear factor of kappa light polypeptide gene enhancer in B cells inhibitor, epsilon | 7.9 |
|  | *Tnfaip3* | tumor necrosis factor, alpha-induced protein 3 | 6.2 |
|  | *Relb* | avian reticuloendotheliosis viral (v-rel) oncogene related B | 8.5 |
|  | *Tfec* | transcription factor EC | 6.3 |
|  | *Tnip3* | TNFAIP3 interacting protein 3 | 7.2 |
|  | *Nfkb2* | nuclear factor of kappa light polypeptide gene enhancer in B cells 2, p49/p100 | 4.5 |
|  | *Irf1* | interferon regulatory factor 1 | 3.5 |
|  | *Rel* | reticuloendotheliosis oncogene | 2.5 |
|  | *Nfkbia* | nuclear factor of kappa light polypeptide gene enhancer in B cells inhibitor, alpha | 2.6 |
|  | *Nfkb1* | nuclear factor of kappa light polypeptide gene enhancer in B cells 1, p105 | 2.7 |
| **IKK and IKK-related protein signaling complex members** |  |  |  |
|  |  | **IKK-family kinases** |  |
|  | *Ikbke* (IKKε) | inhibitor of kappaB kinase epsilon | 30.9 |
|  | *Tbk1* | TANK-binding kinase 1 | 5.8 |
|  | *Chuk* (IKKα) | conserved helix-loop-helix ubiquitous kinase | -1.2 |
|  | *Ikbkb* (IKKβ) | inhibitor of kappaB kinase beta | 1.1 |
|  |  |  |  |
|  |  | **IKK-kinase scaffolds** |  |
|  | *Optn* | optineurin | 3.4 |
|  | *Azi2* | 5-azacytidine induced gene 2 | 3.3 |
|  | *Tbkbp1* | TBK1 binding protein 1 | 2.2 |
|  | *Ikbkg* | inhibitor of kappaB kinase gamma | 2.2 |
|  | *Tank* | TRAF family member-associated Nf-kappa B activator | 2.0 |
|  | *Sqstm1* | sequestosome 1 | 1.6 |
|  | *Riok3* | RIO kinase 3 | 1.6 |
|  | *Dok3* | docking protein 3 | 1.3 |
|  | *Calcoco2* | calcium binding and coiled-coil domain 2 | -1.3 |
|  |  |  |  |
|  |  | **RLR and nucleic acid sensors** |  |
|  | *Zbp1* | Z-DNA binding protein 1 (DAI) | 6.6 |
|  | *Ddx58* | DEAD (Asp-Glu-Ala-Asp) box polypeptide 58 (RIG-I) | 3.1 |
|  | *Ifih1* | interferon induced with helicase C domain 1 (MDA5) | 6.8 |
|  | *Dhx58* | DEXH (Asp-Glu-X-His) box polypeptide 58 (LGP2) | 6.7 |
|  | *Mavs* | mitochondrial antiviral signaling protein | -3.0 |
|  | *Tmem173* | transmembrane protein 173 (STING) | 1.5 |
|  |  |  |  |
|  |  | **TRAFs** |  |
|  | *Traf1* | TNF receptor-associated factor 1 | 6.3 |
|  | *Traf2* | TNF receptor-associated factor 2 | 2.2 |
|  | *Traf3* | TNF receptor-associated factor 3 | 5.1 |
|  | *Traf4* | TNF receptor associated factor 4 | -2.3 |
|  | *Traf5* | TNF receptor-associated factor 5 | -1.8 |
|  | *Traf6* | TNF receptor-associated factor 6 | 4 |
|  | *Traf7* | TNF receptor-associated factor 7 | 1.0 |
| **AP-1 regulated genes** |  |  |  |
|  | *Nos2* | nitric oxide synthase 2, inducible | 13.9 |
|  | *Timp1* | tissue inhibitor of metalloproteinase 1 | 13.4 |
|  | *Csf1* | colony stimulating factor 1 (macrophage) | 8.0 |
|  | *Relb* | avian reticuloendotheliosis viral (v-rel) oncogene related B | 8.5 |
|  | *Spp1* | secreted phosphoprotein 1 | 5.9 |
|  | *Il7r* | interleukin 7 receptor | 6.8 |
|  | *Tnfrsf8* | tumor necrosis factor receptor superfamily, member 8 | 5.8 |
|  | *Hmox1* | heme oxygenase (decycling) 1 | 4.4 |
|  | *Acp5* | acid phosphatase 5, tartrate resistant | 4.5 |
|  | *Egr2* | early growth response 2 | 4.1 |
|  | *Atp10a* | ATPase, class V, type 10A | 3.6 |
|  | *Gba* | glucosidase, beta, acid | 3.2 |
|  | *Tlr4* | toll-like receptor 4 | 2.8 |
|  | *Tapbp* | TAP binding protein | 2.6 |
|  | *Slc20a1* | solute carrier family 20, member 1 | 2.5 |
|  | *Gzmb* | granzyme B | 2.8 |
| **Nrf2 regulated genes** |  |  |  |
|  |  | **Upregulated Nrf2-regulated genes** |  |
|  | *Il6* | interleukin 6 | 17.5 |
|  | *Sod2* | superoxide dismutase 2, mitochondrial | 5.7 |
|  | *Itga5* | integrin alpha 5 (fibronectin receptor alpha) | 5.2 |
|  | *Hmox1* | heme oxygenase (decycling) 1 | 4.4 |
|  | *F7* | coagulation factor VII | 5.6 |
|  | *Ctss* | cathepsin S | 4.3 |
|  | *Hk2* | hexokinase 2 | 4.9 |
|  | *Egr2* | early growth response 2 | 4.1 |
|  | *Nfkbib* | nuclear factor of kappa light polypeptide gene enhancer in B cells inhibitor, beta | 3.2 |
|  | *Gadd45g* | growth arrest and DNA-damage-inducible 45 gamma | 3.3 |
|  | *Nqo1* | NAD(P)H dehydrogenase, quinone 1 | 3.0 |
|  | *Txnrd1* | thioredoxin reductase 1 | 2.5 |
|  | *Socs3* | suppressor of cytokine signaling 3 | 2.8 |
|  | *Esd* | esterase D/formylglutathione hydrolase | 2.1 |
|  | *Ptpn2* | protein tyrosine phosphatase, non-receptor type 2 | 1.9 |
|  | *Clec4d* | C-type lectin domain family 4, member d | 1.8 |
|  | *Ptpn6* | protein tyrosine phosphatase, non-receptor type 6 | 2.0 |
|  | *Atf1* | activating transcription factor 1 | 1.8 |
|  | *Egr1* | early growth response 1 | 1.6 |
|  | Wisp1 | WNT1 inducible signaling pathway protein 1 | 1.6 |
|  | Ccr1 | chemokine (C-C motif) receptor 1 | 1.5 |
|  | *Hif1a* | hypoxia inducible factor 1, alpha subunit | 1.6 |
|  | *Klf6* | Kruppel-like factor 6 | 1.6 |
|  |  |  |  |
|  |  | **Downregulated Nrf2 regulated genes** |  |
|  | *Cyp2s1* | cytochrome P450, family 2, subfamily s, polypeptide 1 | -8.7 |
|  | *Aldh3a1* | aldehyde dehydrogenase family 3, subfamily A1 | -7.4 |
|  | *Hsph1* | heat shock 105kDa/110kDa protein 1 | -7.1 |
|  | *Aldh1a1* | aldehyde dehydrogenase family 1, subfamily A1 | -6.8 |
|  | *Ldhb* | lactate dehydrogenase B | -6.3 |
|  | *Aqp1* | aquaporin 1 | -6.1 |
|  | *Hspa1a* | heat shock protein 1A | -4.9 |
|  | *Idh2* | isocitrate dehydrogenase 2 (NADP+), mitochondrial | -4.4 |
|  | *Ptpn14* | protein tyrosine phosphatase, non-receptor type 14 | -4.4 |
|  | *Aox1* | aldehyde oxidase 1 | -4.3 |
|  | *Gstt1* | glutathione S-transferase, theta 1 | -4.2 |
|  | *Aldh7a1* | aldehyde dehydrogenase family 7, member A1 | -4.0 |
|  | *Tgfb2* | transforming growth factor, beta 2 | -4.1 |
|  | *Cat* | catalase | -3.9 |
|  | *Hspa1b* | heat shock protein 1B | -3.8 |
|  | *Cyp2d22* | cytochrome P450, family 2, subfamily d, polypeptide 22 | -3.7 |
|  | *Cbr3* | carbonyl reductase 3 | -3.4 |
|  | *Gsto1* | glutathione S-transferase omega 1 | -3.4 |
|  | *Gstm2* | glutathione S-transferase, mu 2 | -3.1 |
|  | *Prkca* | protein kinase C, alpha | -3.2 |
|  | *Nr4a2* | nuclear receptor subfamily 4, group A, member 2 | -2.9 |
|  | *Gpx2* | glutathione peroxidase 2 | -3.0 |
|  | *Pparg* | peroxisome proliferator activated receptor gamma | -2.8 |
|  | *Emcn* | endomucin | -2.5 |
|  | *Akr1b10* | aldo-keto reductase family 1, member B10 (aldose reductase) | -2.4 |
|  | *Gss* | glutathione synthetase | -2.3 |
|  | *Dnajb1* | DnaJ (Hsp40) homolog, subfamily B, member 1 | -2.1 |
|  | *Mdh1* | malate dehydrogenase 1, NAD (soluble) | -1.9 |
|  | *Aldh9a1* | aldehyde dehydrogenase 9, subfamily A1 | -1.9 |
|  | *Gjb3* | gap junction protein, beta 3 | -1.8 |

Genes are listed based on categories according to the literature and publicly available databases. Genes with fold changes greater than 2 and FDR<0.05 are considered to be differentially expressed (DE). FDR<0.05 except underlined genes, where FDR>0.05.

**Supplementary Table 2.** Expression of mRNA genes in lung neutrophils measured using RT-PCR

| **mRNA** | **Threshold cycle (Ct)** | | **Delta Threshold Cycle (dCt)** | | **Mean fold change**  **SP compared to PBS (range)** | **Fold change**  **SP compared to PBS in gene profiling study** |
| --- | --- | --- | --- | --- | --- | --- |
|  | **PBS** | ***S. pneumoniae*** | **PBS** | ***S. pneumoniae*** |  |  |
| ***Cxcl1*** | 34.6 ± 0.2 | 29.2 ± 0.4* | 16.1 ±0.4 | 11.0 ± 0.4* | 33 (19-60)* | 7.7† |
| ***Cxcl5*** | nd | 33.2 | n/a | 12.9 | >76 ^‡^ | 4.3† |
| ***Cxcl9*** | 38.9 ± 0.4 (n=4, nd=1) | 29.5 ± 0.2* | 20.7 ±0.3 | 11.3 ± 0.2* | 647 (540-871)* | 53† |
| ***Cxcl11*** | n.d. | 29.0 ± 0.2 | n/a | 10.8 ± 0.3 | >2000^‡^ | 47† |
| ***Hmox1*** | 32.3 ± 0.2 | 29.4 ± 0.3* | 13.8 ±0.2 | 11.2 ± 0.2* | 6.1 (4.1-8.3)* | 4.4† |
| ***Ifit1*** | nd | 32 | n/a | n/a | >100 ^‡^ | 14† |
| ***Ifnar2*** | 29.1 ± 0.2 | 27.8 ± 0.1* | 10.6 ±0.1 | 9.7 ± 0.1* | 1.9 (1.7-2.2)* | 1.5† |
| ***Ikbke*** | nd | 34.4 | n/a | n/a | >22 ^‡^ | 31† |
| ***Il1a*** | 36.1 ± 0.4 | 25.8 ± 0.1* | 17.1 ±0.6 | 6.9 ± 0.2* | 1156 (715-1263)* | 35† |
| ***Il1b*** | 28.1 ± 0.3 | 26.3 ± 0.2* | 9.1 ±0.3 | 7.5 ± 0.2* | 3.0 (1.9-4.0)* | 1.1 ns |
| ***Il6*** | 36.2 ± 0.3 (n=4, nd=1) | 29.2 ± 0.7* | 17.2 ±0.6 | 10.4 ± 0.6* | 112 (46-452)* | 17.5† |
| ***Il10*** | 36.3 ± 0.3 (n=4, nd=1) | 33.4 ± 0.4* | 17.6 ±0.2 | 15.2 ± 0.4* | 5.2 (2.1-10.4)* | 23† |
| ***Irf5*** | nd | 34.1 | n/a | n/a | >23 ^‡^ | 5† |
| ***Isg15*** | 32.0 ± 0.2 | 27.1 ± 0.1* | 13.0 ±0.3 | 8.2 ± 0.2* | 28 (19-42)* | n/a |
| ***Nqo1*** | 34.2 ± 0.4 | 33.0 ± 0.1* | 15.2 ±0.2 | 14.2 ± 0.1* | 2.0 (1.5-2.4)* | 3† |
| ***Nos2*** | 38.1 ± 0.3 | 33.7 ± 0.5* | 16.3 ± 0.2 | 13.5 ± 0.4* | 7.1 (4.4-17)* | 14† |
| ***Tbk1*** | 36.6 ± 0.5 | 32.3 ± 0.3* | 14.8 ±0.4 | 12.1 ± 0.1* | 6.5 (5.5-9.2)* | 5.8† |
| ***Tnf*** | 33.0 ± 0.2 | 26.4 ± 0.0* | 14.0 ±0.1 | 7.5 ± 0.1* | 89 (80-107)* | 16† |

Gene expression was measured using TaqMan gene expression assays and RT-qPCR in independent samples generated from a separate study. Ct and dCt values were determined as described in the methods. Values for Ct and dCT are mean ± SEM. Mean Ct was calculated from threshold values when detected at <40 cycles; Ct for *Ifit1* *Ikbke* and *Irf5* are denoted not detectable (nd) because gene expression in at least 3 out of 5 samples per group did not cross the expression threshold after 40 PCR amplification cycles. Fold changes in gene expression in SP samples were calculated with the PBS group being equal to 1 (values in parenthesis denote the range of fold changes in individual *S. pneumoniae*-treated samples compared with the mean of the PBS group). n=5 per group unless indicated otherwise. nd, not detectable (>40 cycles); n/a, not applicable; *p<0.05 compared with PBS group, t test; †FDR <0.05, SP compared to PBS in the gene profiling study using microarrays; ^‡^ calculated fold change based on imputed C_T_ of 41 for the PBS group.

**Supplementary Table 3.** Protein expression of selected markers and cytokines in lung neutrophils

| **Protein** | **Fluorescence Intensity** | |
| --- | --- | --- |
| *Surface Protein* | **PBS** | ***S. pneumoniae*** |
| CD11b | 429,424 ± 53,634 | 570,064 ± 16,115 |
| **CD11c** | 4,382 ± 735 | 21,971 ± 1,183* |
| CD24 | 101,855 ± 11,249 | 149,447 ± 16,624* |
| CD45 | 62,431 ± 4,709 | 58,176 ± 1,439 |
| **CD54** | 15,087 ±1,757 | 44,303 ± 2,585* |
| **CD64** | 46,315 ± 5,956 | 73,137 ± 6,298* |
| **CD103** | 30,024 ± 3,875 | 45,770 ± 2,365* |
| Ly6C | 41,107 ±5,721 | 113,550 ± 7,729* |
| *Intracellular Cytokine* |  |  |
| **IFNγ** | 10,361 ± 661 | 17,239 ± 1415* |
| **TNF** | 9,699 ± 50 | 18,945 ± 2159* |
|  | **Percentage of lung neutrophils expressing intracellular cytokine** | |
| *Intracellular Cytokine* | **PBS** | **SP** |
| **IFNγ** | 20 ± 2.5% | 45 ± 5.4% |
| **TNF** | 17 ± 1.5% | 51 ± 7.9% |

Fluorescence intensity of surface markers and intracellular staining for IFNγ and TNF in lung neutrophils from mice that received PBS or *S. pneumoniae*. * p<0.05 vs PBS ( t test). Values are mean ± SEM, n=4 (PBS) or 6 (*S. pneumoniae*). The genes encoding the proteins in bold are upregulated during *S. pneumoniae* pneumonia and designated as DE (FDR<0.05, fold change >2): *Itgax* (CD11c), 2.4 fold; *Icam1* (CD54), 3.9 fold; *Fcgr1* (CD64), 32.6 fold; *Itgae* (CD103), 2.1 fold; *Ifng*,2.3 fold; and *Tnf*, 16 fold.

**Supplementary Table 4.** 98 consensus DE miRs.

| **Family accession number** | **Family miRBase 21 name** | **miR accession number** | **miR name** | **RMA step up corrected p-value (SP vs. PBS)** | **RMA Fold-Change (SP vs. PBS)** | **LVS step up corrected p-value (SP vs. PBS)** | **LVS Fold-Change (SP vs. PBS)** |
| --- | --- | --- | --- | --- | --- | --- | --- |
| MIPF0001981 | mir-1896 | MIMAT0007873 | mmu-miR-1896 | 4.05E-03 | 21.7 | 4.87E-03 | 14.9 |
| MIPF0000669 | mir-1247 | MIMAT0014801 | mmu-miR-1247-3p | 2.09E-02 | 21.2 | 3.90E-02 | 8.2 |
|  |  | MIMAT0014908 | mmu-miR-3093-3p | 1.81E-02 | 22.5 | 4.34E-02 | 6.8 |
| MIPF0001271 | mir-3960 | MIMAT0019336 | mmu-miR-3960 | 2.91E-04 | 5.4 | 4.87E-03 | 4.5 |
|  | mir-1224 | MIMAT0005460 | mmu-miR-1224-5p | 5.20E-05 | 5.1 | 3.41E-03 | 4.3 |
| MIPF0000316 | mir-467 | MIMAT0002108 | mmu-miR-467a-3p | 2.02E-02 | 6.6 | 3.19E-02 | 4.2 |
| MIPF0000113 | mir-188 | MIMAT0000217 | mmu-miR-188-5p | 1.22E-03 | 4.7 | 2.52E-03 | 3.5 |
| MIPF0000191 | mir-340 | MIMAT0000586 | mmu-miR-340-3p | 1.01E-02 | 4.0 | 1.97E-02 | 3.3 |
|  |  | MIMAT0014903 | mmu-miR-3091-5p | 4.75E-03 | 5.8 | 4.87E-03 | 3.1 |
| MIPF0000117 | mir-139 | MIMAT0000656 | mmu-miR-139-5p | 6.78E-03 | 3.5 | 1.65E-02 | 3.0 |
| MIPF0000180 | mir-483 | MIMAT0004782 | mmu-miR-483-5p | 2.37E-02 | 5.2 | 2.12E-02 | 3.0 |
| MIPF0000028 | mir-135 | MIMAT0004531 | mmu-miR-135a-1-3p | 2.51E-03 | 6.7 | 8.20E-03 | 2.9 |
|  |  | MIMAT0009459 | mmu-miR-1982-5p | 1.61E-02 | 3.7 | 1.18E-02 | 2.8 |
|  |  | MIMAT0014872 | mmu-miR-3082-5p | 6.75E-03 | 3.3 | 2.84E-02 | 2.6 |
| MIPF0000963 | mir-2861 | MIMAT0013803 | mmu-miR-2861 | 3.56E-03 | 3.3 | 1.16E-02 | 2.5 |
| MIPF0000117 | mir-139 | MIMAT0004662 | mmu-miR-139-3p | 4.02E-03 | 4.4 | 1.26E-02 | 2.5 |
| MIPF0000006 | mir-15 | MIMAT0000124 | mmu-miR-15b-5p | 1.12E-02 | 3.2 | 1.82E-02 | 2.4 |
| MIPF0000316 | mir-467 | MIMAT0017325 | mmu-miR-466i-5p | 1.03E-02 | 2.8 | 3.70E-02 | 2.3 |
| MIPF0000338 | mir-680 | MIMAT0003457 | mmu-miR-680 | 5.46E-03 | 3.0 | 1.91E-02 | 2.3 |
| MIPF0000042 | mir-204 | MIMAT0017059 | mmu-miR-211-3p | 2.17E-03 | 2.8 | 8.84E-03 | 2.3 |
|  |  | MIMAT0020637 | mmu-miR-5126 | 4.02E-03 | 2.5 | 3.14E-02 | 2.3 |
| MIPF0000060 | mir-21 | MIMAT0004628 | mmu-miR-21a-3p | 7.00E-04 | 3.3 | 1.15E-02 | 2.2 |
|  |  | MIMAT0003515 | mmu-miR-721 | 2.85E-02 | 2.8 | 3.90E-02 | 2.2 |
|  |  | MIMAT0014862 | mmu-miR-3077-5p | 7.00E-04 | 5.7 | 9.61E-03 | 2.2 |
| MIPF0000419 | mir-574 | MIMAT0004893 | mmu-miR-574-5p | 8.55E-03 | 2.7 | 4.78E-02 | 2.1 |
| MIPF0000274 | mir-149 | MIMAT0016990 | mmu-miR-149-3p | 3.69E-04 | 3.7 | 1.80E-03 | 2.1 |
| MIPF0001469 | mir-3102 | MIMAT0014933 | mmu-miR-3102-5p | 1.22E-03 | 3.0 | 1.19E-02 | 2.1 |
| MIPF0000001 | mir-17 | MIMAT0000528 | mmu-miR-18a-5p | 6.00E-03 | 2.6 | 1.45E-02 | 2.1 |
| MIPF0000034 | mir-130 | MIMAT0000387 | mmu-miR-130b-3p | 1.54E-02 | 3.0 | 3.42E-02 | 2.1 |
|  |  | MIMAT0007864 | mmu-miR-1897-5p | 1.00E-02 | 2.6 | 2.63E-02 | 2.0 |
| MIPF0000204 | mir-297 | MIMAT0004865 | mmu-miR-297c-5p | 2.84E-02 | 2.9 | 4.94E-02 | 2.0 |
| MIPF0000126 | mir-379 | MIMAT0000743 | mmu-miR-379-5p | 4.08E-03 | -2.3 | 1.80E-03 | -2.0 |
| MIPF0000041 | mir-24 | MIMAT0005440 | mmu-miR-24-2-5p | 1.88E-02 | -2.1 | 3.18E-02 | -2.2 |
| MIPF0001489 | mir-3075 | MIMAT0014858 | mmu-miR-3075-5p | 1.88E-02 | -2.2 | 7.88E-03 | -2.2 |
| MIPF0000038 | mir-1 | MIMAT0000123 | mmu-miR-1a-3p | 8.48E-03 | -3.7 | 1.40E-02 | -2.3 |
|  |  | MIMAT0019349 | mmu-miR-101c | 2.28E-02 | -2.1 | 2.74E-02 | -2.4 |
| MIPF0000019 | mir-8 | MIMAT0004619 | mmu-miR-200a-5p | 6.32E-03 | -3.6 | 9.61E-03 | -2.4 |
|  |  | MIMAT0015643 | mmu-miR-3472 | 5.13E-03 | -2.2 | 8.26E-03 | -2.5 |
| MIPF0000064 | mir-31 | MIMAT0004634 | mmu-miR-31-3p | 1.06E-02 | -3.8 | 1.07E-02 | -2.5 |
| MIPF0000108 | mir-203 | MIMAT0000236 | mmu-miR-203-3p | 1.81E-02 | -2.8 | 1.88E-02 | -2.5 |
| MIPF0000057 | mir-28 | MIMAT0019339 | mmu-miR-28c | 8.96E-03 | -2.6 | 9.61E-03 | -2.5 |
| MIPF0000199 | mir-331 | MIMAT0000571 | mmu-miR-331-3p | 4.75E-03 | -2.2 | 1.04E-02 | -2.6 |
| MIPF0000002 | let-7 | MIMAT0000523 | mmu-let-7c-5p | 7.99E-03 | -2.6 | 4.00E-02 | -2.6 |
| MIPF0000093 | mir-144 | MIMAT0000156 | mmu-miR-144-3p | 1.88E-02 | -4.1 | 1.15E-02 | -2.6 |
| MIPF0001230 | mir-3473 | MIMAT0015645 | mmu-miR-3473a | 2.17E-03 | -2.9 | 4.87E-03 | -2.6 |
| MIPF0000901 | mir-3470 | MIMAT0015640 | mmu-miR-3470a | 2.51E-03 | -2.5 | 6.22E-03 | -2.7 |
| MIPF0000417 | mir-582 | MIMAT0005291 | mmu-miR-582-5p | 4.82E-03 | -2.6 | 1.10E-02 | -2.7 |
| MIPF0000027 | mir-23 | MIMAT0000125 | mmu-miR-23b-3p | 3.79E-03 | -2.5 | 1.65E-02 | -2.8 |
| MIPF0000066 | mir-183 | MIMAT0000212 | mmu-miR-183-5p | 3.01E-02 | -3.6 | 1.65E-02 | -2.8 |
| MIPF0000391 | mir-672 | MIMAT0003735 | mmu-miR-672-5p | 1.77E-02 | -3.7 | 1.62E-02 | -3.0 |
| MIPF0000002 | let-7 | MIMAT0000383 | mmu-let-7d-5p | 1.13E-02 | -2.1 | 1.65E-02 | -3.0 |
| MIPF0000076 | mir-190 | MIMAT0004852 | mmu-miR-190b-5p | 3.07E-02 | -5.1 | 3.40E-02 | -3.0 |
| MIPF0000019 | mir-8 | MIMAT0004545 | mmu-miR-200b-5p | 6.32E-03 | -4.0 | 7.88E-03 | -3.1 |
| MIPF0000079 | mir-145 | MIMAT0000157 | mmu-miR-145a-5p | 4.36E-03 | -5.5 | 3.37E-03 | -3.1 |
| MIPF0000007 | mir-181 | MIMAT0000673 | mmu-miR-181b-5p | 3.32E-03 | -3.1 | 4.87E-03 | -3.2 |
| MIPF0000002 | let-7 | MIMAT0000522 | mmu-let-7b-5p | 4.23E-02 | -2.3 | 2.06E-02 | -3.2 |
| MIPF0001230 | mir-3473 | MIMAT0020367 | mmu-miR-3473b | 1.09E-02 | -2.6 | 1.19E-02 | -3.2 |
| MIPF0000005 | mir-30 | MIMAT0005438 | mmu-miR-30c-2-3p | 2.17E-03 | -4.3 | 1.80E-03 | -3.2 |
| MIPF0000036 | mir-27 | MIMAT0000126 | mmu-miR-27b-3p | 4.75E-03 | -2.6 | 1.04E-02 | -3.2 |
| MIPF0000168 | mir-378 | MIMAT0019348 | mmu-miR-378b | 6.75E-03 | -4.4 | 1.04E-02 | -3.4 |
| MIPF0000002 | let-7 | MIMAT0000525 | mmu-let-7f-5p | 6.00E-03 | -2.4 | 1.56E-02 | -3.4 |
| MIPF0000197 | mir-150 | MIMAT0000160 | mmu-miR-150-5p | 3.19E-02 | -4.3 | 1.68E-02 | -3.4 |
| MIPF0000005 | mir-30 | MIMAT0000514 | mmu-miR-30c-5p | 1.22E-03 | -2.6 | 5.51E-03 | -3.6 |
| MIPF0000026 | mir-218 | MIMAT0000663 | mmu-miR-218-5p | 3.67E-02 | -3.8 | 3.42E-02 | -3.6 |
| MIPF0000033 | mir-10 | MIMAT0000135 | mmu-miR-125a-5p | 2.09E-02 | -3.2 | 1.18E-02 | -3.7 |
| MIPF0000039 | mir-34 | MIMAT0000542 | mmu-miR-34a-5p | 8.77E-03 | -2.9 | 1.19E-02 | -3.7 |
| MIPF0000019 | mir-8 | MIMAT0000657 | mmu-miR-200c-3p | 1.27E-02 | -3.4 | 1.92E-02 | -3.8 |
| MIPF0000231 | mir-497 | MIMAT0003453 | mmu-miR-497-5p | 1.63E-02 | -3.8 | 3.41E-02 | -3.8 |
| MIPF0000196 | mir-335 | MIMAT0000766 | mmu-miR-335-5p | 7.00E-04 | -7.4 | 1.16E-03 | -3.9 |
| MIPF0000082 | mir-193 | MIMAT0004859 | mmu-miR-193b-3p | 1.88E-02 | -5.4 | 1.19E-02 | -3.9 |
| MIPF0000056 | mir-148 | MIMAT0000162 | mmu-miR-152-3p | 2.21E-02 | -3.8 | 2.63E-02 | -3.9 |
| MIPF0000168 | mir-378 | MIMAT0003151 | mmu-miR-378a-3p | 4.05E-03 | -3.6 | 4.69E-03 | -3.9 |
| MIPF0000399 | mir-872 | MIMAT0004934 | mmu-miR-872-5p | 6.09E-03 | -4.1 | 9.52E-03 | -4.1 |
|  |  | MIMAT0003469 | mmu-miR-690 | 2.68E-02 | -3.2 | 2.16E-02 | -4.1 |
| MIPF0000006 | mir-15 | MIMAT0000225 | mmu-miR-195a-5p | 2.37E-02 | -2.9 | 2.51E-02 | -4.2 |
| MIPF0000115 | mir-126 | MIMAT0000138 | mmu-miR-126a-3p | 4.36E-03 | -3.9 | 1.32E-02 | -4.2 |
| MIPF0000057 | mir-28 | MIMAT0004536 | mmu-miR-151-5p | 1.20E-02 | -3.7 | 1.66E-02 | -4.3 |
| MIPF0000034 | mir-130 | MIMAT0000141 | mmu-miR-130a-3p | 1.45E-02 | -4.1 | 2.63E-02 | -4.4 |
| MIPF0000033 | mir-10 | MIMAT0000132 | mmu-miR-99b-5p | 2.71E-02 | -4.7 | 1.76E-02 | -4.7 |
| MIPF0000064 | mir-31 | MIMAT0000538 | mmu-miR-31-5p | 1.98E-02 | -5.9 | 1.95E-02 | -4.8 |
| MIPF0000005 | mir-30 | MIMAT0000129 | mmu-miR-30a-3p | 7.99E-03 | -4.3 | 1.03E-02 | -4.9 |
| MIPF0000039 | mir-34 | MIMAT0004580 | mmu-miR-34c-3p | 9.03E-03 | -6.3 | 1.32E-02 | -5.0 |
| MIPF0000005 | mir-30 | MIMAT0000128 | mmu-miR-30a-5p | 4.08E-03 | -4.1 | 9.52E-03 | -5.0 |
| MIPF0000072 | mir-96 | MIMAT0000541 | mmu-miR-96-5p | 9.66E-03 | -5.2 | 1.19E-02 | -5.1 |
| MIPF0000002 | let-7 | MIMAT0000524 | mmu-let-7e-5p | 2.09E-02 | -3.8 | 1.82E-02 | -5.2 |
| MIPF0000033 | mir-10 | MIMAT0000136 | mmu-miR-125b-5p | 3.23E-02 | -4.0 | 2.63E-02 | -6.0 |
| MIPF0000114 | mir-375 | MIMAT0000739 | mmu-miR-375-3p | 1.14E-02 | -6.5 | 1.32E-02 | -6.3 |
| MIPF0000019 | mir-8 | MIMAT0000233 | mmu-miR-200b-3p | 6.88E-03 | -7.2 | 1.23E-02 | -6.7 |
| MIPF0000019 | mir-8 | MIMAT0001537 | mmu-miR-429-3p | 9.73E-03 | -6.2 | 1.15E-02 | -6.9 |
| MIPF0000094 | mir-143 | MIMAT0000247 | mmu-miR-143-3p | 1.16E-02 | -6.4 | 1.31E-02 | -7.1 |
| MIPF0000019 | mir-8 | MIMAT0000153 | mmu-miR-141-3p | 9.66E-03 | -5.4 | 1.28E-02 | -7.2 |
| MIPF0000115 | mir-126 | MIMAT0000137 | mmu-miR-126a-5p | 6.32E-03 | -6.9 | 8.37E-03 | -7.8 |
| MIPF0000039 | mir-34 | MIMAT0000382 | mmu-miR-34b-5p | 7.14E-03 | -6.7 | 1.31E-02 | -8.0 |
| MIPF0000019 | mir-8 | MIMAT0000519 | mmu-miR-200a-3p | 8.66E-03 | -7.9 | 1.30E-02 | -8.5 |
| MIPF0000039 | mir-34 | MIMAT0000381 | mmu-miR-34c-5p | 6.78E-03 | -8.7 | 1.15E-02 | -9.2 |
| MIPF0000133 | mir-449 | MIMAT0001542 | mmu-miR-449a-5p | 3.81E-03 | -9.8 | 4.83E-03 | -10.1 |
| MIPF0000039 | mir-34 | MIMAT0004581 | mmu-miR-34b-3p | 1.06E-02 | -11.5 | 1.53E-02 | -17.8 |
| MIPF0000088 | mir-224 | MIMAT0000671 | mmu-miR-224-5p | 7.52E-03 | -6.9 | 6.22E-03 | -30.8 |

DE miRs were changed at least 2-fold in both RMA- and LVS-normalized data sets and were considered to be the consensus DE miRs. They were sorted by LVS fold change during pneumonia compared with PBS after LVS normalization. Shaded rows indicate upregulated miRs.

**Supplementary Table 5.** Expression of selected miRs in lung neutrophils measured using RT-qPCR

| **miR** | **Ct** | | **dCt** | | **Mean fold change expressed as ratio of *S. pneumoniae* to PBS (range)** | **Fold change expressed as ratio (signed fold change) of *S. pneumoniae* group compared to PBS group in the microarray gene profiling study** | |
| --- | --- | --- | --- | --- | --- | --- | --- |
|  | **PBS** | ***S. pneumoniae*** | **PBS** | ***S. pneumoniae*** |  | **LVS** | **RMA** |
| mmu-let-7b-5p | 27.3 ± 0.42 | 27.6 ± 0.20 | 0.50 ± 0.30 | 1.03 ± 0.27 | 0.69 (0.34-1.04) | 0.31  (-3.2)† | 0.43  (-2.3)† |
| mmu-let-7c-5p | 27.7 ± 0.40 | 27.9 ± 0.19 | 0.88 ± 0.27 | 1.34 ± 0.25 | 0.73 (0.39-1.13) | 0.38  (-2.6)† | 0.38  (-2.6)† |
| mmu-let-7d-5p | 28.6 ± 0.31 | 28.5 ± 0.14 | 1.83 ± 0.18 | 1.96 ± 0.21 | 0.91 (0.54-1.3) | 0.33  (-3.0)† | 0.47  (-2.1)† |
| mmu-let-7f-5p | 27.6 ± 0.34 | 27.8 ± 0.12 | 0.78 ± 0.21 | 1.29 ± 0.17 | 0.70 (0.46-0.98)* | 0.29  (-3.4)† | 0.42  (-2.4)† |
| mmu-miR-15b-5p | 26.3 ± 0.20 | 25.6 ± 0.12 | -0.50 ± 0.14 | -0.99 ± 0.06* | 1.40 (1.20-1.51)* | 2.4  (2.4)† | 3.2  (3.2)† |
| mmu-miR-23b-3p | 25.9 ± 0.23 | 25.7 ± 0.14 | -0.88 ± 0.11 | -0.81 ± 0.18 | 0.95 (0.64-1.22) | 0.36  (-2.8)† | 0.4  (-2.5)† |
| mmu-miR-34b-5p | 29.5 ± 0.37 | 30.5 ± 0.11* | 2.69 ± 0.28 | 3.99 ± 0.16* | 0.41 (0.29-0.57)* | 0.12  (-8.0)† | 0.15  (-6.7)† |
| mmu-miR-125b-5p | 26.7 ± 0.39 | 26.7 ± 0.48 | 0.11 ± 0.27 | -0.07 ± 0.41 | 1.13 (0.47-1.88) | 0.17  (-6.0)† | 0.25  (-4.0)† |
| mmu-miR-126-3p | 23.7 ± 0.46 | 24.2 ± 0.20 | -3.06 ± 0.34 | -2.37 ± 0.28 | 0.62 (0.31-1.03)* | 0.24  (-4.2)† | 0.25  (-3.9)† |
| mmu-miR-130b-3p | 34.6 ± 0.31 | 34.3 ± 0.18 | 7.96 ± 0.29 | 7.51 ± 0.26 | 1.37 (1.07-1.94) | 2.1  (2.1)† | 3.0  (3.0)† |
| mmu-miR-223-3p | 20.3 ± 0.09 | 19.6 ± 0.15* | -6.50 ± 0.12 | -6.95 ± 0.07* | 1.36 (1.24-1.61)* | 2.1  (2.1)† | 1.5  (1.5) |

MicroRNA expression was measured using Exiqon miRcury LNA miRNA assays and RT-qPCR in a separate set of samples (n=4-5 per group) independently generated from a different cohort of mice than those used in the gene profiling study. Expression of mmu-miR-103a-3p was used to normalize expression of candidate key miRs (dCt). Fold changes in miR expression in SP samples were calculated with the PBS group being equal to 1 (values in parenthesis denote the range of fold changes in individual SP samples compared with PBS). *p<0.05 compared with PBS group, t test; † FDR<0.05 in microarray gene profiling study.

**Supplementary Table 6:** Conserved DE and the numbers and percentages of predicted mRNA targets that are differentially expressed during pneumonia

| miRBase 21 | miR acc | RMA Fold-Change (SP vs. PBS) | LVS Fold-Change (SP vs. PBS) | Total targets | DOWN targets | UP targets | % Down | % Up |
| --- | --- | --- | --- | --- | --- | --- | --- | --- |
| mmu-miR-1224-5p | MIMAT0005460 | 5.1 | 4.3 | 144 | 15 | 7 | 10.42% | 4.86% |
| mmu-miR-188-5p | MIMAT0000217 | 4.7 | 3.5 | 177 | 22 | 13 | 12.4% | 7.3% |
| mmu-miR-139-5p | MIMAT0000656 | 3.5 | 3.0 | 391 | 67 | 31 | 17.14% | 7.93% |
| mmu-miR-15b-5p | MIMAT0000124 | 3.2 | 2.4 | 1171 | 182 | 75 | 15.5% | 6.4% |
| mmu-miR-721 | MIMAT0003515 | 2.8 | 2.2 | 800 | 151 | 66 | 18.9% | 8.3% |
| mmu-miR-18a-5p | MIMAT0000528 | 2.6 | 2.1 | 248 | 36 | 11 | 14.5% | 4.4% |
| mmu-miR-130b-3p | MIMAT0000387 | 3.0 | 2.1 | 800 | 151 | 66 | 18.88% | 8.25% |
| mmu-miR-379-5p | MIMAT0000743 | -2.3 | -2.0 | 102 | 12 | 6 | 11.8% | 5.9% |
| mmu-miR-331-3p | MIMAT0000571 | -2.2 | -2.6 | 260 | 39 | 19 | 15.0% | 7.3% |
| mmu-let-7c-5p | MIMAT0000523 | -2.6 | -2.6 | 1074 | 157 | 100 | 14.62% | 9.31% |
| mmu-miR-144-3p | MIMAT0000156 | -4.1 | -2.6 | 849 | 132 | 53 | 15.55% | 6.24% |
| mmu-miR-3473a | MIMAT0015645 | -2.9 | -2.6 | 355 | 66 | 15 | 18.6% | 4.2% |
| mmu-miR-582-5p | MIMAT0005291 | -2.6 | -2.7 | 586 | 96 | 35 | 16.4% | 6.0% |
| mmu-miR-23b-3p | MIMAT0000125 | -2.5 | -2.8 | 1062 | 160 | 86 | 15.1% | 8.1% |
| mmu-miR-183-5p | MIMAT0000212 | -3.6 | -2.8 | 421 | 78 | 27 | 18.5% | 6.4% |
| mmu-let-7d-5p | MIMAT0000383 | -2.1 | -3.0 | 1074 | 157 | 100 | 14.62% | 9.31% |
| mmu-miR-190b-5p | MIMAT0004852 | -5.1 | -3.0 | 183 | 32 | 9 | 17.5% | 4.9% |
| mmu-miR-181b-5p | MIMAT0000673 | -3.1 | -3.2 | 1121 | 182 | 74 | 16.2% | 6.6% |
| mmu-let-7b-5p | MIMAT0000522 | -2.3 | -3.2 | 1074 | 157 | 100 | 14.62% | 9.31% |
| mmu-miR-27b-3p | MIMAT0000126 | -2.6 | -3.2 | 1183 | 206 | 73 | 17.4% | 6.2% |
| mmu-let-7f-5p | MIMAT0000525 | -2.4 | -3.4 | 1074 | 157 | 100 | 14.62% | 9.31% |
| mmu-miR-150-5p | MIMAT0000160 | -4.3 | -3.4 | 326 | 53 | 22 | 16.26% | 6.75% |
| mmu-miR-30c-5p | MIMAT0000514 | -2.6 | -3.6 | 1354 | 206 | 123 | 15.2% | 9.1% |
| mmu-miR-218-5p | MIMAT0000663 | -3.8 | -3.6 | 958 | 152 | 41 | 15.9% | 4.3% |
| mmu-miR-34a-5p | MIMAT0000542 | -2.9 | -3.7 | 666 | 110 | 48 | 16.5% | 7.2% |
| mmu-miR-497a-5p | MIMAT0003453 | -3.8 | -3.8 | 1171 | 182 | 75 | 15.5% | 6.4% |
| mmu-miR-335-5p | MIMAT0000766 | -7.4 | -3.9 | 255 | 35 | 14 | 13.7% | 5.5% |
| mmu-miR-193b-3p | MIMAT0004859 | -5.4 | -3.9 | 233 | 41 | 14 | 17.6% | 6.0% |
| mmu-miR-152-3p | MIMAT0000162 | -3.8 | -3.9 | 645 | 106 | 51 | 16.4% | 7.9% |
| mmu-miR-378a-3p | MIMAT0003151 | -3.6 | -3.9 | 222 | 33 | 11 | 14.9% | 5.0% |
| mmu-miR-195a-5p | MIMAT0000225 | -2.9 | -4.2 | 1171 | 182 | 75 | 15.5% | 6.4% |
| mmu-miR-151-5p | MIMAT0004536 | -3.7 | -4.3 | 22 | 3 | 1 | 13.6% | 4.5% |
| mmu-miR-130a-3p | MIMAT0000141 | -4.1 | -4.4 | 800 | 151 | 66 | 18.88% | 8.25% |
| mmu-miR-99b-5p | MIMAT0000132 | -4.7 | -4.7 | 51 | 9 | 3 | 17.6% | 5.9% |
| mmu-miR-31-5p | MIMAT0000538 | -5.9 | -4.8 | 414 | 73 | 16 | 17.6% | 3.9% |
| mmu-miR-30a-5p | MIMAT0000128 | -4.1 | -5.0 | 1354 | 206 | 123 | 15.2% | 9.1% |
| mmu-miR-96-5p | MIMAT0000541 | -5.2 | -5.1 | 1061 | 195 | 69 | 18.4% | 6.5% |
| mmu-let-7e-5p | MIMAT0000524 | -3.8 | -5.2 | 1074 | 157 | 100 | 14.62% | 9.31% |
| mmu-miR-375-3p | MIMAT0000739 | -6.5 | -6.3 | 235 | 34 | 17 | 14.5% | 7.2% |
| mmu-miR-143-3p | MIMAT0000247 | -6.4 | -7.1 | 430 | 73 | 29 | 16.98% | 6.74% |
| mmu-miR-34b-5p | MIMAT0000382 | -6.7 | -8.0 | 666 | 110 | 48 | 16.5% | 7.2% |
| mmu-miR-34c-5p | MIMAT0000381 | -8.7 | -9.2 | 666 | 110 | 48 | 16.5% | 7.2% |
| mmu-miR-449a-5p | MIMAT0001542 | -9.8 | -10.1 | 666 | 110 | 48 | 16.5% | 7.2% |
| mmu-miR-224-5p | MIMAT0000671 | -6.9 | -30.8 | 359 | 56 | 22 | 15.6% | 6.1% |

Conserved DE miRs are sorted by fold change using LVS. miR accession numbers and names were based on miRBase 21. The total number of predicted mRNA targets according to TargetScan release 7.1 is listed in column 5. The total predicted targets were searched to identify the mRNAs that were differentially expressed in *S. pneumoniae* pneumonia. The numbers and percentages of DE predicted target mRNAs that were down or upregulated during pneumonia are shown. Upregulated miRs are in gray

**Supplementary Table 7:** Poorly conserved DE miRs and the numbers and percentages of predicted mRNA targets that are differentially expressed during pneumonia

| miRBase 21 | miR acc | RMA Fold-Change (SP vs. PBS) | LVS Fold-Change (SP vs. PBS) | Total targets | DOWN targets | UP targets | % DOWN targets | % UP targets |
| --- | --- | --- | --- | --- | --- | --- | --- | --- |
| mmu-miR-1896 | MIMAT0007873 | 21.7 | 14.9 | 7096 | 1048 | 483 | 14.77% | 6.81% |
| mmu-miR-1247-3p | MIMAT0014801 | 21.2 | 8.2 | 3490 | 556 | 210 | 15.93% | 6.02% |
| mmu-miR-3093-3p | MIMAT0014908 | 22.5 | 6.8 | 3665 | 569 | 243 | 15.53% | 6.63% |
| mmu-miR-3960 | MIMAT0019336 | 5.4 | 4.5 | 320 | 52 | 26 | 16.25% | 8.13% |
| mmu-miR-467a-3p | MIMAT0002108 | 6.6 | 4.2 | 5763 | 865 | 384 | 15.01% | 6.66% |
| mmu-miR-340-3p | MIMAT0000586 | 4.0 | 3.3 | 1070 | 154 | 95 | 14.39% | 8.88% |
| mmu-miR-3091-5p | MIMAT0014903 | 5.8 | 3.1 | 2741 | 420 | 163 | 15.32% | 5.95% |
| mmu-miR-483-5p | MIMAT0004782 | 5.2 | 3.0 | 962 | 168 | 68 | 17.46% | 7.07% |
| mmu-miR-135a-1-3p | MIMAT0004531 | 6.7 | 2.9 | 2388 | 369 | 148 | 15.45% | 6.20% |
| mmu-miR-1982-5p | MIMAT0009459 | 3.7 | 2.8 | 5891 | 884 | 412 | 15.01% | 6.99% |
| mmu-miR-3082-5p | MIMAT0014872 | 3.3 | 2.6 | 6401 | 930 | 467 | 14.53% | 7.30% |
| mmu-miR-2861 | MIMAT0013803 | 3.3 | 2.5 | 4162 | 629 | 296 | 15.11% | 7.11% |
| mmu-miR-139-3p | MIMAT0004662 | 4.4 | 2.5 | 3647 | 537 | 249 | 14.72% | 6.83% |
| mmu-miR-466i-5p | MIMAT0017325 | 2.8 | 2.3 | 6598 | 1018 | 437 | 15.43% | 6.62% |
| mmu-miR-680 | MIMAT0003457 | 3.0 | 2.3 | 3896 | 580 | 274 | 14.89% | 7.03% |
| mmu-miR-211-3p | MIMAT0017059 | 2.8 | 2.3 | 4630 | 683 | 323 | 14.75% | 6.98% |
| mmu-miR-5126 | MIMAT0020637 | 2.5 | 2.3 | 352 | 63 | 27 | 17.90% | 7.67% |
| mmu-miR-21a-3p | MIMAT0004628 | 3.3 | 2.2 | 5450 | 812 | 378 | 14.90% | 6.94% |
| mmu-miR-3077-5p | MIMAT0014862 | 5.7 | 2.2 | 514 | 83 | 37 | 16.15% | 7.20% |
| mmu-miR-574-5p | MIMAT0004893 | 2.7 | 2.1 | 5314 | 852 | 356 | 16.03% | 6.70% |
| mmu-miR-149-3p | MIMAT0016990 | 3.7 | 2.1 | 5908 | 921 | 393 | 15.59% | 6.65% |
| mmu-miR-3102-5p | MIMAT0014933 | 3.0 | 2.1 | 3770 | 569 | 254 | 15.09% | 6.74% |
| mmu-miR-1897-5p | MIMAT0007864 | 2.6 | 2.0 | 4617 | 724 | 302 | 15.68% | 6.54% |
| mmu-miR-297c-5p | MIMAT0004865 | 2.9 | 2.0 | 4633 | 697 | 324 | 15.04% | 6.99% |
| mmu-miR-24-2-5p | MIMAT0005440 | -2.1 | -2.2 | 3112 | 456 | 224 | 14.65% | 7.20% |
| mmu-miR-3075-5p | MIMAT0014858 | -2.2 | -2.2 | 4593 | 717 | 331 | 15.61% | 7.21% |
| mmu-miR-1a-3p | MIMAT0000123 | -3.7 | -2.3 | 729 | 127 | 52 | 17.42% | 7.13% |
| mmu-miR-101c | MIMAT0019349 | -2.1 | -2.4 | 3346 | 501 | 211 | 14.97% | 6.31% |
| mmu-miR-200a-5p | MIMAT0004619 | -3.6 | -2.4 | 3171 | 502 | 221 | 15.83% | 6.97% |
| mmu-miR-3472 | MIMAT0015643 | -2.2 | -2.5 | 2622 | 395 | 157 | 15.06% | 5.99% |
| mmu-miR-31-3p | MIMAT0004634 | -3.8 | -2.5 | 3178 | 510 | 216 | 16.05% | 6.80% |
| mmu-miR-28c | MIMAT0019339 | -2.6 | -2.5 | 4335 | 690 | 287 | 15.92% | 6.62% |
| mmu-miR-3470a | MIMAT0015640 | -2.5 | -2.7 | 5298 | 770 | 379 | 14.53% | 7.15% |
| mmu-miR-672-5p | MIMAT0003735 | -3.7 | -3.0 | 3703 | 557 | 253 | 15.04% | 6.83% |
| mmu-miR-200b-5p | MIMAT0004545 | -4.0 | -3.1 | 3171 | 502 | 221 | 15.83% | 6.97% |
| mmu-miR-145a-5p | MIMAT0000157 | -5.5 | -3.1 | 695 | 133 | 48 | 19.14% | 6.91% |
| mmu-miR-3473b | MIMAT0020367 | -2.6 | -3.2 | 6020 | 910 | 431 | 15.12% | 7.16% |
| mmu-miR-30c-2-3p | MIMAT0005438 | -4.3 | -3.2 | 5761 | 860 | 377 | 14.93% | 6.54% |
| mmu-miR-378b | MIMAT0019348 | -4.4 | -3.4 | 3560 | 539 | 220 | 15.14% | 6.18% |
| mmu-miR-125a-5p | MIMAT0000135 | -3.2 | -3.7 | 845 | 133 | 68 | 15.74% | 8.05% |
| mmu-miR-200c-3p | MIMAT0000657 | -3.4 | -3.8 | 1021 | 173 | 70 | 16.94% | 6.86% |
| mmu-miR-872-5p | MIMAT0004934 | -4.1 | -4.1 | 2479 | 368 | 170 | 14.84% | 6.86% |
| mmu-miR-690 | MIMAT0003469 | -3.2 | -4.1 | 4069 | 617 | 286 | 15.16% | 7.03% |
| mmu-miR-34c-3p | MIMAT0004580 | -6.3 | -5.0 | 3227 | 474 | 213 | 14.69% | 6.60% |
| mmu-miR-125b-5p | MIMAT0000136 | -4.0 | -6.0 | 845 | 133 | 68 | 15.74% | 8.05% |
| mmu-miR-200b-3p | MIMAT0000233 | -7.2 | -6.7 | 1021 | 173 | 70 | 16.94% | 6.86% |
| mmu-miR-429-3p | MIMAT0001537 | -6.2 | -6.9 | 1021 | 173 | 70 | 16.94% | 6.86% |
| mmu-miR-141-3p | MIMAT0000153 | -5.4 | -7.2 | 787 | 140 | 58 | 17.79% | 7.37% |
| mmu-miR-126a-5p | MIMAT0000137 | -6.9 | -7.8 | 4888 | 698 | 349 | 14.28% | 7.14% |
| mmu-miR-200a-3p | MIMAT0000519 | -7.9 | -8.5 | 787 | 140 | 58 | 17.79% | 7.37% |
| mmu-miR-34b-3p | MIMAT0004581 | -11.5 | -17.8 | 3227 | 474 | 213 | 14.69% | 6.60% |

miR accession numbers and names were based on miRBase 21. The total number of predicted mRNA targets according to TargetScan release 7.1 is listed in column 5. The total predicted targets were searched to identify the mRNAs that were differentially expressed in *S. pneumoniae* pneumonia. The numbers and percentages of DE predicted target mRNAs that were down or upregulated during pneumonia are shown. Upregulated miRs are in gray. Two poorly conserved DE miRs are not listed in TargetScan7.1: mmu-miR-203-3p and mmu-miR-126a-3p.

**Supplementary Table 8:** Conserved DE miRs that were upregulated during pneumonia and their anti-correlated predicted targets (mRNAs 2 fold or greater downregulated during pneumonia compared with PBS, FDR≤0.05).

| **Up regulated DE miR** | **Downregulated predicted target mRNAs** |
| --- | --- |
| mmu-miR-1224-5p | *Enah;Fign;Mlec;Nckap5;Nfib;Nrp1;Paqr4;Pkd1;Prickle2;Ptprf;Rbfox2;Rtn4rl1;Slc25a35;Slco2b1;Thrb* |
| mmu-miR-188-5p | *Akap2;Arhgap32;Baiap2l1;Cdc25b;Ddah2;Dlg5;Efnb2;Fam117b;Fzd8;Ggcx;Klf12;Magi1;Mgat3;Nbea;Pgm2l1;Plxdc2;Sh3bgrl2;Sox4;Spag6;Tmem50b;Zbtb20;Zfp185* |
| mmu-miR-139-5p | *Aff3;Agbl5;Arhgap5;Arrdc3;Atp2b2;C77370;Cacna2d1;Cecr2;Ckb;Cnst;Crim1;D630045J12Rik;Dcbld2;Dip2c;Dpy30;Dynll2;Ebf1;Elovl5;Enah;Ets1;Fam53b;Fbxl17;Fbxl7;Foxo1;Foxp2;Gabra3;Galnt7;Glcci1;Gpr56;H2afv;Hs2st1;Igfbp5;Kat6b;Kif13a;Ldhb;Lrp2;Map2;Meis2;Mfsd6;Mycbp;Nfia;Nfib;Nipal2;Pmp22;Ppargc1a;Prdm16;Ptpn4;Ptprf;Ptpru;Pvrl3;Rbms3;Rpgrip1l;Samd12;Sema6d;Slc16a12;Socs2;Sos2;Ssx2ip;Strbp;Tbx1;Tcf12;Tmpo;Trove2;Wdr47;Zbtb10;Zbtb20;Zfp532* |
| mmu-miR-15b-5p | *1700021K19Rik;1700030J22Rik;AA986860;Adamts5;Ago1;Ahcyl2;Akap11;Amotl2;Arhgap32;Arhgap35;Arhgap5;Bace1;Bach2;Bcl7a;Bcl9l;Bmx;Cacna2d1;Cacnb1;Cadm1;Card10;Cbfa2t3;Cbx5;Cbx6;Ccdc19;Ccnd1;Cd2ap;Cdc25b;Cdon;Chd6;Chordc1;Clock;Cluh;Cnksr2;Cnnm2;Cobll1;Crim1;Ctdspl;Cyb561;Cyp26b1;Dgkg;Dixdc1;Dll4;Dnajb2;Dock1;Dolpp1;Dynll2;Efnb2;Elmod1;Enah;Epha1;Epha7;Esrp2;Eya1;Fam120c;Fam135a;Fam161a;Fam171a1;Fam60a;Fam81a;Fasn;Fbln5;Fbxo21;Fbxo8;Fermt2;Fgf18;Foxp2;Fry;Gabre;Garem;Ghr;Glp1r;Gm166;Helz;Ints3;Kank1;Kdr;Kif21a;Kif23;Krt80;Leprel1;Llgl2;Lphn1;Lrig1;Lrp2;Lrp6;Ltbp2;Lurap1l;Map7;Mical3;Mink1;Mmd;Mob3b;Myadm;Myb;Mylk;Myo10;Nebl;Nedd4l;Nkd1;Nol3;Nol4l;Nos1ap;Nrarp;Nrn1;Nup210;Nynrin;Ogt;Pam;Pcdh1;Pdcd4;Phka1;Phlda3;Plekha5;Plekhh1;Plscr4;Plxna4;Pmm1;Pnpla6;Ppm1l;Prkca;Prkg1;Ptch1;Ptpn14;Ptpn3;Ptpn4;Ptprd;Ptprm;Pvrl1;Rab3d;Rarb;Rasef;Reck;Rfx3;Rnf125;Rtn4rl1;Sel1l3;Sema3a;Sema3b;Sema3d;Sema6d;Sesn1;Sftpb;Sh3bgrl2;Sh3pxd2a;Sil1;Slc12a2;Slc22a17;Slc22a23;Slc25a35;Slc2a3;Slc39a10;Slc4a4;Slc9a6;Smad5;Sms;Sos2;Sox6;Stk33;Stox2;Stradb;Strbp;Syde2;Synrg;Tcte1;Tead1;Tmem245;Tmem38a;Trim2;Trim68;Tspan1;Tspan7;Ttc21b;Tuba1a;Unc119b;Usp53;Vav2;Vstm2a;Wdr47;Zbtb10;Zbtb20;Zfp362;Zfp532* |
| mmu-miR-721 | *2700081O15Rik;9430020K01Rik;Abhd3;Acer2;Acvr1;Aff3;Ago1;Agpat3;Akap11;Ar;Arfip1;Arhgap21;Arhgap35;Arhgef12;Arrdc3;Atp2b2;Atrn;Bach2;Bmpr1b;Btbd3;Casd1;Ccdc85a;Ccna2;Cdc14a;Cds1;Chrm2;Chst1;Clock;Cluh;Csrp2;Dcbld2;Dgke;Dicer1;Dip2c;Dlc1;Dnal1;Dpysl2;Dynll2;E2f2;Efnb2;Elmod1;Enah;Endod1;Eogt;Ephb4;Fam13a;Fam53b;Fam73a;Fam84a;Fermt2;Fibin;Fign;Fsd1l;Fzd6;Garem;Grb10;Habp4;Hecw2;Hlf;Htr1b;Impdh1;Ipcef1;Lclat1;Ldlrad4;Lgalsl;Lmln;Lrig1;Lrp2;Lrp4;Lrp6;Map3k12;Map7;Mapre3;Mfap3l;Mfsd6;Mid1ip1;Mlec;Mmgt1;Mob3b;Mtcl1;Myb;Myh14;Myo10;Nbea;Nckap5;Nfia;Nfib;Nfic;Npepl1;Npnt;Nr3c2;Nrarp;Nrp1;Osbpl6;Pgm2l1;Plcb1;Plekhg5;Pparg;Ppp1r9a;Prkg1;Psd3;Ptgfrn;Ptpn14;Ptprg;Ptprm;Pvrl3;Pxdn;Qser1;Rarb;Rasgef1a;Rbfox2;Sel1l3;Sh3d19;Shank2;Slc12a7;Slc39a10;Smad5;Socs2;Sos2;Sox4;Spire1;Stard13;Stox2;Sulf1;Tacc2;Tanc2;Tfcp2l1;Tgfb2;Tle1;Tmem159;Tmem50b;Tom1l2;Trim2;Trim3;Trim37;Trove2;Tshz1;Ttc9;Ulk2;Vsig10;Wdr47;Wdr54;Wnt2b;Zadh2;Zbtb20;Zbtb4;Zcchc14;Zfp113;Zfp652;Zfp704;Zmat3* |
| mmu-miR-18a-5p | *Add3;Ank3;Ar;Arhgap5;Atp8a1;Camk2n1;Crim1;Ctdspl;Dip2c;Endod1;Epb4.1l1;Epha7;Epn3;Fam166b;Fbxl7;Fgf1;Inadl;Mylk;Nrep;Pdzd2;Psd3;Ptgfrn;Ptp4a3;Ptpn13;Rasal2;Sh3bp4;Sorbs2;Sox6;Srebf1;Tie1;Tmem212;Trib2;Trim2;Zbtb20;Zbtb4;Zfp704* |
| mmu-miR-130b-3p | *2700081O15Rik;9430020K01Rik;Abhd3;Acer2;Acvr1;Aff3;Ago1;Agpat3;Akap11;Ar;Arfip1;Arhgap21;Arhgap35;Arhgef12;Arrdc3;Atp2b2;Atrn;Bach2;Bmpr1b;Btbd3;Casd1;Ccdc85a;Ccna2;Cdc14a;Cds1;Chrm2;Chst1;Clock;Cluh;Csrp2;Dcbld2;Dgke;Dicer1;Dip2c;Dlc1;Dnal1;Dpysl2;Dynll2;E2f2;Efnb2;Elmod1;Enah;Endod1;Eogt;Ephb4;Fam13a;Fam53b;Fam73a;Fam84a;Fermt2;Fibin;Fign;Fsd1l;Fzd6;Garem;Grb10;Habp4;Hecw2;Hlf;Htr1b;Impdh1;Ipcef1;Lclat1;Ldlrad4;Lgalsl;Lmln;Lrig1;Lrp2;Lrp4;Lrp6;Map3k12;Map7;Mapre3;Mfap3l;Mfsd6;Mid1ip1;Mlec;Mmgt1;Mob3b;Mtcl1;Myb;Myh14;Myo10;Nbea;Nckap5;Nfia;Nfib;Nfic;Npepl1;Npnt;Nr3c2;Nrarp;Nrp1;Osbpl6;Pgm2l1;Plcb1;Plekhg5;Pparg;Ppp1r9a;Prkg1;Psd3;Ptgfrn;Ptpn14;Ptprg;Ptprm;Pvrl3;Pxdn;Qser1;Rarb;Rasgef1a;Rbfox2;Sel1l3;Sh3d19;Shank2;Slc12a7;Slc39a10;Smad5;Socs2;Sos2;Sox4;Spire1;Stard13;Stox2;Sulf1;Tacc2;Tanc2;Tfcp2l1;Tgfb2;Tle1;Tmem159;Tmem50b;Tom1l2;Trim2;Trim3;Trim37;Trove2;Tshz1;Ttc9;Ulk2;Vsig10;Wdr47;Wdr54;Wnt2b;Zadh2;Zbtb20;Zbtb4;Zcchc14;Zfp113;Zfp652;Zfp704;Zmat3* |

**Supplementary Table 9:** Conserved downregulated DE miRs and their anti-correlated predicted targets

| **Down regulated DE miR** | **Upregulated predicted target mRNAs** |
| --- | --- |
| mmu-miR-224-5p | *2010111I01Rik;Adam17;Apod;Dnase1l3;Dot1l;Dr1;Dusp16;Egr2;Fap;Gch1;Igf2r;Il1rn;Jdp2;Lcor;Mafg;Pcgf5;Ptx3;Serpine1;Sh3bp5;Tank;Wtap;Zcchc4* |
| mmu-miR-449a-5p | *AW549877;Abr;Acbd3;Acsl1;Atxn7l3;Cdc42ep2;Cdk6;Coro1c;Dgkz;Dvl2;Eea1;Elf4;Ergic1;Esrra;Fam167a;Fam46a;Fam76a;Fbxo30;Fndc3a;Foxn2;Itk;Map2k1;March5;Mgat4a;Nampt;Nceh1;Osgin2;P2ry2;Pid1;Pip5k1a;Ppp4r2;Ptges;Ralgds;Rgs1;Rnf213;Rnf34;Rps6ka4;Rragc;Serpine1;Slc31a2;Slco3a1;Smox;Sp2;St6galnac4;Tmem251;Vcpip1;Ywhag;Zfp281* |
| mmu-miR-34c-5p | *AW549877;Abr;Acbd3;Acsl1;Atxn7l3;Cdc42ep2;Cdk6;Coro1c;Dgkz;Dvl2;Eea1;Elf4;Ergic1;Esrra;Fam167a;Fam46a;Fam76a;Fbxo30;Fndc3a;Foxn2;Itk;Map2k1;March5;Mgat4a;Nampt;Nceh1;Osgin2;P2ry2;Pid1;Pip5k1a;Ppp4r2;Ptges;Ralgds;Rgs1;Rnf213;Rnf34;Rps6ka4;Rragc;Serpine1;Slc31a2;Slco3a1;Smox;Sp2;St6galnac4;Tmem251;Vcpip1;Ywhag;Zfp281* |
| mmu-miR-34b-5p | *AW549877;Abr;Acbd3;Acsl1;Atxn7l3;Cdc42ep2;Cdk6;Coro1c;Dgkz;Dvl2;Eea1;Elf4;Ergic1;Esrra;Fam167a;Fam46a;Fam76a;Fbxo30;Fndc3a;Foxn2;Itk;Map2k1;March5;Mgat4a;Nampt;Nceh1;Osgin2;P2ry2;Pid1;Pip5k1a;Ppp4r2;Ptges;Ralgds;Rgs1;Rnf213;Rnf34;Rps6ka4;Rragc;Serpine1;Slc31a2;Slco3a1;Smox;Sp2;St6galnac4;Tmem251;Vcpip1;Ywhag;Zfp281* |
| mmu-miR-143-3p | *A1cf;Abl2;Acbd3;Aff1;Atp10a;Atp6v1a;BC005537;Brd2;Cox17;Creb5;Dcp2;Filip1l;Gns;Hk2;Hspb7;Igf2r;Larp1;Maf;Mapk7;Marcks;Milr1;Naa25;Oser1;Phf11a;Phf11d;Plagl2;Slc39a14;Sp2;Txlnb* |
| mmu-miR-375-3p | *Cdk5r1;Commd8;Coro2a;Cpeb4;Frrs1;Jak2;Mgat4a;Pde4d;Rasd1;Rbpj;Rgs16;Ski;Spag9;Tbc1d9;Tsc1;Zfp281;Zfyve26* |
| mmu-let-7e-5p | *1700017B05Rik;AI606181;AW549877;Abcc5;Abl2;Ankrd49;Arrdc4;Bcl2l1;Ccdc71l;Ccnf;Cd200r1;Cdk6;Chd7;Cish;Clp1;Col15a1;Cpeb4;Cyb561d1;Dtx2;Dtx4;Dusp16;Dyrk1a;Edn1;Eea1;Efhd2;Egr3;Elf4;Ergic1;Ero1l;Etnk1;Fam214b;Fbxo30;Fndc3a;Fnip1;Fnip2;Gas7;Gatm;Gbp7;Gm13889;Gpatch3;Gpr157;Gramd1c;Hic2;Hk2;Hmga1;Iglon5;Ikbke;Il10;Kcng3;Kdm3a;Klhl6;Klkb1;Lcor;Lonrf3;Mab21l3;Map4k4;Mapk6;Mgat4a;Msr1;Nab1;Nceh1;Nhlrc3;Nlrc5;Olr1;Osbpl3;Pacs2;Pias4;Pik3ip1;Plagl2;Plcxd2;Pmaip1;Pqlc2;Prdm1;Ptafr;Rab15;Ranbp2;Rbpj;Rffl;Rgs16;Rufy3;Skil;Slc20a1;Slc36a1;Slc8b1;Smim3;Socs1;Stxbp5;Tbkbp1;Tet3;Tgfbr1;Tnfaip3;Tnfrsf1b;Tor1aip2;Trib1;Tsc1;Vcpip1;Xkr8;Zfp281;Zfyve26;Zswim4* |
| mmu-miR-96-5p | *Abca1;Arel1;Arid5b;Atp6v1c1;B3gnt2;Basp1;Bcl2l11;Cdyl2;Clvs1;Coro1c;Cpeb4;Ctdp1;Cyb561d1;D17Wsu92e;Denr;Eea1;Egr3;Ehd1;Ell2;Elmo1;F13a1;Fhl3;Fmr1;Furin;Homer1;Hspa13;Iqsec1;Lcor;Lpp;Maf;Map2k1;Med12l;Mitf;Mterfd3;N4bp1;Nampt;Nhlrc3;Nr4a3;Pcgf5;Plekhm1;Pou2f2;Ppp4r2;Prdm1;Rabgap1;Rap2b;Rev1;Rhob;Sh3bp5;Slc1a2;Slc43a2;Slc7a8;Slco3a1;Slmo2;Smcr8;Snf8;Sowahc;Spen;Sppl2a;Stxbp5;Tes;Tgfbr1;Tmem189;Tmem86a;Tns3;Vps26a;Ywhag;Zbtb7a;Zhx2;Znrf1* |
| mmu-miR-30a-5p | *3110043O21Rik;5031414D18Rik;A230046K03Rik;AI606181;Abl2;Acap2;Acp2;Adamts6;Ankrd12;Arid5b;Ascc3;Atp6v1c1;B4galt5;Bcl2l11;Bcor;Bdp1;Ccdc71l;Ccnf;Cdyl2;Chd7;Clcf1;Cmpk2;Cpeb4;Dcp2;Ddit4;Dgkq;Dgkz;Dnajc13;Eaf1;Eea1;Ell2;Elmo1;Esco1;Fam46a;Fap;Fndc3a;Fnip2;Gas7;Gatm;Gmeb1;Gna13;Gnpda1;Gpr157;Gtf2h1;Hic2;Ier5;Igf2r;Il1a;Inhba;Irf1;Itga5;Jdp2;Kdm3a;Kif1c;Klf10;Larp1;Lcor;Lonrf3;Lpp;Lrrc8d;Lrrfip2;Lsmem1;Maf;Mafg;Map4k4;Marcks;Med12l;Myo5a;N4bp2;Naa25;Nmi;Orai2;Osbpl8;Pcgf5;Pde4d;Peli1;Plagl2;Plcxd2;Plekho2;Plk2;Prdm1;Ptafr;Rab15;Rab22a;Rab32;Rap2c;Rasd1;Rev1;Rffl;Rgcc;Rhob;Rps6ka2;Rrad;Serpine1;Sik3;Sirt1;Skil;Slc1a2;Slc36a1;Slc38a7;Slc7a11;Slc9a8;Snx8;Socs1;Socs3;Sp4;Spen;Stx2;Stxbp5;Tcp11l1;Tnfrsf8;Traf6;Trpm7;Txlnb;Ube2f;Usp2;Ywhag;Zbtb7a;Zeb2;Zfp281;Zfp507;Zfyve26;Znrf1* |
| mmu-miR-31-5p | *B3gnt2;C030046E11Rik;Chd7;Clcf1;Egln3;Ehd1;Homer1;Itga5;Lpp;Mafg;Mapkapk2;Slc1a2;Slc39a14;Slc43a2;Zbtb21;Zc3h12c* |
| mmu-miR-99b-5p | *Cdyl2;Trib1;Zbtb7a* |
| mmu-miR-130a-3p | *Abca1;Acbd3;Acsl1;Ankrd12;B4galt5;Bcl2l11;Blcap;Ccdc126;Ccrn4l;Cd69;Cpeb4;Creb5;Csf1;Cyld;Dcp2;Ereg;Esr1;Etnk1;Fmr1;Gca;Hivep1;Inhba;Irf1;Lcor;Lonrf3;Lrp8;Lsmem1;Lyl1;M6pr;Maf;Map4;Map4k4;Med12l;Mllt6;N4bp1;Naa25;Pcgf5;Peli1;Pik3ip1;Rap2c;Rasd1;Rbbp8;Rpa2;Rybp;Sh3bp5;Snip1;Spen;Sphk2;Spty2d1;Stx6;Tdrd7;Tet3;Tgfbr1;Tgif1;Tmem55b;Tnf;Tnfrsf1b;Tor1aip2;Tsc1;Vps37b;Zbtb7a;Zc3h12c;Zeb2;Zfp131;Zfyve26;Znrf1* |
| mmu-miR-151-5p | *N4bp1* |
| mmu-miR-195a-5p | *5031414D18Rik;AW549877;Abl2;Acsl1;Arel1;Atp6v1a;Atxn7l3;B4galt1;C030046E11Rik;Cacul1;Ccdc59;Ccnyl1;Cdc42ep2;Cdca4;Cdk5r1;Ciapin1;Csf1;Dcp2;Dtx4;Eif4e;Ell2;Esrra;Etnk1;Fam133b;Fkbp5;Furin;Gm13889;Gna13;Hk2;Hmga1;Igf2r;Il7r;Irak2;Kcnab1;Kcnn4;Kif1c;Lcor;Lonrf3;Lpp;Lrrfip2;Map2k1;Map4;Mgat4a;Mllt6;N4bp1;Naa25;Pcgf5;Pid1;Plcxd2;Rap2c;Rbbp6;Rfx5;Siah1a;Ski;Slc25a22;Slc25a37;Slc36a1;Smad3;Sowahc;Spen;Stxbp3a;Suz12;Tet3;Tmem55b;Tor1aip2;Traf3;Tsc1;Ubap1;Usp25;Vps18;Vps37c;Wsb2;Zbtb43;Zfp622;Znrf2* |
| mmu-miR-378a-3p | *Arf2;Dyrk1a;Fkbp5;Kbtbd7;Mafg;Pde1b;Plagl2;Rffl;Tnfrsf1b;Trim56;Zc3h12c* |
| mmu-miR-152-3p | *2010111I01Rik;Abca1;Abl2;Arel1;Atp6ap2;B4galt1;B4galt5;BC005537;Bcl2l11;Ccnf;Cdk5r1;Chd7;Cpeb4;Csf1;Dcp2;Denr;Dgkq;Dnmt1;Dyrk1a;Egr2;Elmo1;Esco1;Esr1;Etnk1;Fmr1;Gtf2h1;Hivep1;Itga5;Lcor;Lipa;Maf;Med12l;Mgat4a;Mitf;Mllt6;Nceh1;Nt5c3;Patl1;Rybp;Serpine1;Slc7a11;Spty2d1;Stxbp5;Tcp11l1;Tgfbr1;Tnfrsf1b;Ubash3b;Zbtb7a;Zc3h12c;Zfyve26;Znrf1* |
| mmu-miR-193b-3p | *Abca1;Acp2;Cd38;Crtc2;Dyrk1a;Kbtbd7;Mmp14;Mmp19;Plagl2;Plau;Psd4;Rfx5;Siah1a;Slc43a2* |
| mmu-miR-335-5p | *Ehd1;Elf4;F13a1;Fmr1;Lcor;Naa25;Nr4a3;Prss46;Rbm7;Sh3bp5;Snip1;Tpst1;Trim26;Zeb2* |
| mmu-miR-497a-5p | *5031414D18Rik;AW549877;Abl2;Acsl1;Arel1;Atp6v1a;Atxn7l3;B4galt1;C030046E11Rik;Cacul1;Ccdc59;Ccnyl1;Cdc42ep2;Cdca4;Cdk5r1;Ciapin1;Csf1;Dcp2;Dtx4;Eif4e;Ell2;Esrra;Etnk1;Fam133b;Fkbp5;Furin;Gm13889;Gna13;Hk2;Hmga1;Igf2r;Il7r;Irak2;Kcnab1;Kcnn4;Kif1c;Lcor;Lonrf3;Lpp;Lrrfip2;Map2k1;Map4;Mgat4a;Mllt6;N4bp1;Naa25;Pcgf5;Pid1;Plcxd2;Rap2c;Rbbp6;Rfx5;Siah1a;Ski;Slc25a22;Slc25a37;Slc36a1;Smad3;Sowahc;Spen;Stxbp3a;Suz12;Tet3;Tmem55b;Tor1aip2;Traf3;Tsc1;Ubap1;Usp25;Vps18;Vps37c;Wsb2;Zbtb43;Zfp622;Znrf2* |
| mmu-miR-34a-5p | *AW549877;Abr;Acbd3;Acsl1;Atxn7l3;Cdc42ep2;Cdk6;Coro1c;Dgkz;Dvl2;Eea1;Elf4;Ergic1;Esrra;Fam167a;Fam46a;Fam76a;Fbxo30;Fndc3a;Foxn2;Itk;Map2k1;March5;Mgat4a;Nampt;Nceh1;Osgin2;P2ry2;Pid1;Pip5k1a;Ppp4r2;Ptges;Ralgds;Rgs1;Rnf213;Rnf34;Rps6ka4;Rragc;Serpine1;Slc31a2;Slco3a1;Smox;Sp2;St6galnac4;Tmem251;Vcpip1;Ywhag;Zfp281* |
| mmu-miR-218-5p | *AI606181;Abl2;Acsl1;BC005537;Cacul1;Dnajc13;Ell2;Elmo1;Hic2;Hivep1;Itpkc;Jdp2;Kcng3;Lpp;Lyl1;Mafg;Marcks;Med12l;Mitf;Mtmr7;Nab1;Plcxd2;Plekhf2;Plekhg1;Plgrkt;Ppp4r2;Rffl;Rhoq;Rnf19b;Rybp;Sh3gl1;Ski;Skil;Slc1a2;Socs3;Stxbp5;Tnc;Zeb2;Zfyve26;Zmiz1;Znfx1* |
| mmu-miR-30c-5p | *3110043O21Rik;5031414D18Rik;A230046K03Rik;AI606181;Abl2;Acap2;Acp2;Adamts6;Ankrd12;Arid5b;Ascc3;Atp6v1c1;B4galt5;Bcl2l11;Bcor;Bdp1;Ccdc71l;Ccnf;Cdyl2;Chd7;Clcf1;Cmpk2;Cpeb4;Dcp2;Ddit4;Dgkq;Dgkz;Dnajc13;Eaf1;Eea1;Ell2;Elmo1;Esco1;Fam46a;Fap;Fndc3a;Fnip2;Gas7;Gatm;Gmeb1;Gna13;Gnpda1;Gpr157;Gtf2h1;Hic2;Ier5;Igf2r;Il1a;Inhba;Irf1;Itga5;Jdp2;Kdm3a;Kif1c;Klf10;Larp1;Lcor;Lonrf3;Lpp;Lrrc8d;Lrrfip2;Lsmem1;Maf;Mafg;Map4k4;Marcks;Med12l;Myo5a;N4bp2;Naa25;Nmi;Orai2;Osbpl8;Pcgf5;Pde4d;Peli1;Plagl2;Plcxd2;Plekho2;Plk2;Prdm1;Ptafr;Rab15;Rab22a;Rab32;Rap2c;Rasd1;Rev1;Rffl;Rgcc;Rhob;Rps6ka2;Rrad;Serpine1;Sik3;Sirt1;Skil;Slc1a2;Slc36a1;Slc38a7;Slc7a11;Slc9a8;Snx8;Socs1;Socs3;Sp4;Spen;Stx2;Stxbp5;Tcp11l1;Tnfrsf8;Traf6;Trpm7;Txlnb;Ube2f;Usp2;Ywhag;Zbtb7a;Zeb2;Zfp281;Zfp507;Zfyve26;Znrf1* |
| mmu-miR-150-5p | *3110043O21Rik;Acbd3;Angel2;Basp1;Commd8;Cxcl1;Cyb561d1;Dyrk1a;Egr2;Eif4e;Fkbp5;Hilpda;Irf2bp2;Mmp14;Pik3ap1;Prdm2;Rab9;Rnf34;Slc1a2;Tet3;Txlnb;Zfp189* |
| mmu-let-7f-5p | *1700017B05Rik;AI606181;AW549877;Abcc5;Abl2;Ankrd49;Arrdc4;Bcl2l1;Ccdc71l;Ccnf;Cd200r1;Cdk6;Chd7;Cish;Clp1;Col15a1;Cpeb4;Cyb561d1;Dtx2;Dtx4;Dusp16;Dyrk1a;Edn1;Eea1;Efhd2;Egr3;Elf4;Ergic1;Ero1l;Etnk1;Fam214b;Fbxo30;Fndc3a;Fnip1;Fnip2;Gas7;Gatm;Gbp7;Gm13889;Gpatch3;Gpr157;Gramd1c;Hic2;Hk2;Hmga1;Iglon5;Ikbke;Il10;Kcng3;Kdm3a;Klhl6;Klkb1;Lcor;Lonrf3;Mab21l3;Map4k4;Mapk6;Mgat4a;Msr1;Nab1;Nceh1;Nhlrc3;Nlrc5;Olr1;Osbpl3;Pacs2;Pias4;Pik3ip1;Plagl2;Plcxd2;Pmaip1;Pqlc2;Prdm1;Ptafr;Rab15;Ranbp2;Rbpj;Rffl;Rgs16;Rufy3;Skil;Slc20a1;Slc36a1;Slc8b1;Smim3;Socs1;Stxbp5;Tbkbp1;Tet3;Tgfbr1;Tnfaip3;Tnfrsf1b;Tor1aip2;Trib1;Tsc1;Vcpip1;Xkr8;Zfp281;Zfyve26;Zswim4* |
| mmu-miR-27b-3p | *A230046K03Rik;AI606181;Abca1;Abl2;Acsl1;Adamts6;Adora2b;Arid5b;Arrdc4;Bcor;Bdp1;Blm;Ccnyl1;Cdk5r1;Cdk6;Chd7;Cipc;Commd8;Cpeb4;Csf1;Cxcl11;Dcp2;Dnajc13;Dot1l;Dtx4;Elf4;Ell2;Elmo1;Fam133b;Fndc3a;Gns;Gramd1c;Ier3;Iglon5;Itga5;Itpkc;Kdm3a;Lcor;Marcks;Med12l;Mitf;Mmp13;N4bp1;Nek6;Papd7;Plagl2;Plcxd2;Plk2;Rab20;Rap2b;Rbbp6;Rffl;Rgs1;Rnf185;Rxra;Rybp;Slc1a2;Slc37a2;Slc7a11;Spata13;Spty2d1;Stx6;Suz12;Tbkbp1;Tet3;Tgfbr1;Tgif1;Traf3;Trim34a;Tsc1;Ube2f;Wsb1;Zeb2* |
| mmu-let-7b-5p | *1700017B05Rik;AI606181;AW549877;Abcc5;Abl2;Ankrd49;Arrdc4;Bcl2l1;Ccdc71l;Ccnf;Cd200r1;Cdk6;Chd7;Cish;Clp1;Col15a1;Cpeb4;Cyb561d1;Dtx2;Dtx4;Dusp16;Dyrk1a;Edn1;Eea1;Efhd2;Egr3;Elf4;Ergic1;Ero1l;Etnk1;Fam214b;Fbxo30;Fndc3a;Fnip1;Fnip2;Gas7;Gatm;Gbp7;Gm13889;Gpatch3;Gpr157;Gramd1c;Hic2;Hk2;Hmga1;Iglon5;Ikbke;Il10;Kcng3;Kdm3a;Klhl6;Klkb1;Lcor;Lonrf3;Mab21l3;Map4k4;Mapk6;Mgat4a;Msr1;Nab1;Nceh1;Nhlrc3;Nlrc5;Olr1;Osbpl3;Pacs2;Pias4;Pik3ip1;Plagl2;Plcxd2;Pmaip1;Pqlc2;Prdm1;Ptafr;Rab15;Ranbp2;Rbpj;Rffl;Rgs16;Rufy3;Skil;Slc20a1;Slc36a1;Slc8b1;Smim3;Socs1;Stxbp5;Tbkbp1;Tet3;Tgfbr1;Tnfaip3;Tnfrsf1b;Tor1aip2;Trib1;Tsc1;Vcpip1;Xkr8;Zfp281;Zfyve26;Zswim4* |
| mmu-miR-181b-5p | *2810474O19Rik;Acap2;Acsl1;Adamts6;Adm;Aff1;Atp6v1a;B4galt1;BC005537;Bcl2l11;Bhlhe40;Ccl7;Cd69;Chd7;Cmpk2;Cpeb4;D16Ertd472e;Dcp2;Ddit4;Dnajc13;Dock10;Egr3;Esr1;Etnk1;Eya3;Fmnl2;Fmr1;Fndc3a;Fnip2;Gas7;Hexim1;Hic2;Homer1;Il1a;Inhba;Jdp2;Lcor;Lif;Lpp;Lyrm1;Map4k4;Marcks;Med12l;Mitf;Mmp14;Nab1;Nr4a3;Osbpl3;Osbpl8;Pacs2;Phlda1;Plau;Ppp4r2;Rnf34;Serpine1;Sik3;Sirt1;Slc25a37;Spag9;Spty2d1;Tgfbr1;Tnf;Ttc39b;Vcan;Whamm;Wsb1;Ywhag;Zbtb21;Zbtb43;Zbtb7a;Zeb2;Zfp622;Zfp655;Znrf2* |
| mmu-miR-190b-5p | *C030046E11Rik;Ccdc122;Chd7;Cyb561d1;Kcng3;Myo5a;Rap2b;Tank;Wsb1* |
| mmu-let-7d-5p | *1700017B05Rik;AI606181;AW549877;Abcc5;Abl2;Ankrd49;Arrdc4;Bcl2l1;Ccdc71l;Ccnf;Cd200r1;Cdk6;Chd7;Cish;Clp1;Col15a1;Cpeb4;Cyb561d1;Dtx2;Dtx4;Dusp16;Dyrk1a;Edn1;Eea1;Efhd2;Egr3;Elf4;Ergic1;Ero1l;Etnk1;Fam214b;Fbxo30;Fndc3a;Fnip1;Fnip2;Gas7;Gatm;Gbp7;Gm13889;Gpatch3;Gpr157;Gramd1c;Hic2;Hk2;Hmga1;Iglon5;Ikbke;Il10;Kcng3;Kdm3a;Klhl6;Klkb1;Lcor;Lonrf3;Mab21l3;Map4k4;Mapk6;Mgat4a;Msr1;Nab1;Nceh1;Nhlrc3;Nlrc5;Olr1;Osbpl3;Pacs2;Pias4;Pik3ip1;Plagl2;Plcxd2;Pmaip1;Pqlc2;Prdm1;Ptafr;Rab15;Ranbp2;Rbpj;Rffl;Rgs16;Rufy3;Skil;Slc20a1;Slc36a1;Slc8b1;Smim3;Socs1;Stxbp5;Tbkbp1;Tet3;Tgfbr1;Tnfaip3;Tnfrsf1b;Tor1aip2;Trib1;Tsc1;Vcpip1;Xkr8;Zfp281;Zfyve26;Zswim4* |
| mmu-miR-183-5p | *Abca1;B3gnt2;Cdk5r1;Cdyl2;Ctsb;Eea1;Foxn2;Gbp7;Lhfpl2;Mapkapk2;Med12l;Morc3;Nr4a3;Osbpl8;Pde4d;Plagl2;Rap2c;Rhob;Sik3;Skil;Sp2;Sppl2a;Tox4;Tsc1;Usp2;Zeb2;Zfyve26* |
| mmu-miR-23b-3p | *A1cf;Abl2;Adamts6;Aff1;Arel1;Arhgap27;Atp6v1e1;Atxn7l3;Azi2;BC005537;Blcap;Cacul1;Casp7;Ccl7;Ccnl2;Cdk5r1;Chd7;Cipc;Cpeb4;Eea1;Egr3;Elf4;Ell2;Enc1;Ero1l;Esco1;Etnk1;Exoc3l4;Fam46a;Fas;Fbxo30;Fmr1;Fnip1;Fnip2;Gbp7;Gnpda1;Hexim1;Irf1;Lhfpl2;Lonrf3;Lpp;M6pr;Maf;Map4k4;Marcks;Marcksl1;Mb21d1;Mcfd2;Med12l;Mitf;Msr1;Mtm1;Mtmr7;Nab1;Nampt;Ndfip2;Nek6;Osbpl8;Plekhf2;Prdm1;Ptger4;Ptk2b;Rap2b;Rybp;Safb2;Sirt1;Slco3a1;Smad3;Tal2;Tbc1d9;Tet3;Tgif1;Tmem38b;Tnfaip3;Tpst1;Traf1;Trib1;Txnrd1;Vcan;Wdfy1;Whamm;Ywhag;Zbtb43;Zc3h12c;Zfp655;Znrf2* |
| mmu-miR-582-5p | *Adamts6;Ankrd12;Ascc3;Ccdc71l;Cdca4;Cdk6;Chd7;Chmp4b;D16Ertd472e;Dcp2;Dtnbp1;Eea1;Eif4e;Ero1l;Filip1l;Fmr1;Fnbp4;Igf2r;Jak2;Kcng3;Mitf;Naa25;Nek6;Pde4d;Phlda1;Plcxd2;Pou2f2;Rev1;Rufy3;Sp4;Spag9;Stxbp3a;Tet3;Ubash3b;Vps4b* |
| mmu-miR-3473a | *Btnl2;D17Wsu92e;Enc1;Ergic1;Hic2;Igf2r;Lcor;M6pr;Manbal;N4bp1;Phf7;Sh3bp5;Slc39a14;Tgfb1;Traf3* |
| mmu-miR-144-3p | *A830080D01Rik;Abca1;Acbd3;Als2;Ankrd12;Arfgef2;Arid5b;BC005537;Bcl2l11;Dr1;Dtnbp1;Dyrk1a;Eea1;Ell2;Ero1l;Esco1;Filip1l;Fmr1;Fndc3a;Hccs;Hnrnph2;Hvcn1;Iqsec1;Jdp2;Lcor;Mafk;Map3k8;Mapk6;Marcks;Med12l;Mitf;Mmp19;Pcgf5;Pde4d;Phlda1;Pip5k1c;Plekhg1;Rap2b;Rap2c;Rbbp6;Skil;Slc7a11;Sp4;Tet3;Tgfbr1;Tgif1;Trib1;Vps4b;Wtap;Zbtb21;Zbtb7a;Zc3h12c;Zeb2* |
| mmu-let-7c-5p | *1700017B05Rik;AI606181;AW549877;Abcc5;Abl2;Ankrd49;Arrdc4;Bcl2l1;Ccdc71l;Ccnf;Cd200r1;Cdk6;Chd7;Cish;Clp1;Col15a1;Cpeb4;Cyb561d1;Dtx2;Dtx4;Dusp16;Dyrk1a;Edn1;Eea1;Efhd2;Egr3;Elf4;Ergic1;Ero1l;Etnk1;Fam214b;Fbxo30;Fndc3a;Fnip1;Fnip2;Gas7;Gatm;Gbp7;Gm13889;Gpatch3;Gpr157;Gramd1c;Hic2;Hk2;Hmga1;Iglon5;Ikbke;Il10;Kcng3;Kdm3a;Klhl6;Klkb1;Lcor;Lonrf3;Mab21l3;Map4k4;Mapk6;Mgat4a;Msr1;Nab1;Nceh1;Nhlrc3;Nlrc5;Olr1;Osbpl3;Pacs2;Pias4;Pik3ip1;Plagl2;Plcxd2;Pmaip1;Pqlc2;Prdm1;Ptafr;Rab15;Ranbp2;Rbpj;Rffl;Rgs16;Rufy3;Skil;Slc20a1;Slc36a1;Slc8b1;Smim3;Socs1;Stxbp5;Tbkbp1;Tet3;Tgfbr1;Tnfaip3;Tnfrsf1b;Tor1aip2;Trib1;Tsc1;Vcpip1;Xkr8;Zfp281;Zfyve26;Zswim4* |
| mmu-miR-331-3p | *Acsl1;Aff1;Atxn7l3;B4galt5;Furin;Ikbke;Kif1a;Mllt6;Parp10;Psd4;Ptgir;Slc39a14;Socs1;Tbc1d10a;Tbc1d2;Tox4;Trim56;Wdr81;Zfp523* |
| mmu-miR-379-5p | *Ccnyl1;Efcab8;Fap;Pcgf5;Slc20a1;Vps26a* |

**Supplementary Table 10:** Downregulated DE mRNAs that are predicted targets of 4 or more upregulated conserved DE miRs

| **Down regulated DE mRNA** | **May be targeted by upregulated DE miR** | **mRNA official name** | **Also known as** | **Functions and processes** |
| --- | --- | --- | --- | --- |
| *Dip2c* | mmu-miR-139-5p, mmu-miR-721,  mmu-miR-130b-3p, mmu-miR-18a-5p | disco interacting protein 2 homolog C | mKIAA0934; 9630044M06; 2900024P20Rik | Putative transcription factor binding |
| *Dynll2* | mmu-miR-139-5p, mmu-miR-15b-5p, mmu-miR-721,  mmu-miR-130b-3p | dynein light chain LC8-type 2 | DLC8; Dlc2; DLC8b; C87222; 1700064A15Rik; 6720463E02Rik | cytoskeletal binding, motor activity, protein binding |
| *Efnb2* | mmu-miR-188-5p, mmu-miR-15b-5p, mmu-miR-721,  mmu-miR-130b-3p | ephrin B2 | Epl5; ELF-2; Eplg5; Htk-L; Lerk5; LERK-5; NLERK-1 | T cell costimulation, axon guidance, angiogenesis, cell adhesion/migration/differentiation |
| *Enah* | mmu-miR-1224-5p, mmu-miR-139-5p, mmu-miR-15b-5p, mmu-miR-721,  mmu-miR-130b-3p | enabled homolog (Drosophila) | Mena; WBP8; Ndpp1; NDPP-1 | actin cytoskeletal organization |
| *Lrp2* | mmu-miR-139-5p, mmu-miR-15b-5p, mmu-miR-721,  mmu-miR-130b-3p | low density lipoprotein receptor-related protein 2 | Gp330; Megalin; AI315343; AW536255; b2b1625.2Clo; D230004K18Rik | Hedgehog signaling pathway, organism-specific biosystem, Metabolism, organism-specific biosystem |
| *Nfib* | mmu-miR-1224-5p, mmu-miR-139-5p, mmu-miR-721,  mmu-miR-130b-3p | nuclear factor I/B | CTF; NF1-B; NFI-B; NF-I/B; 6720429L07Rik; E030026I10Rik | DNA binding, transcriptional factor |
| *Sos2* | mmu-miR-139-5p, mmu-miR-15b-5p, mmu-miR-721,  mmu-miR-130b-3p | son of sevenless homolog 2 (Drosophila) | SOS-2; mSOS-2 | lymphocyte homeostasis, regulation of small GTPase signal transduction |
| *Trim2* | mmu-miR-15b-5p, mmu-miR-721,  mmu-miR-130b-3p,  mmu-miR-18a-5p | tripartite motif-containing 2 | narf; mKIAA0517 | regulation of neuron apoptotic process |
| *Wdr50* | mmu-miR-139-5p, mmu-miR-15b-5p, mmu-miR-721,  mmu-miR-130b-3p | UTP18 small subunit processome component | Utp18; 6430513M13; 6230425C22Rik | poly(A) RNA binding |
| *Zbtb20* | mmu-miR-188-5p, mmu-miR-139-5p, mmu-miR-15b-5p, mmu-miR-721,  mmu-miR-130b-3p, mmu-miR-18a-5p | zinc finger and BTB domain containing 20 | HOF; DPZF; Oda8; ODA-8S; Zfp288; D16Wsu73e; 1300017A20Rik; 7330412A13Rik; A930017C21Rik | transcriptional repressor activity |

**Supplementary Table 11:** Candidate regulatory miRs based on miRhub analysis.

| **Candidate regulatory miR family based on DE predicted targets** | **P Value** | **miR family member whose expression is anti-correlated with predicted targets** |
| --- | --- | --- |
| **Upregulated predicted targets** |  |  |
| **miR-125a-5p/125b-5p/351/670/4319** | **0.000998** | **mmu-miR-125a-5p, mmu-miR-125b-5p** |
| miR-184 | 0.001996 |  |
| **let-7/98/4458/4500** | **0.003992** | mmu-let-7a-5p**, mmu-let-7b-5p, mmu-let-7c-5p, mmu-let-7d-5p, mmu-let-7e-5p, mmu-let-7f-5p** |
| miR-302ac/520f | 0.003992 |  |
| *miR-1611/4721* | 0.006986 |  |
| miR-760-3p/1842 | 0.007984 |  |
| **miR-126-3p** | **0.00998** | **mmu-miR-126a-3p** |
| *miR-885-5p* | 0.010978 |  |
| miR-142-3p | 0.013972 |  |
| miR-192-5p+1 | 0.01497 |  |
| miR-128/128ab | 0.016966 |  |
| miR-421 | 0.018962 |  |
| miR-670 | 0.020958 |  |
| miR-151a-3p-2 | 0.020958 |  |
| **miR-205/205ab** | **0.028942** | mmu-miR-205-5p |
| *miR-603/3571* | 0.02994 |  |
| miR-149 | 0.02994 |  |
| miR-140-3p | 0.02994 |  |
| miR-202-3p | 0.030938 |  |
| **miR-335/335-5p** | **0.032934** | **mmu-miR-335-5p** |
| miR-219-5p/508/508-3p/4782-3p | 0.04491 |  |
| miR-17/17-5p/20ab/20b-5p/93/106ab/427/518a-3p/519d | 0.046906 |  |
| miR-297ac/297b-5p | 0.048902 |  |
| **miR-23abc/23b-3p** | **0.0499** | mmu-miR-23a-3p, mmu-miR-23a-5p**, mmu-miR-23b-3p** |
| **Downregulated predicted targets** |  |  |
| miR-124/124ab/506 | 0.002994 |  |
| *miR-660* | 0.008982 |  |
| miR-361-3p | 0.022954 |  |
| *miR-4723-5p* | 0.023952 |  |
| *miR-1273g/1603* | 0.02495 |  |
| *miR-3594-5p/4685-5p* | 0.028942 |  |
| miR-453/323b-5p | 0.037924 |  |
| miR-96/507/1271 | 0.03992 |  |
| miR-191 | 0.040918 |  |
| *miR-1618/3940-3p* | 0.043912 |  |
| *miR-1324* | 0.047904 |  |

Putative key regulatory miRs based on miRhubs analysis are in bold type. miR families in italics were not represented on the array.

**Supplementary Table 12:** DE predicted targets of putative key regulatory miRs (in bold type) based on miRhubs analysis.

| **Downregulated miR** | **Upregulated DE predicted targets** |
| --- | --- |
| **mmu-miR-125a-5p, mmu-miR-125b-5p** | *Edn1, Hic2, Sema4d, Tns3, Mapkapk2, Lhfpl2, Abr, Coro2a, Lcor, Tjap1, Cdca4, Neu1, Tfec, Ppm1h, Ubtd1, Rbm7, Foxn2, Il15ra, Vps37b, Tnfaip3, Zswim4, Rps6ka2, Traf6, Fmr1, Vps4b, Papd7, Psd4, Atxn7l3, Cpeb4, Abcc5, Txnrd1, Rhoq, Cdc42ep4, Ehd1, Clec5a, Maf, Ciapin1, Rffl, Ninj1, Pde1b, Stx6, Ppm1n, Ccdc126, Rybp, Lpp, Irf1, Ptk2b, Abl2, Rxra, Slco3a1, Traf3, B4galt1, Pacs2, Camkk1, Dgkz, Larp1, Adamts4, Fam167a, Inpp1, Tgfbr1, Zbtb7a, Ereg, Arrdc4, Ptpn7, Scly, Vps37c, Nqo1, Tnfrsf1b, Plagl2, Setdb2, Gas7, Tor1aip2, Slfn5, Plekhm2, Gpc1, Mitf, Bak1, Tet3, Pik3r5, Esrra, Phf7, Lif, Ptges, Def8, Tnf, Bai1, Osbpl3, Spag9, Mcfd2, Arfgef2, Eaf1, Kcnab1, Trim14, Ptgir, Lfng, Pou2f2, Prdm1, Dtx4, Ggh, Zfp62, Lipg, Tmem38b, Fkbp15, Tmem229b, Hspb7, Mmp19, Havcr2, Phf23, Ccdc25, Rfx5, Vcpip1, Stx2, Sgpl1, Rest, Stoml1, Adora2a, Acp2, Lsm10, Parp14, Csf3, Spen, Pfkfb3, Clcn7, Usp2, Gab2, Hsdl1, M6pr, Fam169b, Mllt6, Fam46a, Homer1, Hk2, Ogfr, Zfyve26, Mgat4a, Inpp5d, St6galnac6, Cyb561d1, Itpkc, Itgb3, Naa25, Lta, Fmnl2, Smcr8, Pik3ip1* |
| mmu-let-7a-5p,  **mmu-let-7b-5p,**  **mmu-let-7c-5p,**  **mmu-let-7d-5p,**  **mmu-let-7e-5p,**  **mmu-let-7f-5p** | *Edn1, Nhlrc3, Hic2, Lcor, Rhoh, Ankrd49, Klkb1, Klhl6, Rab15, Olr1, Elf4, Tnfaip3, Zswim4, Adamts6, Gramd1c, Il10, Chd7, Efhd2, Gpatch3, Atxn7l3, Cpeb4, Abcc5, Nab1, Mafg, Marcksl1, Ccnf, Cd200r1, Rffl, Eea1, Ergic1, Rgs16, Abl2, Pacs2, Nceh1, Slc25a22, Slc26a4, Tgfbr1, Ereg, Casp7, Arrdc4, Egr3, Il6, Rnf213, Mapk6, Slc20a1, Tnfrsf1b, Plagl2, Ikbke, Ankrd12, Gas7, Enc1, Map4k4, Gatm, Crtam, Nsmce2, Tet3, A1cf, Pik3ap1, Ero1l, Ctsb, Il1a, Osbpl3, Pqlc2, Gns, Adamtsl4, Arfgef2, Msr1, Kcnab1, Slc7a11, Slc31a2, Pou2f2, Fbxo30, Prdm1, Dtx4, Dtx2, Tsc1, Pmaip1, Slc43a2, Socs1, Rufy3, Igf2r, Ccdc25, Rfx5, Vcpip1, Mab21l3, Col15a1, Fnip1, Clp1, Parp14, Cdk6, Stxbp5, Myo5a, Tbkbp1, Treml4, Trib1, Tes, Gab2, Dot1l, Slc36a1, Nlrc5, Fndc3a, Hk2, Bcl2l1, Iglon5, Pts, Ogfr, Zfyve26, Mgat4a, Xkr8, Stat2, Ptafr, Dyrk1a, Cyb561d1, Itpkc, Itgb3, Dusp16, Etnk1, Hmga1, Ranbp2, Kdm3a, Smcr8, Pik3ip1* |
| **mmu-miR-126a-3p** | *Sema4d, Ppm1h, Efhd2, Gna13, Tsc1, Pid1, Sgpl1, Plk2* |
| **mmu-miR-335-5p** | *Hic2, Sod2, Sema4d, Lhfpl2, Lcor, Rhoh, Tfec, Rbm7, Elf4, Nr4a3, Eif4e, F13a1, Fmr1, Lrp8, Ski, Cpeb4, Abcc5, Txnrd1, Slc15a3, Rhoq, Med12l, Ehd1, Pde1b, Rybp, Map4, Lpp, Enpp4, Snip1, Zbtb22, Ublcp1, Pacs2, Nceh1, Snx10, Aim1, Egr2, Tcp11l1, Slc1a2, Tgfbr1, Casp7, Egr3, Wdfy1, Slc6a19, Plagl2, Ankrd12, Orai2, Zc3h10, Ccdc88b, Papss2, Apobr, Prss46, Pik3ap1, Lif, Coro1c, Gns, Il21r, Eaf1, Slc7a11, Sdhaf1, Tbc1d10a, Tsc1, Spty2d1, Sh3bp5, Slc37a2, Tmem229b, N4bp1, Igf2r, Tollip, Tpst1, Adnp2, Trafd1, Phf23, Ifit3, Agpat9, Vcpip1, Clcn6, Rest, Clp1, Csf3, Ttc39c, Myo5a, Lipa, Ccnyl1, Trib1, Dot1l, Wdr81, Bcl2l11, Mllt6, Fam46a, Dock10, Plekhm1, Bcl2l1, Kif1c, Zfyve26, Zeb2, Cyld, Mgat4a, B4galt7, Itpkc, Spata13, Naa25, Cd274, Ttc1, Smcr8* |
| mmu-miR-23a-3p,  mmu-miR-23a-5p,  **mmu-miR-23b-3p** | *Osbpl8, Lhfpl2, Prdm2, Ccl19, Lcor, Tfec, Olr1, Elf4, Safb2, Gch1, Dgkq, Wsb1, Tnfaip3, Adamts6, Exoc3l4, Fmr1, Chd7, Vps4b, Lrp8, Atxn7l3, Cpeb4, Acsl1, Txnrd1, Elmo1, Nab1, Kif1a, Vcan, Med12l, Marcksl1, Maf, Stx6, Ccdc126, Eea1, Rybp, Lpp, Mtm1, Irf1, Ptk2b, Slc39a14, Abl2, Smad3, Fas, Gnpda1, Slco3a1, Traf3, N4bp2, Ccl7, Ccnl2, Map3k8, Slc1a2, Inpp1, Ndfip2, Casp7, Znrf2, Plekhf2, Egr3, Wdfy1, Rap2b, Lrif1, Txk, Hccs, Slc6a19, Slc20a1, Dcp2, Cdk5r1, Srfbp1, Angel2, Tor1aip2, Enc1, Map4k4, Arhgap27, Znrf1, Mitf, Tet3, S1pr2, A1cf, Nek6, Hexim1, Mcfd2, Atp6v1a, Epsti1, Atp6v1e1, Zc3h12c, Fbxo30, Ptger4, Prdm1, Trpm7, Ywhag, Tmem68, Tlr7, Blcap, Lipg, Tmem38b, Tgif1, Tmem229b, N4bp1, Whamm, Cldn1, Sirt1, Tollip, Tpst1, Mmp19, Plau, Aff1, Vcpip1, Ascc3, Tmem86a, Ube2f, Col15a1, Sgpl1, Abca1, Fnip1, Adora2a, Arid5b, Zbtb43, Myo5a, Trib1, Gab2, Ccl2, M6pr, Pml, Marcks, Slc36a1, Denr, Fmnl3, Fam46a, Dock10, Mtmr7, Fndc3a, Zeb2, Mgat4a, Ptafr, Slc9a8, Ell2, Lgals8, Etnk1, Cd69, Fmnl2, Tbc1d9, Smcr8, Pik3ip1* |

The miRs in regular font (i.e. not in bold type) are downregulated but not consensus DE.

**Supplementary Table 13.** miR families and individual miRs identified as putative miRhubs and their unique and shared DE predicted targets

| **Putative miRhub** | **DE predicted target mRNAs** |
| --- | --- |
| [let-7/98/4458/4500]  **mmu-let-7b-5p, mmu-let-7c-5p, mmu-let-7d-5p, mmu-let-7e-5p, mmu-let-7f-5p** | *Nhlrc3, Ankrd49, Klkb1, Klhl6, Rab15, Gramd1c, Il10, Gpatch3, Mafg, Ccnf, Cd200r1, Ergic1, Rgs16, Slc25a22, Slc26a4, Il6, Rnf213, Mapk6, Ikbke, Gatm, Crtam, Nsmce2, Ero1l, Ctsb, Il1a, Pqlc2, Adamtsl4, Msr1, Slc31a2, Dtx2, Pmaip1, Slc43a2, Socs1, Rufy3, Mab21l3, Cdk6, Stxbp5, Tbkbp1, Treml4,Tes, Nlrc5, Iglon5, Pts, Xkr8, Stat2, Dyrk1a, Dusp16, Hmga1, Ranbp2, Kdm3a* |
| [mir-126-3p]  **mmu-miR-126a-3p** | *Gna13, Pid1, Plk2* |
| [miR-335/335-5p]  **mmu-miR-335-5p** | *Sod2, Nr4a3, Eif4e, F13a1, Ski, Slc15a3, Map4, Enpp4, Snip1, Zbtb22, Ublcp1, Snx10, Aim1, Egr2, Tcp11l1, Orai2, Zc3h10, Ccdc88b, Papss2, Apobr, Prss46, Coro1c, Il21r, Sdhaf1, Tbc1d10a, Spty2d1, Sh3bp5, Slc37a2, Adnp2, Trafd1, Ifit3, Agpat9, Clcn6, Ttc39c, Lipa, Ccnyl1, Wdr81, Bcl2l11, Plekhm1, Kif1c, Cyld, B4galt7, Spata13, Cd274, Ttc1* |
| [miR-23abc/23b-3p]  **mmu-miR-23b-3p** | *Osbpl8, Prdm2, Ccl19, Safb2, Gch1, Dgkq, Wsb1, Exoc3l4, Acsl1, Elmo1, Kif1a, Vcan, Mtm1, Slc39a14, Smad3, Fas, Gnpda1, N4bp2, Ccl7, Ccnl2, Map3k8, Ndfip2, Znrf2, Plekhf2, Rap2b, Lrif1, Txk, Hccs, Dcp2, Cdk5r1, Srfbp1, Angel2, Arhgap27, Znrf1, S1pr2, Nek6, Hexim1, Atp6v1a, Epsti1, Atp6v1e1, Zc3h12c, Ptger4, Trpm7, Ywhag, Tmem68, Tlr7, Blcap, Tgif1, Whamm, Cldn1, Sirt1, Plau, Aff1, Ascc3, Tmem86a, Ube2f, Abca1, Arid5b, Zbtb43, Ccl2, Pml, Marcks, Denr, Fmnl3, Mtmr7, Slc9a8, Ell2, Lgals8, Cd69, Tbc1d9* |
| [miR-125a-5p/125b-5p/351/670/4319]  **mmu-miR-125a-5p, mmu-miR-125b-5p** | *Tns3, Mapkapk2, Abr, Coro2a, Tjap1, Cdca4, Neu1, Ubtd1, Foxn2, Il15ra, Vps37b, Rps6ka2, Traf6, Papd7, Psd4, Cdc42ep4, Clec5a, Ciapin1, Ninj1, Ppm1n, Rxra, B4galt1, Camkk1, Dgkz, Larp1, Adamts4, Fam167a, Zbtb7a, Ptpn7, Scly, Vps37c, Nqo1, Setdb2, Slfn5, Plekhm2, Gpc1, Bak1, Pik3r5, Esrra, Phf7, Ptges, Def8, Tnf, Bai1, Spag9, Trim14, Ptgir, Lfng, Ggh, Zfp62, Fkbp15, Hspb7, Havcr2, Stx2, Stoml1, Acp2, Lsm10, Spen, Pfkfb3, Clcn7, Usp2, Hsdl1, Fam169b, Homer1, Inpp5d, St6galnac6, Lta* |
| [let-7/98/4458/4500] and [mir-126-3p]  **mmu-let-7b-5p, mmu-let-7c-5p, mmu-let-7d-5p, mmu-let-7e-5p, mmu-let-7f-5p**  **mmu-miR-126a-3p** | *Efhd2* |
| [let-7/98/4458/4500] and [miR-335/335-5p]  **mmu-let-7b-5p, mmu-let-7c-5p, mmu-let-7d-5p, mmu-let-7e-5p, mmu-let-7f-5p**  **mmu-miR-335-5p** | *Rhoh, Nceh1, Ankrd12, Pik3ap1, Gns, Slc7a11, Igf2r, Clp1, Dot1l, Bcl2l1* |
| [let-7/98/4458/4500] and [mir-126-3p] and [miR-335/335-5p]  **mmu-let-7b-5p, mmu-let-7c-5p, mmu-let-7d-5p, mmu-let-7e-5p, mmu-let-7f-5p**  **mmu-miR-126a-3p**  **mmu-miR-335-5p** | *Tsc1* |
| [miR-125a-5p/125b-5p/351/670/4319] and [let-7/98/4458/4500]  **mmu-let-7b-5p, mmu-let-7c-5p, mmu-let-7d-5p, mmu-let-7e-5p, mmu-let-7f-5p**  **mmu-miR-125a-5p,**  **mmu-miR-125b-5p** | *Edn1, Zswim4, Rffl, Ereg, Arrdc4, Tnfrsf1b, Gas7, Osbpl3, Arfgef2, Kcnab1, Pou2f2, Dtx4, Ccdc25, Rfx5, Parp14, Hk2, Ogfr, Cyb561d1, Itgb3* |
| [miR-125a-5p/125b-5p/351/670/4319] and [mir-126-3p]  **mmu-miR-125a-5p, mmu-miR-125b-5p**  **mmu-miR-126a-3p** | *Ppm1h* |
| [miR-125a-5p/125b-5p/351/670/4319] and [let-7/98/4458/4500] and [miR-23abc/23b-3p]  **mmu-miR-125a-5p, mmu-miR-125b-5p**  **mmu-let-7b-5p, mmu-let-7c-5p, mmu-let-7d-5p, mmu-let-7e-5p, mmu-let-7f-5p**  **mmu-miR-23b-3p** | *Tnfaip3, Atxn7l3, Abl2, Tet3, Prdm1, Gab2, Pik3ip1* |
| [miR-125a-5p/125b-5p/351/670/4319] and [let-7/98/4458/4500] and [miR-335/335-5p] and [miR-23abc/23b-3p]  **mmu-let-7b-5p, mmu-let-7c-5p, mmu-let-7d-5p, mmu-let-7e-5p, mmu-let-7f-5p**  **mmu-miR-125a-5p, mmu-miR-125b-5p**  **mmu-miR-23b-3p**  **mmu-miR-335-5p** | *Lcor, Cpeb4, Vcpip1, Mgat4a, Smcr8* |
| [let-7/98/4458/4500] and [miR-335/335-5p] and [miR-23]  **mmu-let-7b-5p, mmu-let-7c-5p, mmu-let-7d-5p, mmu-let-7e-5p, mmu-let-7f-5p**  **mmu-miR-335-5p**  **mmu-miR-23b-3p** | *Elf4, Casp7, Egr3, Myo5a, Trib1* |
| [let-7/98/4458/4500] and [miR-23abc/23b-3p]  **mmu-let-7b-5p, mmu-let-7c-5p, mmu-let-7d-5p, mmu-let-7e-5p, mmu-let-7f-5p**  **mmu-miR-23b-3p** | *Olr1, Adamts6, Chd7, Nab1, Marcksl1, Eea1, Slc20a1, Enc1, Map4k4, A1cf, Fbxo30, Col15a1, Fnip1, Slc36a1, Fndc3a, Ptafr, Etnk1* |
| [miR-125a-5p/125b-5p/351/670/4319] and [miR-335/335-5p] and [miR-23abc/23b-3p]  **mmu-miR-125a-5p, mmu-miR-125b-5p**  **mmu-miR-335-5p**  **mmu-miR-23b-3p** | *Lhfpl2, Tfec, Fmr1, Txnrd1, Rybp, Lpp, Tmem229b, Fam46a* |
| [miR-125a-5p/125b-5p/351/670/4319] and [miR-335/335-5p]  **mmu-miR-125a-5p, mmu-miR-125b-5p**  **mmu-miR-335-5p** | *Rbm7, Rhoq, Ehd1, Pde1b, Lif, Eaf1, Phf23, Rest, Csf3, Mllt6, Naa25* |
| [miR-125a-5p/125b-5p/351/670/4319] and [mir-126-3p] and [miR-335/335-5p]  **mmu-miR-125a-5p, mmu-miR-125b-5p**  **mmu-miR-126a-3p**  **mmu-miR-335-5p** | *Sema4d* |
| [miR-335/335-5p] and [miR-23abc/23b-3p]  **mmu-miR-335-5p**  **mmu-miR-23b-3p** | *Lrp8, Med12l, Slc1a2, Wdfy1, Slc6a19, N4bp1, Tollip, Tpst1, Dock10, Zeb2* |
| [miR-125a-5p/125b-5p/351/670/4319] and [miR-23abc/23b-3p]  **mmu-miR-125a-5p, mmu-miR-125b-5p**  **mmu-miR-23b-3p** | *Vps4b, Maf, Stx6, Ccdc126, Irf1, Ptk2b, Slco3a1, Traf3, Inpp1, Tor1aip2, Mitf, Mcfd2, Lipg, Tmem38b, Mmp19, Adora2a, M6pr, Fmnl2* |
| [miR-125a-5p/125b-5p/351/670/4319] and [mir-126-3p] and [miR-23abc/23b-3p]  **mmu-miR-125a-5p, mmu-miR-125b-5p**  **mmu-miR-126a-3p**  **mmu-miR-23b-3p** | *Sgpl1* |
| [miR-125a-5p/125b-5p/351/670/4319] and [let-7/98/4458/4500] and [miR-335/335-5p]  **mmu-miR-125a-5p, mmu-miR-125b-5p**  **mmu-let-7b-5p, mmu-let-7c-5p, mmu-let-7d-5p, mmu-let-7e-5p, mmu-let-7f-5p**  **mmu-miR-335-5p** | *Hic2, Abcc5, Pacs2, Tgfbr1, Plagl2, Zfyve26, Itpkc* |

The miR or miR family identified as candidate regulatory miRs according to miRhub analysis are in brackets. The DE miRs that are putative miRhubs are in bold type.


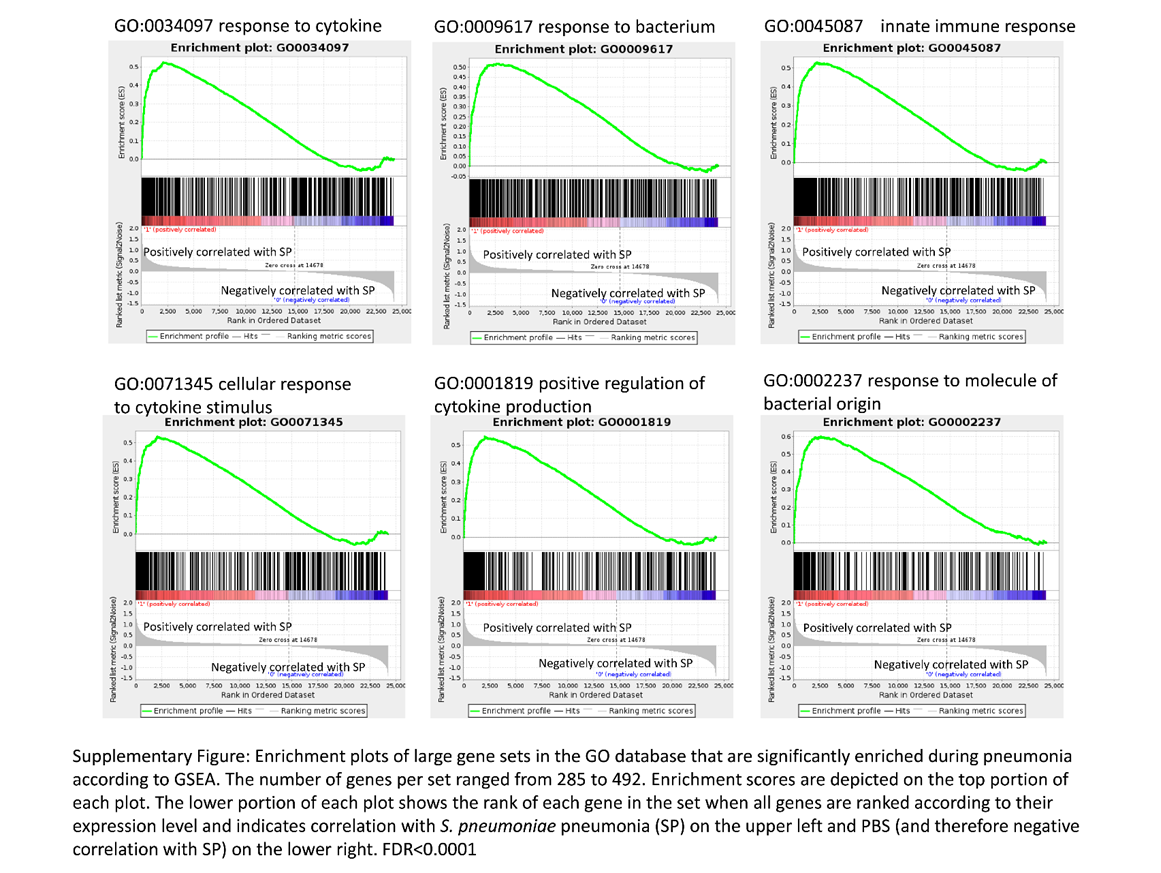

Supplement: Supplementary file 1 — Supplementary tables and figure [file 41598_2017_11638_MOESM1_ESM.doc]
